# Silicone-Foam Passive Air Samplers for Combined Target and Nontarget Chemical Profiling and Toxicity Assessment of Airborne Exposomes

**DOI:** 10.1021/acs.est.5c16613

**Published:** 2026-02-10

**Authors:** Adrià Sunyer-Caldú, Hongyu Xie, Bénilde Bonnefille, Foteini Raptopoulou, Edouard Pesquet, May Britt Rian, Daniel Schlesinger, Michael Norman, Young June Jeon, Boram Kim, Seung-Bok Lee, Ji Eun Lee, Jean Froment, Stefano Papazian, Jonathan W. Martin

**Affiliations:** † Department of Environmental Science, Science for Life Laboratory, 7675 Stockholm University, Stockholm 10691, Sweden; ‡ National Facility for Exposomics, Metabolomics and Exposomics Platform, Science for Life Laboratory, Stockholm University, Solna 171 65, Sweden; § Arrhenius Laboratories, Department of Ecology, Environment and Plant Sciences (DEEP), Science for Life Laboratory, Stockholm University, Stockholm 10691, Sweden; ∥ SLB-Analys, Environment and Health Administration, City of Stockholm, Stockholm 104 20, Sweden; ⊥ Chemical & Biological Integrative Research Center, Biomedical Research Division, Korea Institute of Science and Technology, Seoul 02792, Republic of Korea; # Center for Sustainable Environment Research, Climate and Environmental Research Institute, Korea Institute of Science and Technology, Seoul 02792, Republic of Korea; ∇ Department of Environmental Chemistry and Health Effects, NILU, Kjeller NO-2027, Norway

**Keywords:** PDMS, passive sampling, nontarget analysis, particulate matter, exposomics, air pollution, chemical profiling, toxicity

## Abstract

Polluted air is a major global health risk factor, yet the chemical composition and toxicity of airborne gases and particles remain underexplored due to their complexity and difficulties in sampling. We recently introduced how polydimethylsiloxane (PDMS) foam—or silicone foam—can be synthesized for passive air sampling, enabling simple and cost-effective nontarget chemical profiling of indoor air. Here, we demonstrate expanded applications, indoors and outdoors, with commercial PDMS-foam, including for: (i) wide-scope target analysis of >220 priority substances by quantitative liquid- and gas chromatography-high-resolution mass spectrometry, (ii) microscopic characterization and nontarget profiling of accumulated fine particles, and (iii) effect-guided discovery of harmful substances, combining toxicological data with nontarget analysis in silico. Median method quantification limits were 0.12 ng/mL, 90% of target analytes had absolute recoveries between 70 and 130%, and hazardous substances were discovered, including ethylene glycols, insecticides, and UV filters. Microscopy revealed the accumulation of abundant fine particles, and the automated characterization of the fluorescent fraction revealed that most were <4 μm. Extracts from outdoor samples reduced human lung cell viability, and multivariate modeling flagged families of potentially toxic substances in a virtual effect-directed analysis. PDMS-foam disks require field calibration to determine their linear sampling rate(s), but current results and applications establish PDMS-foam as a multimodal passive sampler, enabling integrated chemical quantitation, toxicological analysis, and molecular discovery in air.

## Graphical abstract

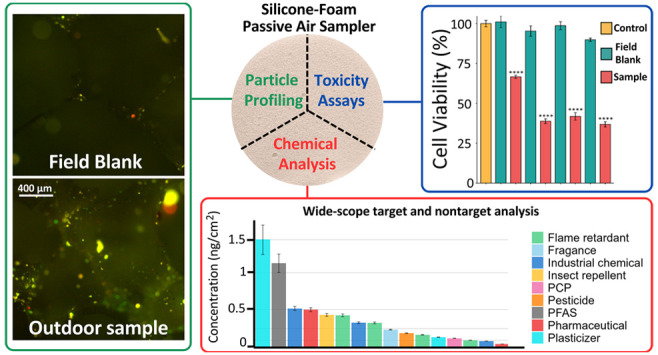

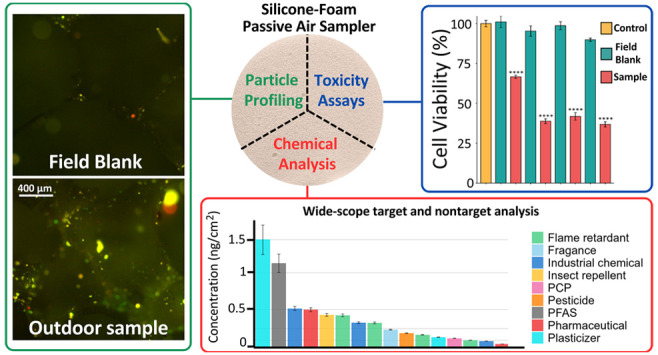

## Introduction

Air pollution stands as one of the most pervasive environmental global health threats, contributing to a significant burden of disease.^1^ The World Health Organization (WHO) estimates that approximately 7 million premature deaths are attributable to air pollution annually; thus, the airborne exposome is a leading environmental risk factor for both mortality^2^ and morbidity.^1^ Air pollution is a complex heterogeneous mixture encompassing gases, fine particulate matter (PM), and a diverse array of volatile and semivolatile organic compounds (SVOCs), many of which have been associated with severe health conditions, such as respiratory diseases, cardiovascular diseases, and cancer.^3^ Chronic exposure to airborne fine PM is a major contributor to the health impacts, having been directly linked to cardiovascular and respiratory diseases, systemic inflammation, and premature death.^4,5^ Despite its clear significance for exposure and adverse health consequences, the airborne chemical exposome and its toxicity remain poorly characterized due to the inherent complexities and dynamics of air,^6^ as well as practical limitations of sampling techniques.^7^

Past workflows aimed at measuring ambient airborne SVOCs have primarily focused on specific classes of compounds, for example, the polycyclic aromatic hydrocarbons (PAHs)^8^ or polychlorinated biphenyls (PCBs).^9^ Most studies of airborne contaminants have prioritized the analysis of nonpolar semivolatile compounds that are readily analyzed by gas chromatography (GC), with significantly fewer studies^10^ employing liquid chromatography (LC) to detect the more polar fraction of airborne contaminants. This gap is critical, as many priority airborne pollutants—such as oxy-PAHs, current-use pesticides, and certain per- and polyfluoroalkyl substances (PFAS)—are thermally unstable, nonvolatile, or have acidic or basic functionalities that make them unsuitable for sensitive GC analysis. Expanding LC-based approaches can enhance chemical-space coverage and allow broader exposure assessments, particularly for airborne PM which contains a major fraction of polar and water-soluble substances.^11^ While targeted approaches to air analysis offer valuable insights into priority substances, this only informs on a small fraction of total airborne chemical exposures. Nontarget mass spectrometry approaches show promise in uncovering emerging airborne contaminants,^12^ but substantial challenges remain due to the available air sampling media. Conventional passive sampling media for airborne organic contaminants, such as polyurethane foam (PUFs), have been used successfully in previous studies for nontarget or suspect screening applications^12,13^ and have demonstrated the stability of collected chemicals over time.^14,15^ However, the PUF sampler undergoes oxidation during deployment,^16–18^ which we hypothesize will introduce mixtures of polar organic degradation products into the associated extracts that will confound subsequent toxicity testing and the accurate characterization of airborne organic substances by nontarget analysis.

To minimize such molecular interferences introduced from the sampling media, a promising new alternative air sampling media is the use of silicone-foam, also known as polydimethylsiloxane (PDMS)-foam.^19^ This material is inexpensive and simple to deploy like PUF passive samplers but offers an inherent advantage for nontarget analysis due to its polysiloxane backbone of alternating silicon and oxygen atoms; any oxidation or degradation products from the PDMS media will also contain silicon and can thereby be distinguished in nontarget analysis from environmental analytes. PDMS-foam can also be thermally cleaned to reduce chemical background and has been shown to be relatively clean from chemical interferences, with the exception of short PDMS-oligomers.^19^ Solid PDMS has previously been applied in personal exposure monitoring, such as in the FreshAir^20^ or silicone wristbands,^21^ due to its ability to absorb a broad range of airborne SVOCs by passive diffusion. Compared to smooth PDMS, the rough and increased surface area of PDMS-foam creates greater potential for impaction and collection of fine particles—the key airborne exposure pathway leading to adverse health outcomes. The linear uptake of a broad spectrum of SVOCs on PDMS-foam, including organic acids and bases, suggests that PDMS-foam effectively captures both the gas- and particle-phase of air over several weeks of indoor deployment.^19^

Building on the preliminary results from PDMS-foam disks synthesized in our lab,^19^ and their strong performance for nontarget analysis of indoor air, here we explore the use of commercially available PDMS-foam and demonstrate advances in its application to monitoring and characterization of the airborne exposome using advanced nontarget tools, such as feature-based molecular networking (FBMN).^22^ We present new evidence—combining microscopy and aqueous extraction—for the collection of fine PM on the surface of indoor and outdoor deployed PDMS-foam and validate quantitative methods for wide-scope target analysis of organic contaminants in various extracts, employing both LC- and GC- high-resolution mass spectrometry (HRMS). Building on our preliminary study, which only semiquantified a few PCBs, we developed and validated two complementary workflows here—one for polar organic compounds and another for nonpolar compounds—covering >220 target chemicals. In a series of ambient passive air samples from an East-Asian megacity, we also demonstrate toxicity testing of the polar extracts in human cells and show how the resulting toxicological data can be combined with nontarget data to discover and characterize potentially toxic families of airborne substances.

## Materials and Methods

### PDMS-Foam Sampling Disks and Optimization of Cleaning Protocol

In previous work, we custom-synthesized PDMS-foam disks from silicon elastomer,^19^ but here commercially available PDMS-foam sheets were used as passive air samplers and cleaned prior to deployment to minimize background signals. For some applications, smaller subdisks were produced from the larger disks for method optimization (see Deployment 1). The predeployment cleaning protocol was optimized in this study to ensure effective removal of residual contaminants while maintaining sampler integrity, minimizing PDMS-oligomer background in POC extracts, and enhancing analytical sensitivity and signal stability in extended GC-HRMS sequences (Figures S1 and S2). Various conditions, including vacuum oven temperature, baking time, extraction solvent, extraction temperature, and disk size, were evaluated (Table S1, Figure S3). Detailed protocols are provided in Section S1 of the Supporting Information (SI).

### Design of Wide-Scope Target Analyses

The aim was an analytical method with broad coverage of analytes with diverse physicochemical properties and structures (Figure S4). Two lists of wide-scope target analytes were selected based on the occurrence of chemical contaminants reported in air and PM in previous studies.^11,19,23^ The complete list encompassed 118 polar analytes (Table S2; LC) and 106 nonpolar analytes (Table S3; GC). Stock solutions of these 224 analytes were used for quantitative monitoring but also in method development to understand any limitations of the analytical method’s chemical-space coverage. All analytical standards used in this study, including those used for retrospective confirmation of tentative annotations, are reported along with supplier information, CAS number, purity, and other details in Table S4. Many isotope-labeled internal standards (IS) (*n* = 73) covering various chemical classes were used to monitor and correct for extraction and analytical variability in the optimized LC and GC methods (Table S5).

### Passive Air Sampling Studies with PDMS-Foam

#### Deployment 1 (Indoor, April–May 2023)

The goal of this first sampling experiment was to obtain representative samples for optimizing and validating the GC and LC analytical methods. Small PDMS-foam disks (*n* = 96) and field blanks (*n* = 12) were cleaned and deployed for 40 days in an office at Stockholm University. Disks were suspended from the ceiling using clean aluminum wire under a clean aluminum cover to minimize deposition of larger dust particles (Figure S5a). After collection, samples were individually wrapped in two layers of aluminum foil and sealed in zip-lock plastic bags, stored at −20 °C until extraction. Field blanks were briefly exposed and handled identically to the samples, including wrapping and storage. Office activities remained unchanged during the deployment time.

#### Deployment 2 (Indoor/Outdoor, March-April 2023)

The goal of this deployment was to characterize the PDMS-foam’s ability to passively capture airborne fine particles, which were characterized using microscopy and chemical analysis of water extracts. The large PDMS-foam disks (*n* = 3) and corresponding field blank (*n* = 1) were deployed inside double bowl housings (Tisch Environmental, TE-200-PAS) for 42 days at an urban air quality station (SLB-analys, East Sweden Air Quality Management Association) on a rooftop (20 m above street level) in central Stockholm, Sweden (59°18′56.89″N, 18°3′17.19″E) (Figure S5b, and S6). In parallel, a second set of large PDMS-foam disks (*n* = 3) and a field blank (*n* = 1) were deployed in indoor housings (Tisch Environmental, TE-300-PAS) during the same 42-day period in an office at Stockholm University, next door to the site of Deployment 1 (Figure S5c). For the indoor PDMS-foam disks only, each triplicate and field blank were subsampled on days 1, 21, and 42 by a cork borer (subsamples of 1.5 cm thickness × 2.5 cm Ø) (Figure S7b).

#### Deployment 3 (Outdoor, October 2022-January 2023)

The goal of these ambient deployments was to test a methodology for evaluating the cytotoxicity of deployed PDMS-foam extracts in human lung fibroblasts and to integrate these data with wide-scope profiling and nontarget analysis. Large PDMS-foam disks (*n* = 7), each with a time-paired field blank (*n* = 7), were deployed in double bowl housings (Tisch Environmental, TE-200-PAS) on the rooftop of the Korea Institute of Science and Technology (KIST) building in Seoul, Republic of Korea (37°33′57.6″N, 126°58′42.24″E) (Figure S5d), each for approximately 2 months.

### PDMS-Foam Extractions for HRMS Analyses

Three separate PDMS-foam extraction methods were developed, each applied to a separate quarter of the sampling disk (or to separate disks in the case of small disks) to cumulatively isolate a wide range of substances in three complementary solvents: (i) polar organic chemicals (POCs) extractable by methanol, (ii) nonpolar organic chemicals (NPOCs) extractable by hexane, and (iii) water-soluble organic chemicals (WSOCs). Each fraction was analyzed using either LC- or GC-HRMS, depending on the polarity. Detailed extraction procedures, solvent compositions, and optimization parameters are provided in the SI (Section S2), and information on IS is listed in Table S5. Further details on method validation parameters are described in Section S3. Details of QA/QC, such as precleaning protocols and method blank procedures, are described in Section S4. A summary of method applications across deployments is given in Figure S7.

### LC- and GC-HRMS Analyses

Detailed instrumental parameters are listed in Section S5. Briefly, POC extracts were analyzed using an Acquity BEH C18 column (Waters) at 40 °C with a 0.4 mL/min flow on an Ultimate 3000 UPLC system (Thermo Scientific). HRMS acquisition was performed on a Q Exactive Orbitrap HF-X (Thermo Scientific), with separate injections (10 μL) for acquisitions in positive and negative electrospray ionization modes (ESI+ and ESI−). LC gradient conditions for both ionization modes are listed in Table S6. NPOC extracts were separated on a DB-5MS column (Agilent) using a TRACE 1300 Series GC system (Thermo Fisher Scientific) with helium as carrier gas at 1 mL/min, and 2 μL volume injection using a PTV splitless injector. The HRMS acquisition was performed on a Q Exactive Orbitrap (Thermo Fisher Scientific) operating with electron ionization (EI, 70 eV) in full scan mode (40–750 *m*/*z*) at a nominal resolution of 60,000 FWHM.

For WSOCs, 1 mL of aqueous extract was injected (separately for ESI+ and ESI−) to online solid-phase extraction (SPE), using two serially connected SPE columns (Thermo Scientific Hypersil GOLD PFP and Hypersil GOLD aQ), before back-flushing a separation on the same C18 analytical column as for POCs. The online SPE columns were eluted with the analytical mobile phase, using gradient conditions in Tables S7 and S8. HRMS acquisition also followed the same parameters as those for POCs.

Target analysis was performed by using TraceFinder 5.1 (Thermo Scientific) for data review and peak integration. For quantification, only analytes with peak areas at least three times higher than the corresponding field blank(s) were considered. For nontarget analysis, preprocessing of raw data was performed in MS-DIAL (v.4.9)^24^ to identify features based on retention time (RT), peak intensity, accurate mass (MS1), and spectral (MS2) fragmentation. Feature lists were exported from MS-DIAL, filtering for peak areas at least 10-fold higher (LC) or 5-fold higher (GC) in samples compared with the average area in the field blanks. All data were normalized by ISs and blank subtracted (see Section S6). Additional processing details are described in Section S7.

Nontarget molecular annotations were performed by comparing spectra of extracted features to spectral libraries^25–27^ and in-house libraries. Confidence levels for LC annotations followed the Schymanski scale,^28^ while GC annotations adhered to the Koelmel scale.^29^ Level 2 annotations (based on spectral matching) were accepted when the MS-DIAL total identification score exceeded 700—using dot-product scores for LC-HRMS and reverse match factors for GC-HRMS—and for GC, only if the feature’s Kovats retention index was within 50 units of the reference value. For LC features, molecular formulas were calculated using SIRIUS,^30^ Section S8. Features were then clustered into molecular networks in GNPS, based on MS2 spectral similarity with criteria of cosine-score >0.65 (details in Section S9).^22,31^ Structural annotations, tailored to substances in PubChemLite for Exposomics,^32^ were performed using the NAP workflow, which reranks candidates initially generated by MetFrag using network topology.^33,34^ To increase confidence in annotations, we manually reviewed selected NAP-predicted candidates against reference MS2 spectra in MassBank and mzCloud.^35^ When fragmentation patterns matched, features were upgraded to Level 2b identification. Otherwise, they were discarded or retained as lower-confidence (Level 3) annotations. All nontarget features with annotations at Level 2 in GC-HRMS were semiquantified by normalization to the closest eluting internal standard followed by blank subtraction, with full details provided in Section S10.

### Microscopy Characterization of PDMS-Foam

PDMS-foam surfaces were evaluated microscopically to characterize particle capture. Autofluorescence and brightfield images were acquired using an inverted epifluorescence microscope, and semiautomated image processing was performed in ImageJ to identify and measure particles.^36^ Metrics included particle area, density, and shape descriptors, such as circularity and convexity. Further details of the imaging and processing workflow are provided in Section S11.

### Cytotoxicity and Cellular ROS

From Deployment 3, samples and corresponding field blanks were tested for cytotoxicity and cellular production of reactive oxidant species (ROS). Briefly, one-twelfth of the large PDMS-foam disks (surface area = 31.0 cm^2^) were extracted as described in Section S4. Cells were exposed for 48 h to 2% of the original extract concentration. Cell viability was measured using the CCK-8 assay, and ROS levels were quantified with a fluorescent ROS detection kit relative to those of untreated controls. All experiments included the corresponding field blanks. Detailed protocols and calculations are provided in Section S12.

### Statistical Analysis

Details on statistical analysis are provided in Section S13.

## Results and Discussion

### NPOC Extraction and Validation

To minimize the background of PDMS-oligomers in NPOC extracts, the optimized protocol used PLE with hexane at 40 °C and a final evapoconcentration to 1 mL. Validation of the NPOC analysis by GC-HRMS (Table S9) demonstrated robust performance with the majority of target analytes (94%) having absolute recoveries in the range of 70–130%, and the remaining (6%) still being between 43 and 140%. Median recoveries were 96% for the low spike (1 ng/mL) and 88% for the high spike (10 ng/mL), with median precisions of 15% RSD for both levels (Figure S8). Phthalates posed challenges due to elevated background in the NPOC method and were therefore only analyzed in the POC method by LC-HRMS. Matrix effects were moderate, in the range of 60–250% with a median of 107% (where 100% indicates no matrix effect), thus primarily causing signal enhancement rather than suppression (Figure S9). Consistent with the NPOC results, matrix-matched calibration was applied for POCs, yielding excellent linearity (R^2^ ≥ 0.9699) over a wide concentration range, up to 200 ng mL in extract, with a median R^2^ of 0.9992. (Figure S10). MLOQs normalized to sampler surface area (ng/cm^2^) are also provided in Table S9.

### POC Extraction and Validation

Unlike the analysis of NPOC extracts by GC-HRMS, background PDMS-oligomers are not ionizable by ESI and hence showed no apparent impact on the analysis of POC extracts by LC-HRMS. Several POC extraction methods were tested (orbital shaker, ultrasound bath, or PLE), and PLE was selected as the preferred technique because it was able to contain the expansion of the PDMS material in organic solvent. Four organic solvents (MeOH, acetonitrile, ethyl acetate, or 50:50 hexane:acetone) were evaluated for POC extraction based on absolute analyte recoveries at 1 ng/mL. The extraction solvent was critical (Figure S11), with recovery trends following solvent polarity; hexane: acetone and ethyl acetate performed poorly, while acetonitrile improved recoveries but remained suboptimal for several industrial chemicals, pharmaceuticals, and sulfonic PFAS. MeOH gave the best recoveries for most analytes, with the exception of certain pharmaceuticals (Figure S12). However, because chromatographic peak shapes were suboptimal when injecting pure MeOH (Figure S13a), final extracts were diluted 50:50 with water to improve peak shapes, sensitivity, and recoveries (Figure S13b,c).

Validation of the optimized POC method by LC-HRMS (Table S10) demonstrated strong performance, with median recoveries at 1 and 10 ng/mL of 90% (27–149%) and 91% (61–114%), respectively (Figure S8). Slightly lower recoveries at 1 ng/mL were due to blank analyte levels, thereby requiring the subtraction of the signal from the spike. Across both spiking levels, 90% of analytes had good recoveries in the range of 70–130%, highlighting method performance across a diverse range of analyte properties and structures. Matrix effects were minimal (median 90%, range 59–114%, i.e., 100% indicates no effect), and with 97% of analytes showing less than 30% matrix suppression relative to standards in solvent (Figure S14). Matrix-matched calibration was used, and linearity was excellent (*R* ^2^ ≥ 0.9699), and median *R* ^2^ was 0.9992 over a wide concentration range, up to 200 ng/mL in extract. POC method sensitivity was high, with a median MLOQ of 0.08 ng/mL in extract (range 0.005–6.5 ng/mL), and 93% of analytes had MLOQs ≤ 1 ng/mL in extract (Figure S15). MLOQs normalized to sampler surface area (ng/cm^2^) are also provided in Table S10. Higher MLOQs for certain plasticizers, preservatives, organophosphate flame retardants, and PFAS were likely attributable to their higher blank levels rather than instrumental limitations.

#### Wide-Scope Target and Nontarget Analysis of PDMS-Foam (Deployment 1)

In our previous work, we demonstrated the compatibility of PDMS-foam passive samplers with nontarget analysis and their linear uptake over weeks for a wide range of known and unknown analytes in indoor air.^19^ Here, we utilize the PDMS-foam samplers (triplicate samples and field blank, Deployment 1) to demonstrate a combination of wide-scope target analysis for >220 priority substances and nontarget analysis of airborne substances by both GC- and LC-HRMS; a range of target detections, concentrations, and nontarget discoveries are reported in sections below.

### Target Analysis

POC and NPOC workflows (Figure S16) resulted in 67 target analyte quantifications, including 46 NPOCs quantified by GC-HRMS (see Table S11) and 21 POCs quantified by LC-HRMS (see Table S12). To facilitate future conversion to air concentrations, concentrations are reported here on a surface area basis of the PDMS-foam disks (ng/cm^2^); the passive uptake rates (i.e., m^3^ cm^–2^ d^–1^) are currently being calibrated in a field study, which will eventually allow conversion to air concentrations (e.g., ng/m^3^). Concentrations in the extract (ng/mL) are also provided in Tables S11 and S12. Target analytes showed relatively high reproducibility across triplicates (Figure S18), with NPOCs showing marginally higher RSD% (median of 7.8%) than POCs (median = 5.3%). The only target analytes with RSD% > 35% were dioctyl and diisobutyl phthalate in GC, due to their relatively higher background levels in the field blank.

Target NPOCs (range < MLOQ-995 ng/cm^2^) were present at up to 100-fold higher concentrations compared to any POC (range < MLOQ-1.5 ng/cm^2^, see Figure S17). Three target analytes, i.e., tributyl phosphate (0.4 ng/cm^2^ NPOC, 0.3 ng/cm^2^ POC), DEET (0.4 NPOC, 0.4 POC), and tris­(2-butoxyethyl) phosphate (0.1 NPOC, 0.1 POC)—were quantified by both GC and LC methods and at similar concentrations within 25%. Among the analytes detected via GC-HRMS, several widely used phthalate plasticizers, e.g., diethyl-, dibutyl-, bis­(2-ethylhexyl)-, and dioctyl-phthalates, were found at the highest concentrations, consistent with previous reports of various phthalates in indoor dust^37–39^ and air.^40,41^ Their endocrine-disrupting properties and potential for reproductive or developmental toxicity make phthalates an ongoing concern for human health.^42–44^ Several PCB congeners were also detected at high concentrations, ranging between 0.1 and 15.6 ng/cm^2^. Despite being banned in Europe since 2001,^45^ their persistence and presence in old construction remains a source of human exposure. PCBs have been linked to endocrine disruption,^46^ immune suppression and developmental toxicity,^47^ and are known to biomagnify^48^ and persist in the human body.^49^ Three phosphate flame retardants, namely triphenyl phosphate, tributyl phosphate, and tris­(2-butoxyethyl) phosphate, were quantified at 0.42 ± 0.02, 0.32 ± 0.01, and 0.1 ± 0.001 ng/cm^2^, respectively. These flame retardants have been previously reported in indoor air^50–52^ and dust.^53^ Fourteen PAHs were detected at lower concentrations ranging from 0.01 to 0.37 ng/cm^2^, demonstrating the method’s sensitivity for trace-level analysis. Of the 16 PAHs regulated by the EU and US EPA for their carcinogenic and toxic properties,^54,55^ six were detected here (phenanthrene, fluoranthene, benzo­[*a*]­pyrene, acenaphthene, pyrene, and fluorene), underscoring their continued relevance for airborne exposomics. Notably, all occupants of the office were nonsmokers, but ambient levels of PAHs can originate from infiltration of outdoor sources (e.g., traffic, industrial emissions, and fossil fuel combustion).^56^

Among POCs, several persistent mobile pollutants were detected and quantified in air by the LC-HRMS method. For instance, the industrial chemical and hazardous air pollutant, 4-nitrophenol,^57^ previously classified as very persistent and very mobile in water,^58^ was detected at 10.7 ± 0.03 ng/cm^2^. While commonly reported in water, soil, and biofluids,^59,60^ its occurrence in ambient air has primarily been linked to its presence on PM.^61^ Trifluoroacetic acid (TFA), a highly persistent and mobile PFAS, was detectable in these indoor samples above blank levels (1.14 ± 0.05 ng/cm^2^ after blank subtraction). As a terminal transformation product of many trifluoromethyl-containing compounds, TFA accumulates in surface water and groundwater,^62^ and its persistence raises concerns for long-term environmental and human exposure.^58^ We are not aware of any previous report of TFA in indoor air, but it is present in ambient air^63^ and is known to partition between the gas- and particle-phase.^64^ Notably, other targeted PFAS were not detected above the MLOQ in these indoor samples from Deployment 1. DEET, a widely used mosquito and insect repellent approved as a biocide in Europe^65^ and the USA,^66^ was consistently quantified in Deployment 1 samples (0.43 ± 0.02 ng/cm^2^). While DEET has been detected in water, sediment, biota, and human biospecimens,^67–75^ reports of its occurrence in air remain limited. It has been frequently detected in wearable devices such as silicone wristbands in studies from Peru^76^ and USA,^77^ despite reports indicating DEET is not considered persistent in air.^78^ However, to the best of our knowledge, no data are available from air passive samplers.

Icaridin, a common DEET alternative, was also detected (0.19 ± 0.004 ng/cm^2^), representing the first report of its presence in air to our knowledge. Given that sampling took place in Stockholm during mosquito-free months (April–May), the detected levels may reflect background contamination or significant persistence from previous summer use. Octocrylene, a UV filter widely used in sunscreens and plastics,^79^ is also reported here for the first time as an indoor air pollutant, detected at 2.6 ± 0.002 ng/cm^2^. Its presence indoors is likely to originate from emissions from plastics, textiles, or paints, as described in previous studies of indoor dust^80^ and ambient air.^81^ With significant bioaccumulation potential^82^ and frequent detection in water, sediments, human biofluids, and indoor dust,^79,82–87^ the airborne exposure sources and effects of octocrylene warrant further study.

### Nontarget Analysis and FBMN Clusters

After feature extraction and blank filtration, LC-HRMS analysis of POCS yielded 8663 features (ESI+ and ESI– combined), while GC-HRMS analysis of NPOCs yielded 526 features (Figure S19). Spectral-library matching resulted in the initial annotation of 82 LC-HRMS features (64 in ESI+, 18 in ESI−) and 56 GC-HRMS features. Despite LC-HRMS extracting 16× more features than GC-HRMS, the annotation rate was significantly higher for GC-HRMS (11 vs 0.01%), partly reflecting the library size and added value of Kovats retention indexing; however, many LC-HRMS features may represent transformation products of commercial chemicals that have not been previously synthesized, and thus cannot be present in spectral databases. After manual curation of the annotations and removal of redundancies or target analytes, 34 LC-HRMS and 33 GC-HRMS features were confidently assigned at level 2 (Table S13; PCBs in Table S14); see spectral matches in Figures S20 (LC) and S21 (GC) and representative examples of aligned chromatographic peaks from samples and blanks shown in Figures S22 (LC) and S23 (GC). Of 23 authentic standards later acquired to confirm Level 2 annotations, 21 successfully identified the substance at Level 1 confidence,^28,29^ including five that were doubly confirmed by both GC and LC, resulting in a 91% confirmation rate.

Five compounds, including three organophosphate flame retardants and two plasticizers (one phthalate and one sulfonamide), were identified by both LC and GC, thereby reinforcing confidence in their annotation. Given the high number of PCBs detected by target analysis (*n* = 21), suspect screening of other PCBs was conducted using theoretical masses and retention data. This resulted in the tentative identification of 40 additional PCB congeners, among which 10 were later confirmed (Level 1) by authentic standards; 30 suspect PCBs remained annotated at level 2 based on spectral matches and plausible RI (Figure S24, Table S14). In total, 61 PCB congeners were identified: 23 through target analysis, and 38 through suspect/nontarget screening, including 8 at Level 1 and 30 at Level 2.

Semiquantified concentrations for Level 2 GC-HRMS annotations (Tables S14 and S15) ranged from 0.0017 to 7.2 ng cm/cm^2^, which is overall lower than those measured in the target analysis (up to 995 ng/cm^2^). Among nontarget GC-HRMS annotations, diphenyl sulfone, a high-production-volume industrial compound confirmed with an authentic standard, exhibited the highest concentration, followed by an ethylene glycol derivative and benzyl butyl phthalate. Several PCB congeners were also frequently detected in samples but largely absent from field blanks, underscoring both the strong uptake of PDMS-foams and the sensitivity of the analytical workflow. Notably, the semiquantified features in GC-HRMS spanned a wide chemical space—including plasticizers, solvents, pyrrolidones, sulfonamides, and fragrances, demonstrating the multimodal capability of PDMS-foam to capture structurally diverse airborne contaminants beyond the targeted list.

The reproducibility of internal standard-normalized nontarget feature areas was also evaluated after internal standard normalization and compared to target analytes; the reproducibility among nontarget features in the triplicate samples was lower. The median RSD for all POC features was 23.7 and 17.9% for all NPOC features (Figure S25). It is important to note that automated peak integration performed in MS-DIAL can negatively affect these results, and manual curation was not possible due to the high number of features in the dataset (i.e., 7099 features).

FBMN is a technique that enhances and leverages confident annotations by clustering putatively structurally related unknown compounds based on mass spectral similarities. Overall, 2197 molecular clusters were present in the LC-HRMS dataset in ESI+ (Figure 1a), 333 in ESI–, and 155 by GC-HRMS (Figure 1d). Clusters containing features annotated by traditional nontarget analysis—which groups neighboring features based on shared spectral characteristics, were further curated. Features with distinct retention times (RTs) were considered structurally distinct (i.e., not adducts or in-source fragments) and manually compared to spectral databases. Using the integrated FBMN and NAP workflow for these triplicate indoor air samples led to 20 additional annotations (Level 2b), including major clusters of phthalates and poly­(ethylene glycol)­s (PEGs), in addition to a smaller but notable cluster of organophosphate flame retardants (OPFRs), all of which are discussed below.

**Figure 1:**
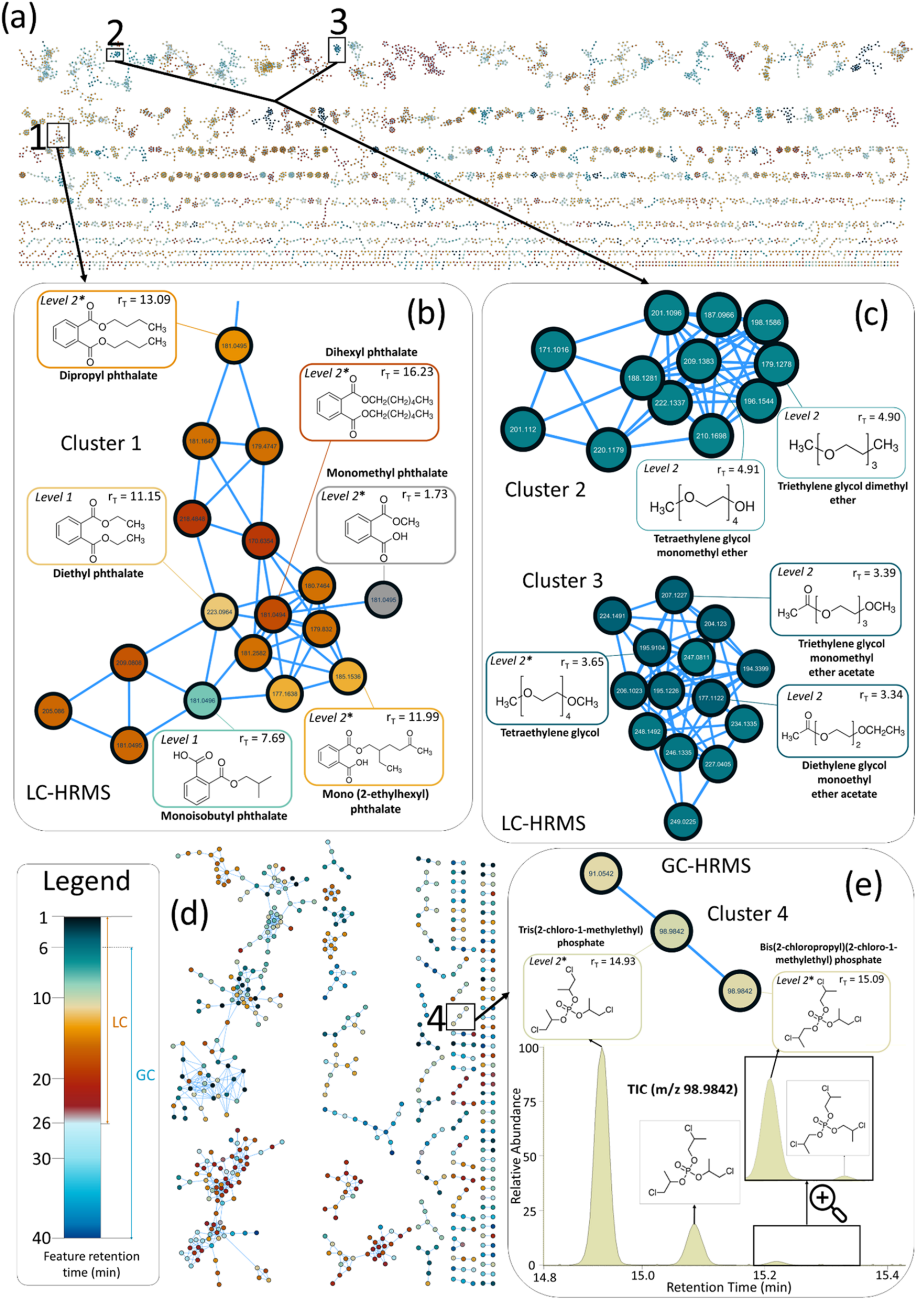
PDMS-foam molecular networking for enhanced chemical identification. The complex mixture of chemicals collected by passive sampling with PDMS-foam disks deployed indoors (Deployment 1) and detected with nontarget analysis can be visualized in molecular networks, whereby putatively structurally similar substances are clustered based on spectral similarity. (a) Example showing the complete FBMN obtained from LC-HRMS (ESI+) analyses, and zoom-in on (b) Cluster 1: phthalates, (c) Clusters 2 and 3: polyethylene glycol derivatives. (d) Complete FBMN obtained from GC-HRMS (EI) analyses, and zoom-in on (e) Cluster 4: two tris­(chloropropyl) phosphate (TCPP) isomers, enabling the identification of all four TCPP isomers (chromatogram below). *Features initially identified at level 2, later confirmed at level 1 with analytical standards.

#### Phthalates

Nine phthalate substances (i.e., diesters of phthalic acid) were annotated using FBMN/NAP, with some examples shown in Figure 1b. Seven of these were confirmed (Level 1), and two remained confidently annotated (Level 2b) due to lack of available standards; their close clustering in FBMN and similar retentions as confirmed phthalates increase confidence. Other features in the same cluster were curated as adducts or in-source fragments of phthalates—a pattern observed in other clusters too. The detection of multiple known and unknown phthalates in indoor passive air samples highlights their widespread presence and raises more concern about their cumulative health impacts.

#### Polyethylene Glycols

Eleven PEGs were tentatively identified using FBMN/NAP, which is particularly effective for grouping structural homologues such as PEGs, differing primarily in the length of their repeating ethylene oxide units. Two ethylene glycol oligomers—(HO–(CH_2_CH_2_O)_3_–H and HO–(CH_2_CH_2_O)_4_–H)—were confidently identified (Level 1), with nine other PEG oligomers confidently annotated (Level 2, examples in Figure 1c). PEGs are widely used in antifreeze, hydraulic fluids, and as polymer precursors and are a growing environmental concern.^88^ Glycol ethers are classified by the US EPA as hazardous air pollutants,^89^ while some are known for their potential toxicity to humans and wildlife.^90^ To our knowledge, they have not been reported in indoor or ambient air before, and further study on their indoor sources, fate, and impacts on humans is warranted.

#### Organophosphate Flame Retardants

Eight features annotated as OPFRs were confirmed (Level 1). A notable example was tris­(chloropropyl) phosphate (TCPP), a widely used OPFR, which was initially annotated via LC-HRMS and spectral-library matching. Further investigation of the GC-HRMS dataset by FBMN revealed additional peaks corresponding to four TCPP isomers (Figure 1e), which were not separated by LC due to coelution. This showcases how an integrated LC–GC approach, enhanced by FBMN, can broaden the chemical coverage and improve the resolution and sensitivity of airborne chemical profiling. The consistent detection of these compounds in indoor air underscores the need for continued monitoring of their sources and potential health risks. OPFRs, commonly used as flame retardants and plasticizers, have replaced brominated flame retardants in many consumer products.^91^ They are semivolatile, have been reported in air, airborne PM, water, and biological samples,^92,93^ and have been linked to neurotoxicity and endocrine disruption.^94,95^

### Passive Collection of Airborne Fine Particles on PDMS-Foam

In the first test of PDMS-foam for passive air sampling, we previously reported detection of complex mixtures of larger polar substances (e.g., oxygen, nitrogen, and sulfur-containing) that were more prominent compared to parallel sampling with solid PDMS-sheets, and we hypothesized these to originate from passive collection of airborne particulates.^19^ Previous studies have demonstrated that certain other types of passive air samplers can capture airborne fine particles,^96–98^ and moreover, many of the detected compounds, we report above have high octanol–air partition coefficients (log *K*_oa_ > 10^8^–10^9^, Table S15)—such as di-2-ethylhexyl phthalate, di-*n*-butyl phthalate, PCB-180, fluoranthene, pyrene and benzo­[*a*]­pyrene—and have been linked to PM in previous reports.^99–101^ To explore this hypothesis further here, indoor and outdoor PDMS-foam samples from Deployment 2 were analyzed using (i) chemical profiling of the aqueous extracts and (ii) microscopic imaging of the PDMS-foam surface.

#### Chemical Profiling of Aqueous Extracts

Unlike MeOH and hexane, used for the characterization of POCs and NPOCs, water is a poor extraction solvent for PDMS, and thus, any detectable WSOCs in the aqueous extracts of PDMS-foam were assumed to be particle-associated substances on the surface of the foam. Interestingly, across both indoor and outdoor samples here, WSOC extracts contained more nontarget molecular features than POC extracts in MeOH (Figure 2), specifically in positive ionization mode. In fact, in positive ionization mode, the WSOC extracts yielded two times more features outdoors and five times more indoors. This is in agreement with previous findings of PM2.5 collected using high-volume air samplers, which contained a predominance of WSOCs compared to POCs.^11,23^ Moreover, in both the indoor and outdoor PDMS-foam samples here, WSOC extracts consistently had features with molecular weights higher than those in the corresponding POC extracts. This partly reflects that WSOC extracts capture only particle-associated compounds, captured by the porous PDMS surface, and that airborne fine particles are known to contain a preponderance of relatively large, highly oxygenated water-soluble organic substances.^102^ In positive ionization mode, the average *m*/*z* of indoor WSOC features was 574 (average RT = 11.5 min), similar to outdoor WSOC features (average *m*/*z* 537, average RT = 12 min). For POC extracts, average *m*/*z* values were 440 for indoor and 443 for outdoor samples, both with longer average RT (∼15 min). A similar trend was observed in negative ionization mode, as WSOC features averaged *m*/*z* 748 and RT = 12 min indoors and *m*/*z* 612, RT = 12 min outdoors, while POC extracts contained substances with lower *m*/*z* (average *m*/*z* 389 indoors and average *m*/*z* 447 outdoors) and longer average RT of 15 and 14 min, respectively. Moreover, features in WSOC and POC extracts had very little overlap, with only 1.8% of features in common among the indoor samples, and 2.6% overlap among the outdoor samples (Figure 2), indicating that water and methanol extract a distinctive fraction of chemicals from the PDMS-foam samplers. Together, these data support the idea that PDMS-foam effectively captures a fraction of ambient PM containing a complex mixture of relatively large and polar water-soluble substances. Notably, both indoor and outdoor samples were deployed in housings designed to minimize the settling of large dust particles; thus, it is likely that a significant portion of the collected PM is fine (e.g., PM2.5).

**Figure 2:**
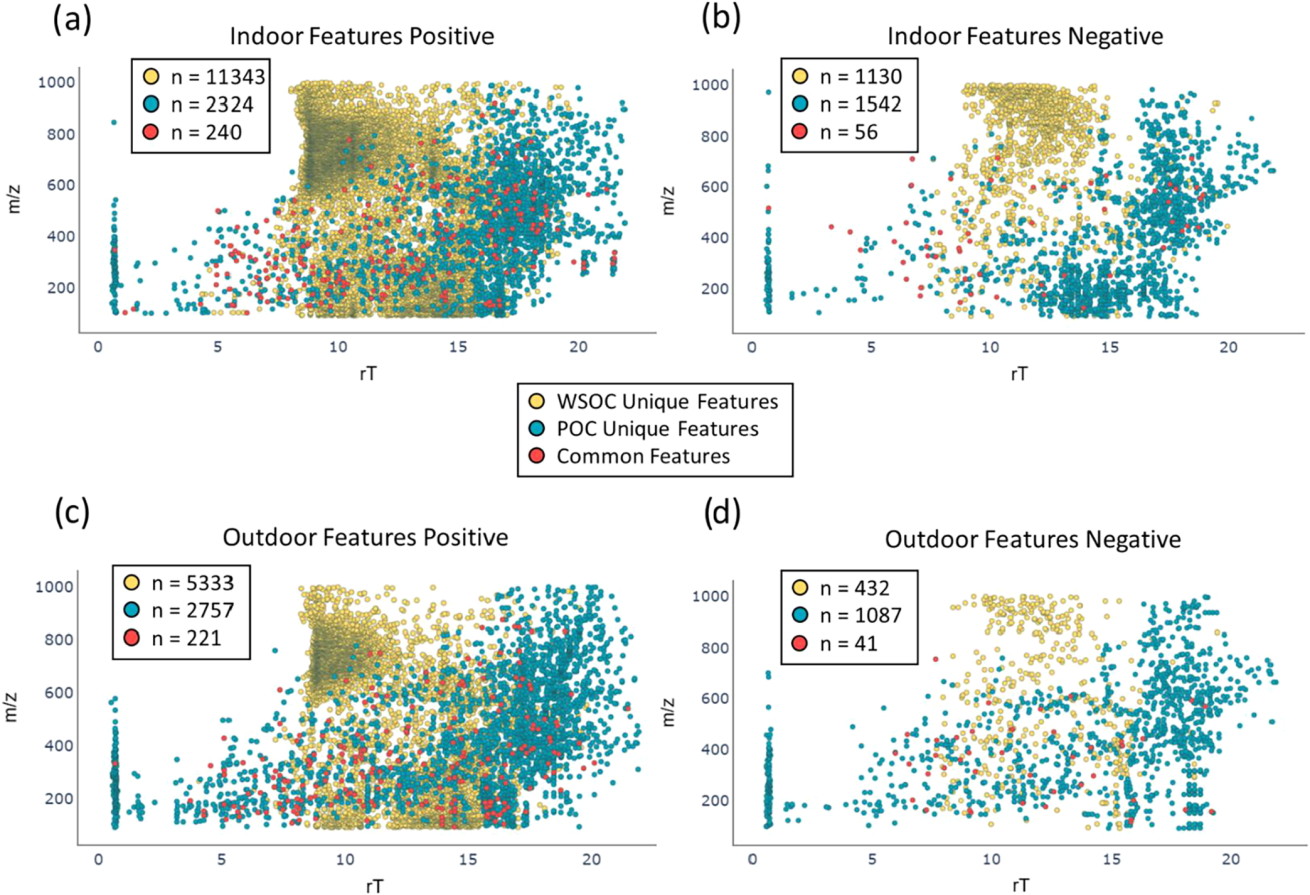
Indoor–outdoor feature profiles from PDMS-foam WSOC and POC extracts. Retention times and precursor ion *m*/*z* (MS1) of nontarget features in PDMS-foam from indoor and outdoor samples in Stockholm (Deployment 2) when extracted with water (WSOC) or methanol (POC). Data are plotted separately for (a) indoor features (ESI+), (b) indoor features (ESI−), (c) outdoor features (ESI+), and (d) outdoor features (ESI−). Unique features in WSOC are shown in yellow, for POC in teal, and features detected in both extracts are shown in red. WSOC: Water-soluble organic compounds. POC: Polar organic compounds.

#### Microscopy Analysis

The above chemical profiling suggested passive accumulation of fine particles by PDMS-foam samplers. A subset of replicate samples from Deployment 2 (indoor and outdoor) was thus used to employ microscopy to visually confirm particle deposition on sampler surfaces. Brightfield images of an outdoor sample reveal an abundance of fine particles compared to the field blank (Figure 3a). Imaging of the indoor samples, subsampled on days 1, 21, and 42, along with corresponding field blanks (Figure S26) demonstrated a progressive accumulation of PM. Because of the limited resolving power, bright-field microscopy was not adequate to accurately count or characterize these abundant dark fine particles. Nevertheless, with fluorescence microscopy, excitation under blue light (∼480 nm) allowed counting and characterization (size distribution and morphology) of a subset of fluorescing particles (Figure S27); acknowledging that this fluorescent fraction is not fully representative of all fine particles. When fluorescence emission was used in previous studies to characterize PM, it was linked to low-oxidation humic-like substances, PAH-like compounds, and protein-like materials.^103^

**Figure 3:**
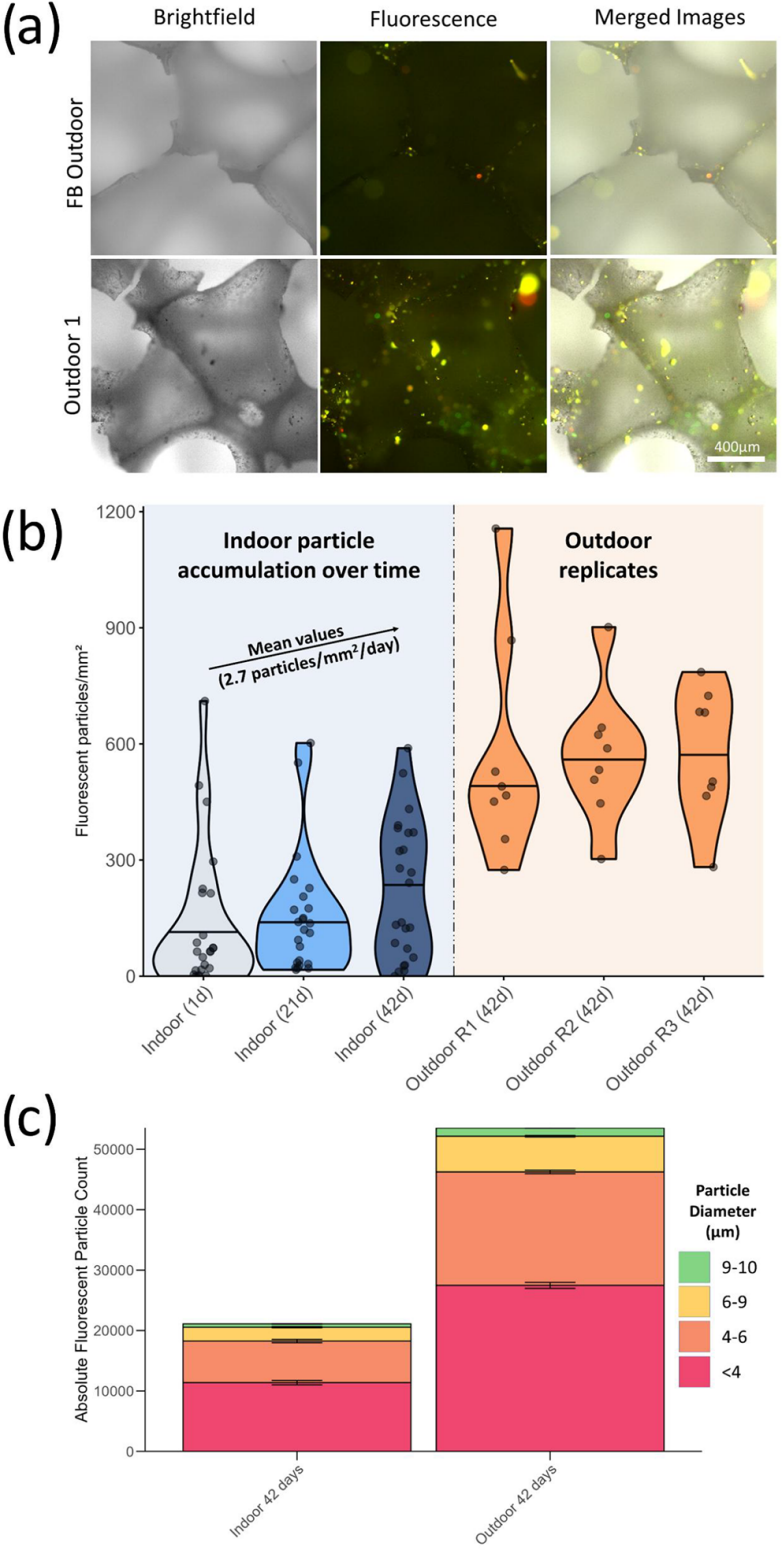
Microscopic visualization and quantification of particulate accumulation on PDMS-foam samplers. Microscopy was used to investigate the passive collection of fluorescent particulate matter on PDMS-foam samplers deployed indoors and outdoors. (a) Brightfield and fluorescence microscopy were used to compare deployed samples and field blanks. Brightfield images revealed abundant dark fine particles, many of which were too small or numerous for manual counting. Fluorescent particles, detectable by their emission signal, enabled automated quantification. Representative images are shown for one outdoor sample and its corresponding field blank. (b) Quantification of fluorescent particles per mm^2^ of PDMS-foam from indoor samples deployed for 1, 21, and 42 days shows a linear accumulation trend, alongside data from three outdoor samples deployed for 42 days. The shown data are blank-corrected using the mean field-blank values.(c) Fluorescent particle counts (*n* = 8) from indoor (*n* = 3) and outdoor (*n* = 3) samples after blank subtraction (*n* = 2), stratified by particle size (μm diameter).

Clear differences in fluorescent particle load, normalized to sampler area (particles/mm^2^), were observed between indoor and outdoor samples exposed for 42 days and their corresponding field blanks. This effect was most pronounced in the outdoor samples, where replicate samplers showed consistently high and comparable particle counts after field blank subtraction (520, 545, and 551 particles/mm^2^). Indoor samples deployed for 1, 21, and 42 days displayed lower overall particle accumulation but nonetheless demonstrated a time-dependent increase in fluorescent particles by linear regression (2.7 fluorescent particles/day/mm^2^) (Figure 3b). The sizes of fluorescent particles were similar between indoor and outdoor samplers, with the majority (>97%) measuring less than 9 μm in diameter. Size distribution of all fluorescent particles up to PM10 (Figure 3c) showed that particles with <4 μm diameter were the most dominant (54% indoor, 51% outdoor), followed by 4–6 μm (33% indoor, 35% outdoor) and 6–9 μm (11% in both). These trends are consistent with previous reports of passive air samples collected in housings designed to limit the deposition of larger particles.^7^ In terms of particle morphology (Figure S28), more than 85% of fluorescent particles exhibited high circularity with no significant shape differences between indoor and outdoor deployments. This suggests that particle deposition onto the PDMS-foam occurs without structural deformation and supports the notion that captured particles preserve their original morphology—likely indicative of anthropogenic sources, as documented in other studies.^104,105^

As a footnote to the microscopic analysis of particles on PDMS-foam, the area (*A*), perimeter (*P*), and circularity (*C*) of PDMS-foam alveoli were also measured microscopically (Figure S29). No significant differences (α = 0.05) were observed between samplers deployed outdoors and their corresponding field blanks. Average values for outdoor-deployed foams were *A* = 1.0 × 10^6^ mm^2^, *P* = 3.8 × 10^3^ mm, and *C* = 0.85, while field blanks showed similar measurements (*A* = 1.2 × 10^6^ mm^2^, *P* = 4.1 × 10^3^ mm, *C* = 0.83). No obvious discoloration was noticed, confirming the general stability of the commercial PDMS-foam over 6 weeks at the urban air quality station in Stockholm, Sweden (Figure S30). This stability may be an advantage relative to traditional passive samplers which are more susceptible to oxidation^12,14^ during deployment (Figure S31), but we acknowledge that a direct comparison of PUF and PDMS-foam has yet to be described. PDMS-foam is a silicone-based polymer that is thermally stable, enabling pretreatment by vacuum baking at 250 °C to lower the chemical background, and which exhibits reproducible partitioning behavior for airborne substances.^19^ A previous study has reported higher sampling rates for PDMS-sheets than for PUF for selected compounds,^96^ but further studies should be conducted to compare PDMS-foam and PUF. Because passive air sampling kinetics are generally air-side controlled,^106^ significant differences in sampling rates between PDMS-foam and PUF are not necessarily expected, and both samplers might yield similar quantitative results in short deployments. Accordingly, direct performance comparisons between PDMS-foam and PUF disks are beyond the scope of this study and should be considered preliminary. This discussion is intended to contextualize PDMS-foam as a promising sampling substrate, while recognizing that rigorous, quantitative comparisons will require future codeployment studies with established samplers such as PUF or SIP disks.

### Toxicity Assessment of Passive Air Samples and Virtual Effects-Directed Analysis

Only a few studies have previously explored the use of passive air sampler extracts for toxicological assessments, with most focusing on PUF.^107,108^ Here, primary human lung fibroblasts exposed for 48 h to 2% solutions of the PDMS-foam extracts from Deployment 3 in a megacity (Seoul, Korea) had significantly lower viabilities in every sample compared to paired field blanks (Figure 4a). PDMS-foam extracts decreased cell viability by 26–67%, with samples 2, 3, 4, and 7 exhibiting the greatest reductions (61, 58, 63, and 67%, respectively). Samples 1 and 5 had lower toxicity (33 and 26% reductions, respectively), presumably due to differences in the extractable chemicals. Most field blanks did not reduce cell viability relative to the negative control, although two (FB6 and FB7, Figure 4a) showed small, significant reductions of 4 and 10%, respectively.

**Figure 4:**
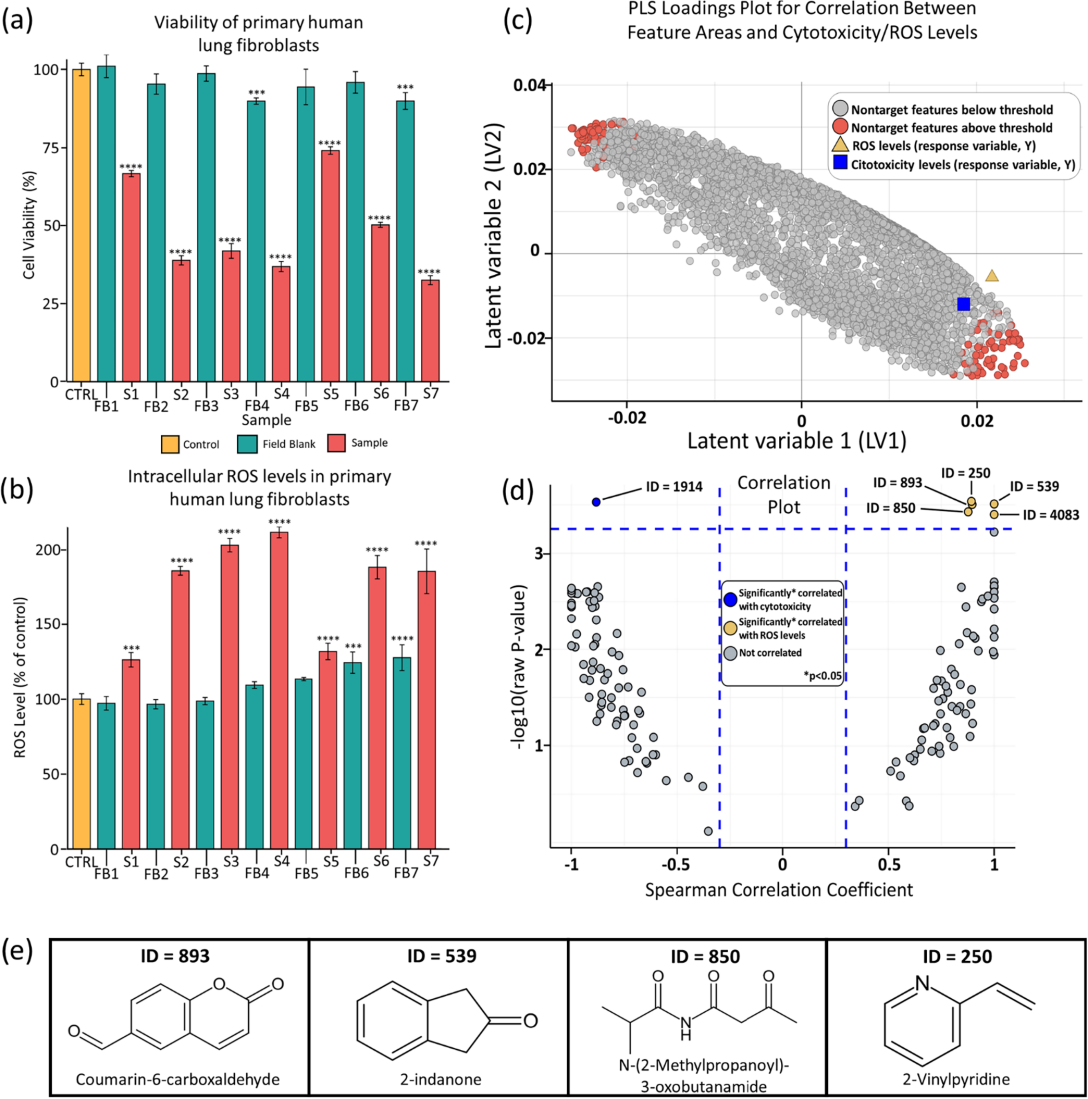
Integrating toxicity assays and nontarget analysis through virtual effects-directed analysis (vEDA). Toxicity assays combined with nontarget analysis enabled the prioritization and in-silico structural annotation of features associated with cellular toxicity. Primary human lung fibroblasts were exposed to 2% extracts from PDMS-foam disks (S), along with corresponding field blanks (FB), to assess (a) cell viability and (b) intracellular reactive oxygen species (ROS) levels. To identify features linked to toxicity, supervised multivariate modeling was performed using (c) partial least-squares (PLS; 3 components), with feature areas as predictors (*X*) and both cytotoxicity and ROS levels as joint responses (*Y*), given their similar response patterns. Features most strongly associated with toxicity were flagged using two criteria: (I) the top 1% of features by regression coefficient and (II) those with variable importance in projection (VIP) scores >1.5. (d) These prioritized features were further evaluated through individual Spearman correlation tests with Bonferroni correction (*p* < 0.05), and (e) structurally annotated in silico using SIRIUS software.^30^ Data are presented as mean ± SEM (*n* = 3 per treatment). Statistical analysis was conducted using one-way ANOVA followed by Dunnett’s test vs control. Significance: ns (not significant), *P < 0.05, **P < 0.01, ***P < 0.001, **P < 0.0001.

Intracellular ROS was also measured following the same exposure conditions (2% extracts, 48 h) (Figure 4b), and the positive control,^109^ TBHP, caused a dose-dependent increase in intracellular ROS levels (Figure S32). Consistent with cell viability, most field blank extracts did not significantly elevate ROS, except moderately in FB6 and FB7 (24 and 27%, respectively). In contrast, all PDMS-foam extracts significantly elevated ROS compared to that of paired field blanks. Among these, samples 2, 3, 4, 6, and 7 triggered increased ROS (86, 103, 111, 88, and 85%, respectively), whereas samples 1 and 5 led to relatively lower ROS increases (26 and 31%, respectively). Overall, a significant correlation (*p* = 0.015, *r* ^2^ = 0.73) was observed between cytotoxicity and ROS (Table S16), suggesting that intracellular oxidative stress may be a mechanism of cytotoxicity in these primary human cells exposed to complex mixtures of airborne POCs.

As a proof-of-principle, we demonstrate how toxicological data can be integrated with nontarget chemical analysis through a virtual effects-directed analysis (vEDA) approach. The vEDA concept, originally introduced for water samples,^110,111^ uses multivariate statistical modeling—typically PLS-based pattern-recognition methods—to reduce the complexity of large mixture datasets. It extends the principles of classical EDA into a computational framework with many samples, whereby correlations between detected chemical features and measured biological responses (e.g., cytotoxicity and oxidative stress) are examined to flag the most relevant toxicological drivers, thereby negating the need for fractionation or bioassay-guided chemical isolation in the initial stages. A wide range of organic compounds were quantified in the POC extracts of all seven samples (Table S17), including aromatic amines (1,3-diphenylguanidine, 1*H*-benzotriazole), phenolics (2,5-dichlorophenol, 4-nitrophenol), pesticides and industrial chemicals (2,4-dinitroaniline, atrazine, DEET, bioallethrin, chlorothalonil-4-hydroxy), organophosphates (tri-*n*-butyl phosphate, triphenyl phosphate), and PFAS (perfluorobutanoate, perfluorononanoate). Although several of these, including 4-nitrophenol, DNOC,^112,113^ DEET,^114,115^ triphenyl phosphate,^116,117^ and perfluorobutanoate,^118,119^ have been previously associated with ROS generation, none showed a significant positive correlation with cytotoxicity or ROS here based on Spearman correlations (Table S18). However, three target compounds—palmitamide (*p* = 0.008), 4-toluenesulfonamide (*p* = 0.011), and methyl paraben (*p* = 0.049)—were positively correlated with ROS. Methyl paraben has previously been associated with increased ROS in human spermatozoa.^120,121^

Considering all detectable nontarget features in the corresponding POC extracts, a total of 7692 blank-subtracted features (areas normalized by internal standards) were included in the supervised PLS regression and modeled using cytotoxicity and ROS levels as *Y* response variables (Figure 4c). Important variables in the projection (VIP) were then prioritized for follow-up structural in silico characterization.

A total of 125 features (1.6% of the dataset; Table S19) corresponding to VIP scores >1.5 model (Figure S33a) or the top 1% of positive or negative correlations with either cytotoxicity (Figure S33b) or ROS levels (Figure S33c) in the PLS were thus further selected for subsequent Spearman′s correlation tests with Bonferroni multiple comparison correction. Finally, six features, all of which have unknown molecular structures, were highlighted as significantly correlated with toxicity (corrected *p*-value <0.05) (Figure 4d). Of these, one feature showed a negative correlation with cytotoxicity (i.e., protective effect; *p*-value = 0.03), and five were positively correlated with cellular ROS (*p*-value <0.027).

In-silico formula and structural prediction in SIRIUS^122^ allowed to provide putative annotations for four of these chemicals, namely coumarin-6-carboxaldehyde, 2-indanone, 2-methylpropanoyl-3-oxobutanamide, and 2-vinylpyridine (Figure 4e). Notably, these four compounds are listed in the EPA DSSTox database,^123^ a resource for predictive toxicology, suggesting that they have been previously associated with potential toxicity or adverse biological effects. Both 2-indanone and 2-vinylpyridine are also included in the “Blood Exposome” database,^124^ indicating prior detection in human biomonitoring studies. Notably, while 2-indanone is a xenobiotic transformation product—often formed during the oxidation of polycyclic musks or other fragrance compounds and commonly found in fragranced consumer products—it lacks sufficient toxicological data.^125^ 2-Vinylpyridine is an azaarene, i.e., sharing structural similarity with PAHs but with a nitrogen inside the aromatic ring. Azaarenes are known to exhibit toxicity comparable to or exceeding PAHs.^126,127^

Although this vEDA analysis highlights the potential toxic-drivers among thousands of airborne substances, the results are not conclusive without confirmation tests with these chemicals in cells, and we acknowledge only seven samples from a single location, in addition to the uncertainties in some in-silico annotations. Nonetheless, we suggest that integrating the nontarget analysis of passive PDMS-foam air samples with bioassays in human-relevant cell systems offers a scalable and practical path forward. Unlike air–liquid interface systems, which are limited to examining air in specific individual settings, this passive sampling approach can enable broader deployment, including in indoor environments. This is particularly relevant in megacities like Seoul, where air pollution has been linked to elevated morbidity and mortality,^128^ underscoring the need for accessible tools to monitor and assess complex chemical exposures at scale in comparison to background control sites. This approach combining nontarget analysis and toxicity testing of air samples offers a valuable opportunity to improve chemical management strategies and better evaluate real-world exposures to complex chemical mixtures.^129^

### Applicability and Future Directions

This study establishes PDMS-foam passive samplers as a powerful multimodal tool for the comprehensive characterization of airborne chemicals and particulate matter in both indoor and outdoor environments. We demonstrate for the first time how PDMS-foam samplers, combined with multiclass quantification and nontarget analyses, bridge a critical gap in current air monitoring approaches, offering a more comprehensive perspective on air quality and exposure assessment. Data on this capability remain scarce, and the contribution of captured PM is often overlooked in exposure assessments.^130^ This dual capability enhances the potential of PDMS-foams for large-scale monitoring campaigns, particularly in exposomics and environmental health research, and highlights the value of untargeted approaches for understanding the airborne chemical exposome, enabling individual-level exposure assessment, more comprehensive mixture risk evaluations, and future data-driven public health interventions.

While this work provides a foundation for PDMS-foam-based passive sampling, further analyte stability and calibration studies are needed to accurately estimate airborne concentrations—crucial steps toward wider adoption for monitoring and epidemiological applications. One possible strategy is to calibrate PDMS-foam through a codeployment alongside PUF and/or SIP disks of matching dimensions, as the sampling rates and sampling behaviors of the latter materials are already established.^131–133^ The quantitative results presented here (i.e., ng analyte/mL extract) therefore need to be interpreted with care until future data allow these to be expressed as airborne concentrations (i.e., ng/m^3^). Similarly, the semiquantitative results presented here for GC-HRMS annotated compounds should be interpreted with caution, as they are inherently less accurate than those obtained for target analytes. Combining PDMS-foam sampling with nontarget screening and bioassays can nevertheless proceed immediately to uncover correlations between known and unknown toxicants in air and their potential toxic effects, but improved quantitation in the future can help to strengthen these links and to drive public health interventions.

## Supplementary Material





## Notes

Supporting Information Sections (Sections S1–S13) and Supporting Information Figures (Figures S1–S33) describing sampling materials, analytical methods (LC- and GC-HRMS), data processing, annotation strategies, toxicological assays, and statistical analyses are provided in a single PDF Supporting Information file (PDF)
Supporting Information Tables (Tables S1–S20) containing detailed results and metadata are provided as an XLSX file (XLSX)
