# Supplementary material for: Silicone-Foam Passive Air Samplers for Combined Target and Nontarget Chemical Profiling and Toxicity Assessment of Airborne Exposomes
Source: Environ Sci Technol. 2026 Feb 10;60(7):5628–44. doi: 10.1021/acs.est.5c16613 (PMC12947678; doi:10.1021/acs.est.5c16613)
Supplement: Supplementary file 1 [file es5c16613_si_001.pdf]

Silicone-Foam Passive Air Samplers for Combined Target and Nontarget Chemical Profiling and Toxicity Assessment of Airborne Exposomes

Adrià Sunyer-Caldú[1], Hongyu Xie[1], Bénilde Bonnefille[1,2], Foteini Raptopoulou[1], Edouard Pesquet[3], May Britt Rian[1], Daniel Schlesinger[4], Michael Norman[4], Young June Jeon[5], Boram Kim[5], Seung-Bok Lee[6], Ji Eun Lee[5], Jean Froment[1,7], Stefano Papazian[1,2], and Jonathan W. Martin[1,2*]

[1] Department of Environmental Science, Science for Life Laboratory, Stockholm University, Stockholm 10691, Sweden
[2] National Facility for Exposomics, Metabolomics and Exposomics Platform, Science for Life Laboratory, Stockholm University, Solna 171 65, Sweden
[3] Arrhenius Laboratories, Department of Ecology, Environment and Plant Sciences (DEEP), Science for Life Laboratory, Stockholm University, Stockholm 10691, Sweden
[4] SLB-Analys, Environment and Health Administration, City of Stockholm, 104 20 Stockholm, Sweden
[5] Chemical & Biological Integrative Research Center, Biomedical Research Division, Korea Institute of Science and Technology, Seoul 02792, Republic of Korea
[6] Center for Sustainable Environment Research, Climate and Environmental Research Institute, Korea Institute of Science and Technology, Seoul 02792, Republic of Korea
[7] Department of Environmental Chemistry and Health Effects, NILU, Kjeller, NO-2027, Norway

*Corresponding author: jon.martin@aces.su.se

This PDF contains:

Supplementary Sections (Section S1-S13)

Supplementary Figures (Figure S1-S33)

Supplementary Sections

Section S1. Commercial information, cleaning optimization, and PDMS background testing.

The commercially obtained PDMS-foam sheets (1.5 cm thickness, Bisco MF1-55 Bun Silicone Foam, Rogers Corporation, USA) were water-jet cut into disks with final dimensions of 1.5 cm thickness × 14 cm Ø (surface area 373 cm^2), matching the size of passive samplers used in the Global Atmospheric Passive Sampling (GAPS) network[1]. For selected applications, these larger disks were further cut with a cork borer (2.5 cm Ø) to produce smaller disks (1.5 cm thickness × 2.5 cm Ø, surface area 21.6 cm^2). Cleaning involved a two-cycle pressurized liquid extraction (PLE) with MeOH/ethyl acetate (50:50) followed by hexane/ethyl acetate (50:50) at 125°C, and subsequent vacuum baking at 250°C for 1 h under nitrogen (>99.99% purity). These parameters were optimized to minimize residual background and maintain PDMS integrity (Figure S3).

Subsequent GC-HRMS analysis of pre-cleaned commercial PDMS-foam revealed an elevated signal of PDMS oligomers, which were monitored through characteristic fragments, including m/z 73.0468 (C_3H_9Si), 147.0656 ($C_5H_{15}OSi_2$), 221.0844 ($C_7H_{21}O_2Si_3$), 295.1033 ($C_9H_{27}O_3Si_4$), and 369.1220 ($C_{11}H_{33}O_4Si_5$)(**Figure S2**). The PDMS-oligomer background was of concern because it could lead to matrix interference and contamination of the GC ion source resulting in signal suppression of analytes. Thus, we further optimized the pre-cleaning procedure to minimize extraction of PDMS-oligomers, which improved method sensitivity and allowed to prolong analytical sequences. To refine PDMS cleaning, we assessed the influence of multiple parameters on siloxane background levels in extracts. These included disk dimensions, PDMS foam quantity, number of cleaning cycles, vacuum oven temperature, extraction solvent type, and extraction temperature. Lower siloxane content in extracts allowed longer analytical sequences and reduced signal decay in GC-HRMS. The PDMS background signal was monitored by analyzing extracts using GC-HRMS and tracking the following masses: 73.0468 m/z (C3H9Si), 147.0656 m/z (C5H15OSi2), 221.0844 m/z (C7H21O2Si3), and 369.1220 m/z (C11H33O4Si5) (Figure S2).

No significant variation in signal intensity was observed between smaller and larger PDMS disks, indicating consistent PDMS background levels across samples. To ensure consistency between different injection batches, a solvent blank and an extract from previously synthesized foam were always injected alongside the test samples for comparison. Description of the tested parameters:

1) *Cleaning protocol*

- **ASE cleaning cycles**: Standard cleaning cycles in the ASE consisted on using (I) MeOH/ethyl acetate (50:50) at 125°C and (II) hexane/ethyl acetate at 125°C. As an alternative, four solvent cleaning cycles were tested (x1 of MeOH/ethyl acetate, x3 hexane/ethyl acetate).
- **Time in the vacuum oven:** The standard baking time for PDMS foam disks in the vacuum oven was one hour. As alternatives, both skipping the oven step and extending the baking time (ranging from 2 to 22 hours) were tested. However, after 10 hours or more, the PDMS foam disks became dried, brittle, and easily breakable, indicating a change in texture and composition.
- **Temperature in the vacuum oven:** The standard baking temperature for PDMS foam disks in the vacuum oven was 250°C. Higher temperatures (up to 290°C – upper limit of the oven) were tested. Like for extended times, after baking at higher temperatures, the PDMS foam disks became dried, brittle, and easily breakable, indicating a change in texture and composition.

2) *Chemical extraction*
   - **Extraction temperature:** Standard temperature for extraction was 125°C in the ASE. Lowest temperature allowed by the instrument was tested (40°C), which resulted to decrease PDMS content in the extract without affecting analytes extraction.

   - **Extraction solvent:** Standard solvent for chemical extraction was hexane. A 50:50 mixture of hexane and acetone was tested, but increased the PDMS content significantly.

   - **Final volume extract:** Standard final extract volume before injection was 0.5 mL. However, after some tests it was observed that increasing the volume (1 mL) was decreasing considerably the measured siloxane levels—often exceeding the expected reduction based on dilution.

3) *Amount of cleaned/extracted PDMS*
   - **Disk size for cleaning:** The standard disk size used for cleaning was 2.5 cm Ø. For larger disks (14 cm Ø), poking a hole of 2.5 cm Ø on them, and performing the same extraction and analysis conditions, siloxane content in the extract from the big disk was always significantly higher. This highlights that the cleaning of big disks, was not as efficient as with the small ones, and probably additional ASE cycles are needed to lower siloxane content if big disks are going to be deployed.

- **Disk size for extraction:** The standard disk size used for extraction was 2.5 cm Ø. As expected, a larger amount of PDMS material extracted resulted in higher siloxane content in the extract. The effect of using smaller 1.25 cm Ø disks for extraction was also evaluated, but it did not significantly impact the siloxane content in the extracts.

4) *Storage time after cleaning*

   - Among the various tests, it was observed that freshly cleaned PDMS foams and PDMS foams cleaned under identical conditions but stored for a period of time exhibited different siloxane levels in the extract. When comparing storage times (none vs. 3 months), PDMS foams stored for 3 months consistently produced higher siloxane content in the extract. It suggests that PDMS may undergo slow chemical changes, such as the reformation of siloxane bonds.

The optimized pre-cleaning protocol allowed to minimize the background of PDMS oligomers in the POC extracts, resulting in enhanced analytical sensitivity and higher signal stability through extended GC-HRMS sequences (**Figure S1**). This issue was not present for LC-HRMS. Various conditions were evaluated (**Table S1**), revealing that vacuum oven temperature, baking time, extraction solvent, extraction temperature, and disk size were all key factors (**Figure S3**). Notably, additional solvent extraction cycles yielded minimal reduction of PDMS oligomers. The optimal protocol consisted of two PLE solvent extractions—MeOH/ethyl acetate (50:50), then hexane/ethyl acetate (50:50)— at 125°C, followed by vacuum baking at 250°C for 1 hr and cooling under a nitrogen atmosphere (>99.99% purity).

Section S2. Chemical extraction for airborne exposomics.

For the POCs extraction, 200 µL of a mixture of 35 isotopically labelled internal standards (final concentration 2 ng/mL) were spiked onto the surface of the small PDMS foams (1.5 cm thickness × 2.5 cm Ø, surface = 21.6 cm²). After solvent evaporation, the foams were placed in 22 mL stainless steel cells of a pressurized liquid extractor (PLE) (ASE 350 Thermo Scientific). In the PLE, the samples underwent a solvent extraction with a static cycle of 20 mL of MeOH at 125°C, which were collected in clean 60 mL collection ASE vials. After extraction, the samples were reduced to 0.5 mL under nitrogen flow (TurboVap, Biotage; 35 °C, 1.2 L/min) and transferred to amber glass vials (1.5 mL, Thermo Scientific). Water (Optima LC/MS Grade, Fisher Chemical) was then added to the final extract to reach a 50:50 methanol-water ratio for chromatographic analysis. Finally, 200 µL of diuron-d6 solution (final concentration 2 ng/mL) was added to correct for extract volume variations and monitor instrumental performance. The extraction conditions were optimized using pre-deployed disks from Deployment 1 to ensure high recoveries, broad chemical coverage, and minimal PDMS-oligomer background.

For NPOCs extraction, 200 µL of a different mixture of 35 isotopically labelled internal standards (final concentration 2 ng/mL) were spiked onto the surface of the small PDMS foams (1.5 cm thickness × 2.5 cm Ø, surface = 21.6 cm²). After solvent evaporation, the PDMS foams were extracted using a similar process to the POC method, but with 20 mL of iso-hexane (Merck, Germany) at 40°C. After extraction, the samples were reduced to 0.8 mL under nitrogen flow (TurboVap, Biotage; 35 °C, 1.2 L/min) and transferred to amber glass vials. The final extract was spiked with 200 µL of n-dodecane-d26 solution (final concentration 2 ng/mL) for volume correction and to monitor instrumental performance. All extracts were subsequently prepared for LC- or GC-HRMS analysis depending on compound polarity.

For WSOCs extraction, a quarter of the large PDMS foam disks (1.5 cm thickness × 14 cm Ø, surface = 373 cm²) were extracted by soaking them in 5 mL of HPLC water (VWR, Sweden) while shaking at 230 rpm (Mini Shaker, VWR) for 1 hour. After agitation, the samples were filtered using 0.2 µm regenerated cellulose syringe filters (Sartorius Minisart RC 15 mm). These filters were precleaned with 5 mL methanol and dried under a fume hood.

Section S3. POC and NPOC Method Validation.

POC and NPOC Method Validation. POC and NPOC methods were optimized and validated using indoor passive samples from Deployment 1, and using optimized workflows designed to integrate both target and nontarget analysis in a single injection. Absolute recoveries were determined by comparing analyte response in replicate samples (n = 5) spiked with native standards before and after extraction. For POCs, 118 native standards were used, and 104 for NPOCs, each tested at two concentration levels (1–2 ng/mL, 10–20 ng/mL in final extracts). Absolute recoveries (no IS corrected) were calculated based on the peak areas, adjusted for any background analytes in non-spiked samples. Precision was determined using the relative standard deviation (RSD) between replicates. Matrix-matched calibration curves were generated by pooling sequentially extracted field blanks, which were then spiked with IS and native standards. A 10-point calibration curve was built for POCs (0.005–100 ng/mL; for LC) and for NPOCs (0.001–200 ng/mL; for GC) to assess linearity and matrix effects by comparing slopes of matrix-matched curves with those of solvent calibration curves. Method limits of quantification (MLOQs) were defined as the lowest concentration detected in the matrix-matched calibration curves with an RSD under 20%, or when blank signal was present by the mean concentration in field blanks plus three standard deviations. A summary of method applications across deployments is in Figure S7.

Section S4. Quality assurance, quality control.

All samples were prepared in a positive pressure clean laboratory with high efficiency particulate filtration. Procedural blanks were prepared for all experiments (optimization, validation, and analysis of individual samples) and treated in the same manner as the experimental samples. All glass materials were solvent cleaned and furnaced before use. Aluminum foil used to protect the foams was furnaced before use. Targeted analytes were in general absent in field blanks. For quantification, field blank subtraction was always performed to samples before concentration reporting. Instrumental blanks consisting of clean solvent were also run multiple times in the injection sequence to track carryover. Matrix matched calibration curves for quantification were run 3 times during the injection sequence (beginning, middle, end).

Section S5. LC- and GC-HRMS.

LC-HRMS

POCs were analyzed using an Acquity BEH C18 column (130 Å, 1.7 µm, 2.1 × 100 mm)(Waters) at 40°C with a Vanguard pre-column of the same material. A trap column (C18 Waters BEH, 130 Å, 1.7 µm, 3 × 30 mm)(Waters) was used before the injector to separate instrumental background analytes from sample analytes. The flow rate was 0.4 mL/min, and HRMS acquisition was carried out in both positive and negative ESI modes. The mobile phases consisted of water with 1 mM ammonium fluoride (A) and MeOH (B). Injection volumes were 10 µL. Full scan (MS1) was conducted at nominal resolution of 120,000 FWHM (90−1000 m/z, ~3 Hz) and with parallel data-independent acquisition (DIA) MS/MS (MS2) at a nominal resolution of 30,000 FWHM; five DIA precursor windows were used covering the MS1 precursor range of 50–1040 m/z, ~12 Hz. Stepped normalized collision energy (stepped NCE) was used for fragmentation (20-70 eV).

GC-HRMS

NPOC extracts were separated on a DB-5MS column (Agilent) using a TRACE 1300 Series GC system (Thermo Fisher Scientific). The carrier gas was helium at 1 mL/min, and 2 µL of extract was injected using a PTV splitless injector. The temperature program began at 70 °C, increased to 300 °C at 10 °C/min, held for 4 min, then increased to 320 °C at 10°C/min with an 11 min hold, for a total run time of 41 min. Kovats Retention Index (RI) of detected analytes was calculated using n-alkane standards (C7−40 mixture) (Dr. Ehrenstorfer). The HRMS acquisition was performed on a Q Exactive Orbitrap (Thermo Fisher Scientific) operating with electron ionization (EI, 70 eV) in full scan mode (40−750 m/z) at a nominal resolution of 60,000 FWHM. A PTV splitless injector was used with a Siltek Metal Liner (2 mm ID × 120 mm). The carrier gas was helium at 1 mL/min, and 2 µL of extract was injected. The ion source and transfer line temperature were set to 300°C.

Section S6. Feature signal normalization.

Internal standard (IS) areas were integrated using the MS-Dial inclusion list feature and subsequently used for principal component analysis (PCA) in SIMCA® 17 software, with IS areas as variables and samples as observations. Prior to modeling, the IS areas were normalized using Univariate Variance Normalization (UVN) to ensure equal contribution of each variable. The model was then autofitted, and principal component scores for each sample were utilized for sample normalization. After normalization, feature areas were blank-subtracted by subtracting the averaged normalized values of the corresponding features from field blanks.

Section S7. Data processing.

Data Processing Parameters NPOCs workflow (MS-DIAL)

Project data type GC-MS for EI data ("Metabolomics") with "profile" mode, and accurate mass. Number of parallel processing threads: 8. Peak detection parameters, smoothing method: "Linear Weighted Moving Average", with smoothing level 3. Minimum peak width: 20 scans. Minimum peak height: 10000, mass slice width: 0.025 Da. Deconvolution parameters, sigma window value: 0.5, cut off: 3. Identification setting, GNPS + in-house library (August 2024), Retention type: RI ("Alkanes"), RI index file (C8-C40 alkane series). EI similarity library tolerance: 60, identification score cut-off: 60. Use retention information for scoring: True (40). Alignment parameters setting, retention index tolerance: 10, EI similarity tolerance: 70, retention time factor: 0.5, EI similarity factor: 0.5. Identification after alignment: True. Gap filling by compulsion: True. Base peak m/z selected as the representative quant mass: True. Filtering setting, peak count filter: 0, remove feature based on peak height fold-change: True, with sample max / blank average: 5.

Data Processing Parameters POCs workflow (MS-DIAL, ESI+ and ESI-)

Project data type LC-MS, for DIA data ("Metabolomics") with MS1 and MS2 both "profile" modes. Centroid parameters: MS1 tolerance 0.001 Da and MS2 tolerance 0.005 Da. Isotope recognition Maximum charged number: 2. Consider Cl and Br elements: True. Number of parallel processing threads: 8. Peak detection parameters, smoothing method: "Linear Weighted Moving Average", with smoothing level 3. Minimum peak width: 9 scans. Minimum peak height: 90000. Peak spotting parameters, mass slice width: 0.05 Da. Deconvolution parameters, sigma window value: 1, cut off: 0. Exclude after precursor: True. Keep isotope until: 5 Da. Keep original precursor isotopes: True. MS/MS identification setting, Library MS-Dial + In-house (August 2024), accurate mass tolerance (MS1) 0.003 Da, (MS2) 0.005. Using retention time for scoring/filtering: False. Identification score cut off: 80. Alignment parameters setting, retention time tolerance: 0.2 min, MS1 tolerance: 0.002, retention time factor: 0.5, MS1 factor: 0.5, peak count filter: 0%, N% detected in at least one group: 0%. Remove feature based on peak height fold-change: True, with sample max / blank average: 10. Gap filling by compulsion: True.

Section S8. In-silico tools for annotation of molecular formulas and structures.

SIRIUS software (v.6.1.1) was used for in-silico annotations. Spectral information of prioritized features was exported to SIRIUS as .txt files. Then, features were computed with these parameters: Instrument: Orbitrap, MS2 mass accuracy (ppm) 3, Possible adducts: [M+H]+, [M+Na]+, [M]+, [M+H2O]+, Molecular formula generation: De novo + bottom up, Predict properties: CSI:FinderID: True, Score threshold: false, Search DBs: True, all available databases searched, MSNovelist: True.

Section S9. Molecular annotation parameters.

Feature-based molecular networks (FBMN) were generated in GNPS (https://gnps.ucsd.edu/) release version 28.2, using LC-HRMS data pre-processed with MS-DIAL. Parameters were: precursor MS1 and MS2 fragment tolerance: 0.02 Da, minimum spectra similarity cosines of ≥ 0.65, with a minimum of 2 matched spectral peaks. Network graphs were visualized using Cytoscape v.3.8.2. Molecular networking jobs are publicly accessible and visualizable directly in GNPS web-browser, at the following links https://gnps.ucsd.edu/ProteoSAFe/index.jsp?task=96a39776b047463aab240104c41ffe52 (ESI+) and https://gnps.ucsd.edu/ProteoSAFe/status.jsp?task=898af885586b4c759427a7655e0aaf83 (ESI-). For in silico structural prediction, the GNPS workflow NAP ("Network Annotation Propagation") was used in combination with the structural database PubChemLite for Exposomics v.1.33.0 (accessed: 26

Apr 2024; https://zenodo.org/records/11070224) consisting of 371,663 molecular structures compiled from PubChem. Parameter settings for NAP were set as: 10 n-first candidates, 5 ppm accuracy, and cosine score ≥ 0.65, with Consensus + Fusion ranking algorithm. Results from the NAP workflow are publicly accessible at the University of California San Diego - Center for Computational Mass Spectrometry (CCMS) server, under: https://proteomics2.ucsd.edu/ProteoSAFe/status.jsp?task=8d1c2a2744414470b3c0255ab88c24fb in ESI+ and https://proteomics2.ucsd.edu/ProteoSAFe/status.jsp?task=088a44adc3084bd0a120dfefa9996a42 in ESI-. Molecular networks were generated in GNPS release version 30, using GC-HRMS data pre-processed with MS-DIAL. Parameters were: Fragment Ion Mass Tolerance: 0.4 Da, minimum of 5 matched spectral peaks, score threshold: 0.8, min pars Cos: 0.5.

Section S10. Semi-quantification of nontarget annotations.

Semi-quantification of confidently annotated features (reported in **Tables S13 and S14**) was performed by normalizing each annotation in both samples and field blanks to the closest eluting internal standard. Following blank subtraction, normalized peak areas were converted to extract concentrations (ng/mL) based on the known concentration (2 ng/mL) of the internal standard spiked in the samples. To obtain sampler surface–normalized values (ng/cm^2), extract concentrations were converted to mass per sampler and divided by the corresponding sampler surface area. All results from this procedure are reported and discussed as semi-quantitative estimates.

Section S11. Microscopy data processing.

A stereomicroscope (Zeiss Stemi 2000-C, Carl Zeiss, Germany) was first used for gross evaluation of PDMS-foam surfaces. Detailed imaging was performed with an inverted epifluorescence microscope (Zeiss Axiovert 200M) equipped with a long-working distance 5× objective (NA 0.15), a high-resolution color camera (Zeiss AxioCam 512), and Zeiss Colibri multicolor LED illumination. Images were acquired in both autofluorescence and brightfield modes.

Semi-automated image processing was carried out in ImageJ. Raw 16-bit RGB stacks were converted to 8-bit RGB and merged into grayscale images. Background-to-signal separation was performed by thresholding >95% of the image, followed by binarization. Automated particle analysis was used to outline each particle's region of interest and quantify particle area, density, and shape descriptors, including circularity (similarity to a perfect circle) and convexity (similarity to a circular convex hull), following previously established protocols for biological samples[2].

Obtained ROIs from detected particles were placed onto the corresponding 8-bit RGB image stack to determine changes in each of the RGB channel to determine autofluorescence emission color. Particle size were selected for area above 0.68 µm2 to stay within the instrument detection limit and ensure that particles detected were confirmed by multiple pixels and their relative color emission was determined independently of their intensity using the red to green 8-bit ratio with >1,5 for red, <0.75 for green and in-between for yellow. Although no confocal section was used thus leading to an overestimation of particle size due to light diffraction across multifocal images, conditions were kept similar between films to maintain effect of optical artefact similar between treatment.

Section S12. Cytotoxicity assessment and ROS levels measurement.

One-twelfth of each PDMS-foam disk was cut in half, placed into 50 mL Falcon tubes, and covered with ~31 mL of MeOH. The samples were sonicated for 30 min and centrifuged at 1,800 g for 10 min. The supernatant was divided into three 15 mL tubes, evaporated under nitrogen flow to 3 mL in each, recombined, and further reduced to 1 mL. To this, 4 mL of HPLC water was added, followed by filtration through regenerated cellulose syringe filters to remove fine particles and PDMS residues, and careful evaporation to 20 µL. After centrifugation at 16,000 g for 40 min at 4 °C, the supernatant was transferred to 0.6 mL vials for cellular assays.

To evaluate cytotoxicity, primary human lung fibroblasts (Lonza, Basel, Switzerland) were seeded in 96-well plates at 4,000 cells/well and cultured to ~50% confluence. Cells were exposed to 100 µL of PDMS-foam extracts (2% of the original extract concentration) or field blanks for 48 h. Control cells received fresh culture media. Cell viability was assessed using the CCK-8 assay (Dojindo Laboratories, Kumamoto, Japan) by adding 10 µL of CCK-8 solution per well, incubating at 37 °C for 2 h, and measuring optical density (OD) at 450 nm. Cell viability (%) and cytotoxicity (%) were calculated as:

$$(1)\ \text{Cell viability (\%)} = \frac{(\text{OD value of treatment} - \text{OD value of blank control})}{(\text{OD value of control} - \text{OD value of blank control})} \times 100$$

Intracellular ROS levels were measured using the Cellular ROS Assay Kit (Abcam, Cambridge, UK). Cells were seeded in black 96-well microplates at 4,000 cells/well and exposed to 100 µL of 2% extracts for 48 h at 37 °C. Control cells were maintained in fresh media. Positive controls were treated with tert-butyl hydroperoxide (TBHP, 137.5, 275, and 550 µM) 4 h before fluorescence measurement. After exposure, 100 µL of 40 µM H_2DCFDA solution was added to all wells without removing the media, incubated at 37 °C in the dark for 45 min, and fluorescence was measured at 485/20 nm excitation and 528/20 nm emission. ROS levels (%) were calculated as:

$$(2)\ \text{ROS level (\% of control)} = \frac{(\text{Fluorescence of treated cells})}{(\text{Fluorescence of control cells})} \times 100$$

Section S13. Statistical analysis.

Principal component analysis (PCA) for normalization of nontarget feature response, based on IS profile response, was conducted in SIMCA® 17 (Sartorius) as described previously[3]. Microscopy data were analyzed using one-way ANOVA followed by a post-hoc Tukey-Kramer test (α = 0.05). Toxicological data were analyzed with one-way ANOVA followed by Dunnett’s test to compare treatment groups with the control using GraphPad Prism (v10.4.1). For virtual effects-directed analysis, initial assessment of toxicity correlation with individual feature areas (non-detect values were replaced with 10% of the lowest detected peak area) was performed using partial least squares (PLS) in SIMCA® 17 to identify features (X; explanatory variables) most strongly associated with cell viability and ROS (Y; response variables). Mean-centring and unit variance (UV) scaling were applied to feature areas. Then, the modelling software automatically tested several PLS models (with different numbers of variables) using 7-fold cross-validation and chose the optimal number of components (three in this case) based on predictive performance. We employed a partial least squares discriminant analysis regression model because it inherently down-weights features detected at low frequency in the samples, allowing to discriminate strong correlations caused by low detection frequencies. Model significance was supported by CV-ANOVA (p = 0.05). Flagged features were subsequently evaluated individually using Spearman correlation with Bonferroni correction for multiple comparisons (α < 0.05).

References:

1. Harner, T. *et al.* Global pilot study for persistent organic pollutants (POPs) using PUF disk passive air samplers. **144**, (2006).

2. Ménard, D. *et al.* Plant biomechanics and resilience to environmental changes are controlled by specific lignin chemistries in each vascular cell type and morphotype. *Plant Cell* **34**, 4877–4896 (2022).

3. Papazian, S. *et al.* Silicone Foam for Passive Sampling and Nontarget Analysis of Air. *Environ. Sci. Technol. Lett.* (2023) doi:10.1021/acs.estlett.2c00489.

Supplementary Figures

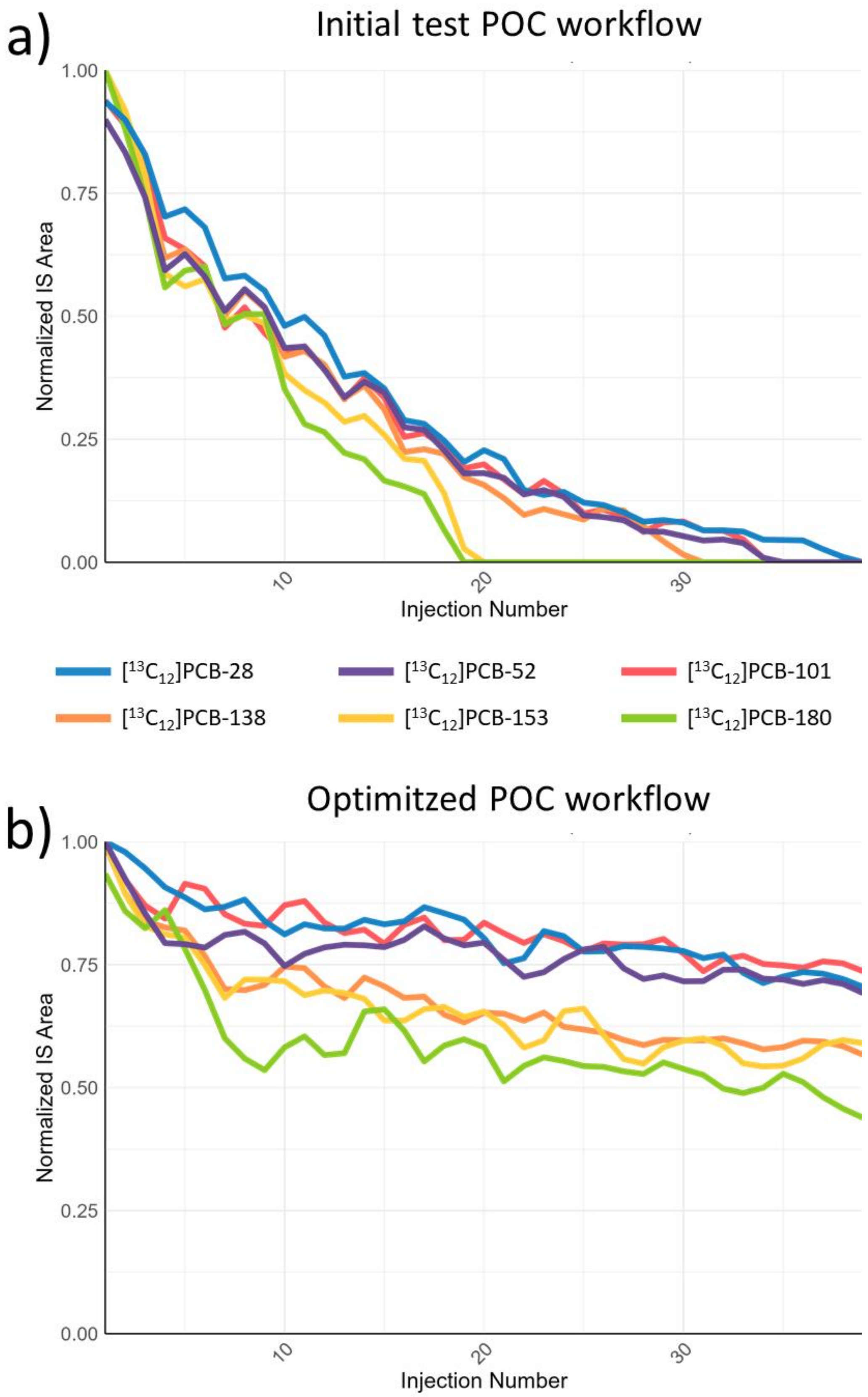

Figure S1. Normalized internal standard (IS) areas (relative to the highest area point) for different labelled PCBs after repeated reinjection of triplicate samples to assess the PDMS background effect on ion source and sensitivity. Panel (a) shows the results under initial cleaning and extraction conditions of the POC workflow, while panel (b) shows results after optimization. PDMS content in the extract initially caused significant signal decay and sensitivity loss, which was corrected after optimization, resulting in a normal decay over time in the GC analysis.

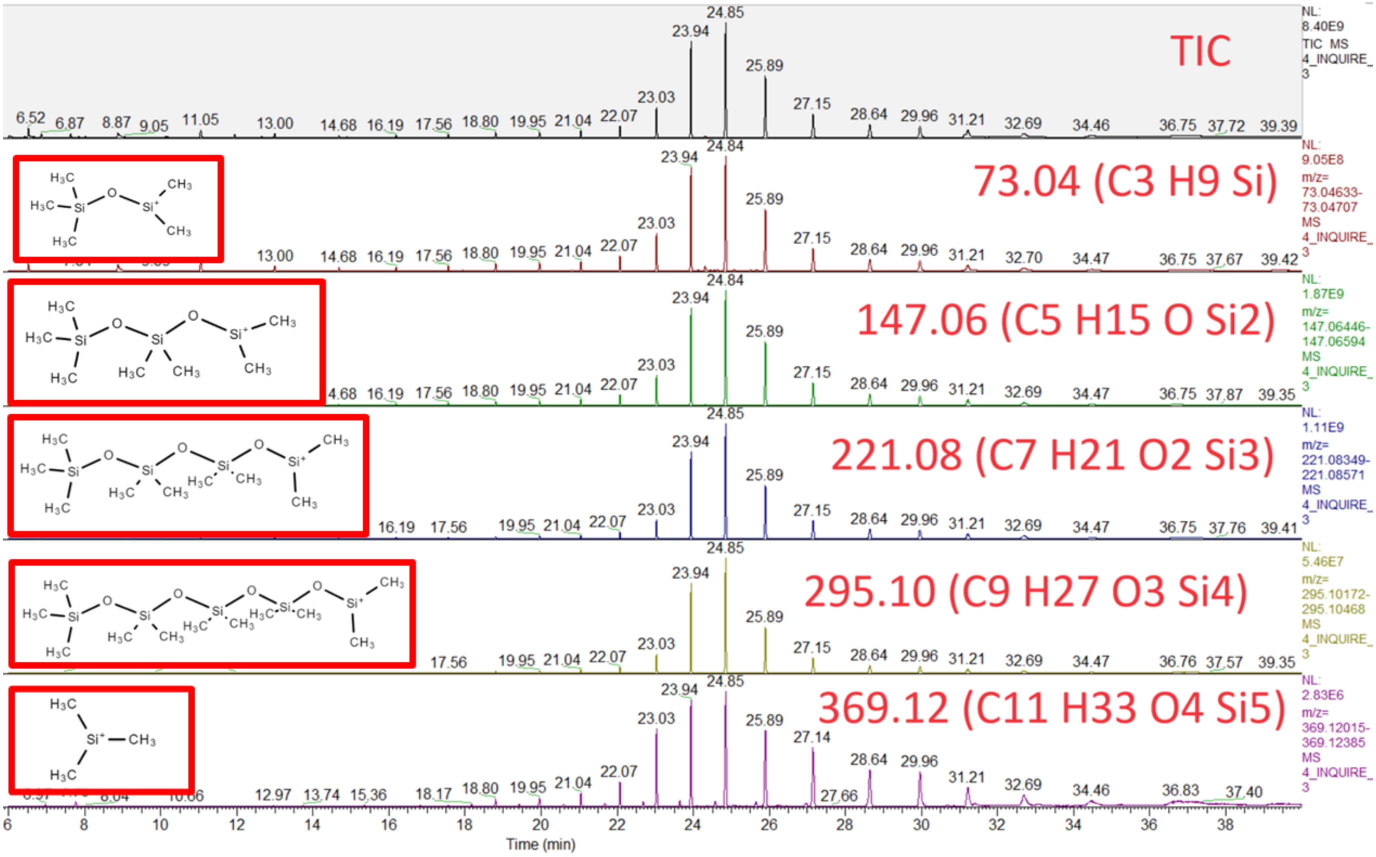

Figure S2. Example of a total ion chromatogram and extracted ion chromatograms of characteristic PDMS fragments, generating a PDMS background from an extracted foam analyzed by GC-HRMS.

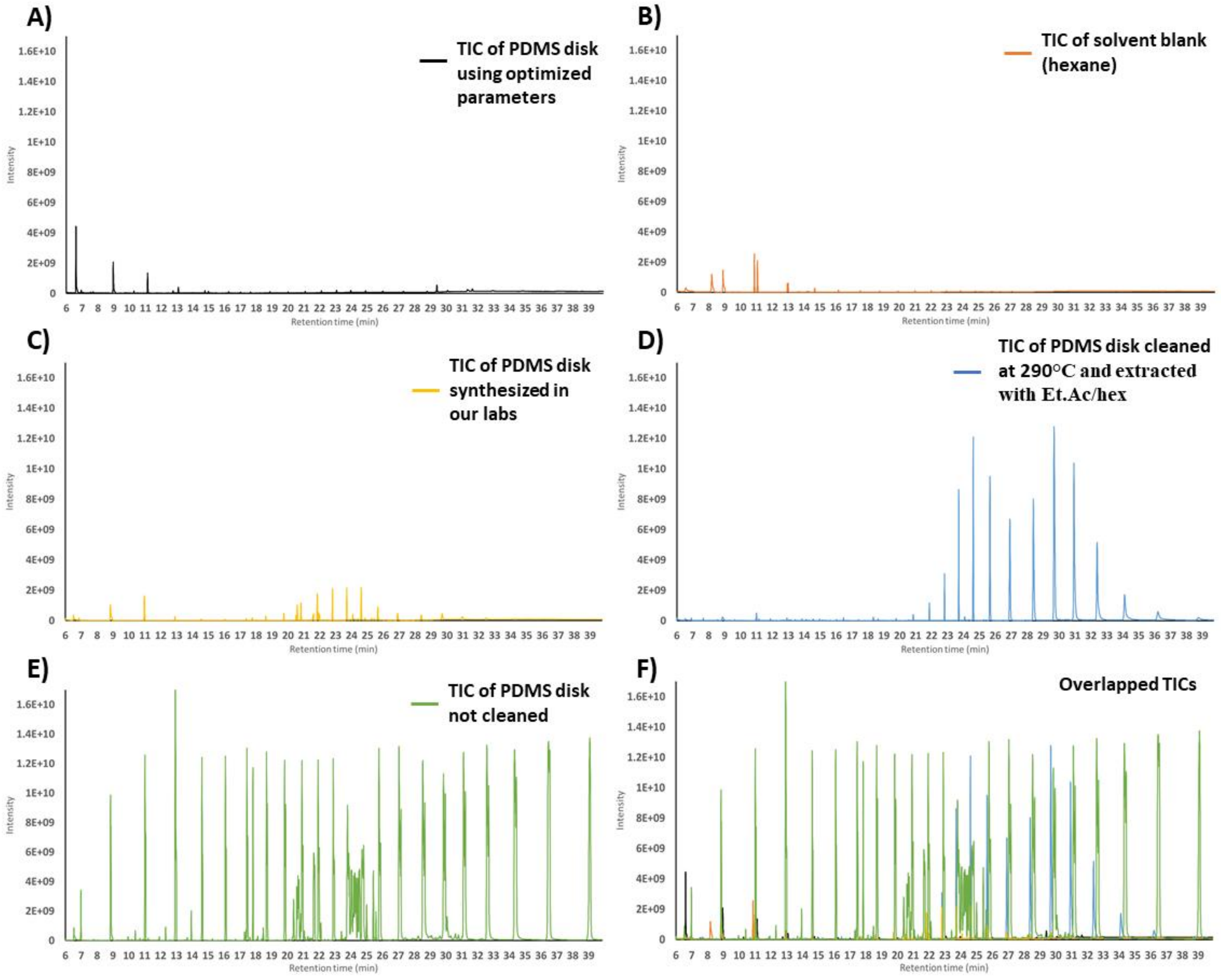

Figure S3. Total ion chromatogram (TIC) of PDMS foams cleaned and extracted using different conditions, and analyzed by GC-HRMS. TIC was used to monitor siloxane levels as it represented an overall sum of all PDMS fragment contributions (i.e. 73.0468 m/z (C_3H_9Si), 147.0656 m/z ($C_5H_{15}OSi_2$), 221.0844 m/z ($C_7H_{21}O_2Si_3$), or 369.1220 m/z ($C_{11}H_{33}O_4Si_5$)). (A) PDMS foam cleaned and extracted with the final optimized parameters. (B) Direct injection of hexane as solvent blank (C) Previously synthesized PDMS foam[3] cleaned and extracted with optimized parameters (D) PDMS foam baked at 290°C for 2h (instead of 250°C for 1h) and extracted with ethyl acetate and hexane (50:50) (instead of hexane 100%) (E) PDMS foam directly extracted without any cleaning protocol (F) Merged TICs for intensity comparison.

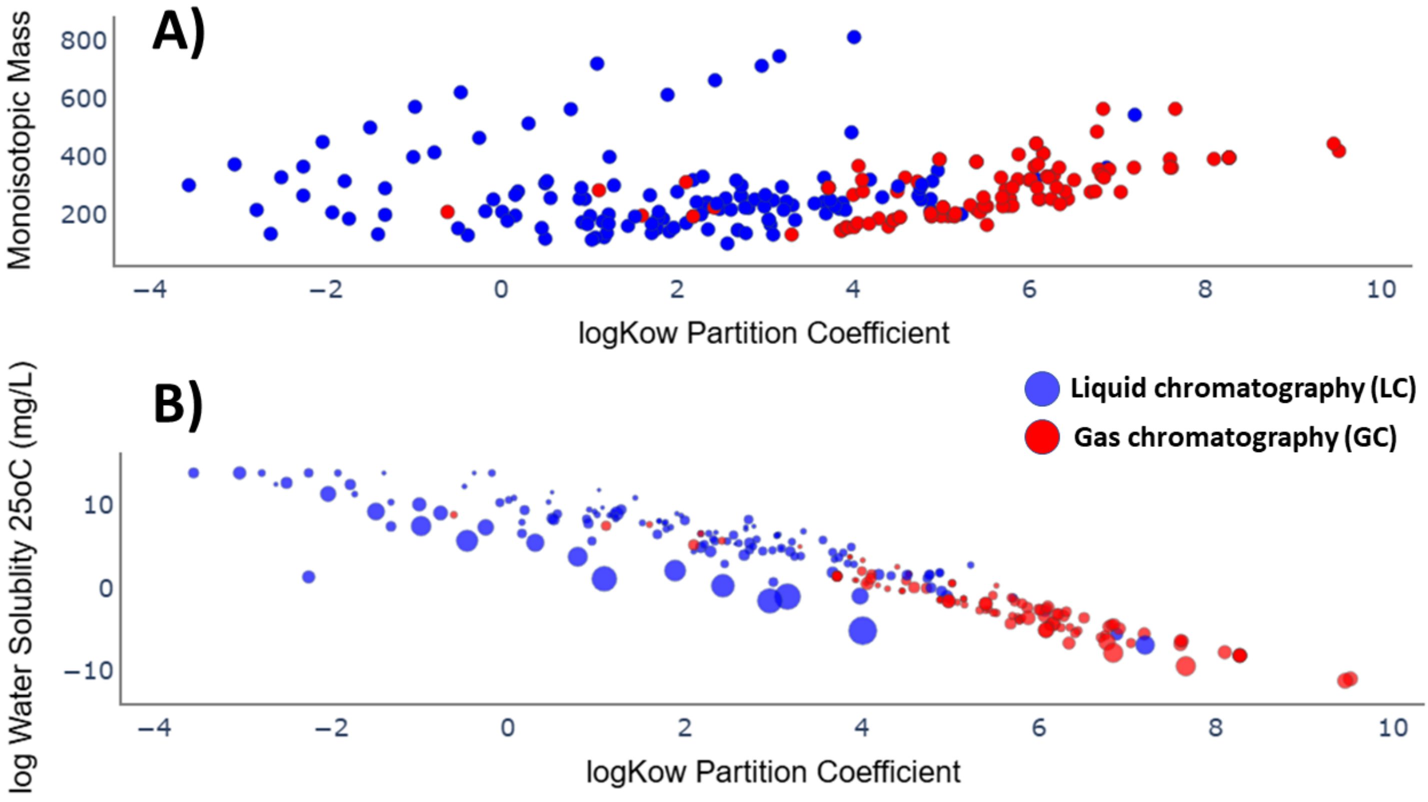

Figure S4. Distribution of selected multiclass targeted analytes categorized by A) monoisotopic mass versus log K_{ow} coefficient, and B) water solubility versus log Kow coefficient. This highlights the comprehensive coverage of physicochemical properties across the analytes.

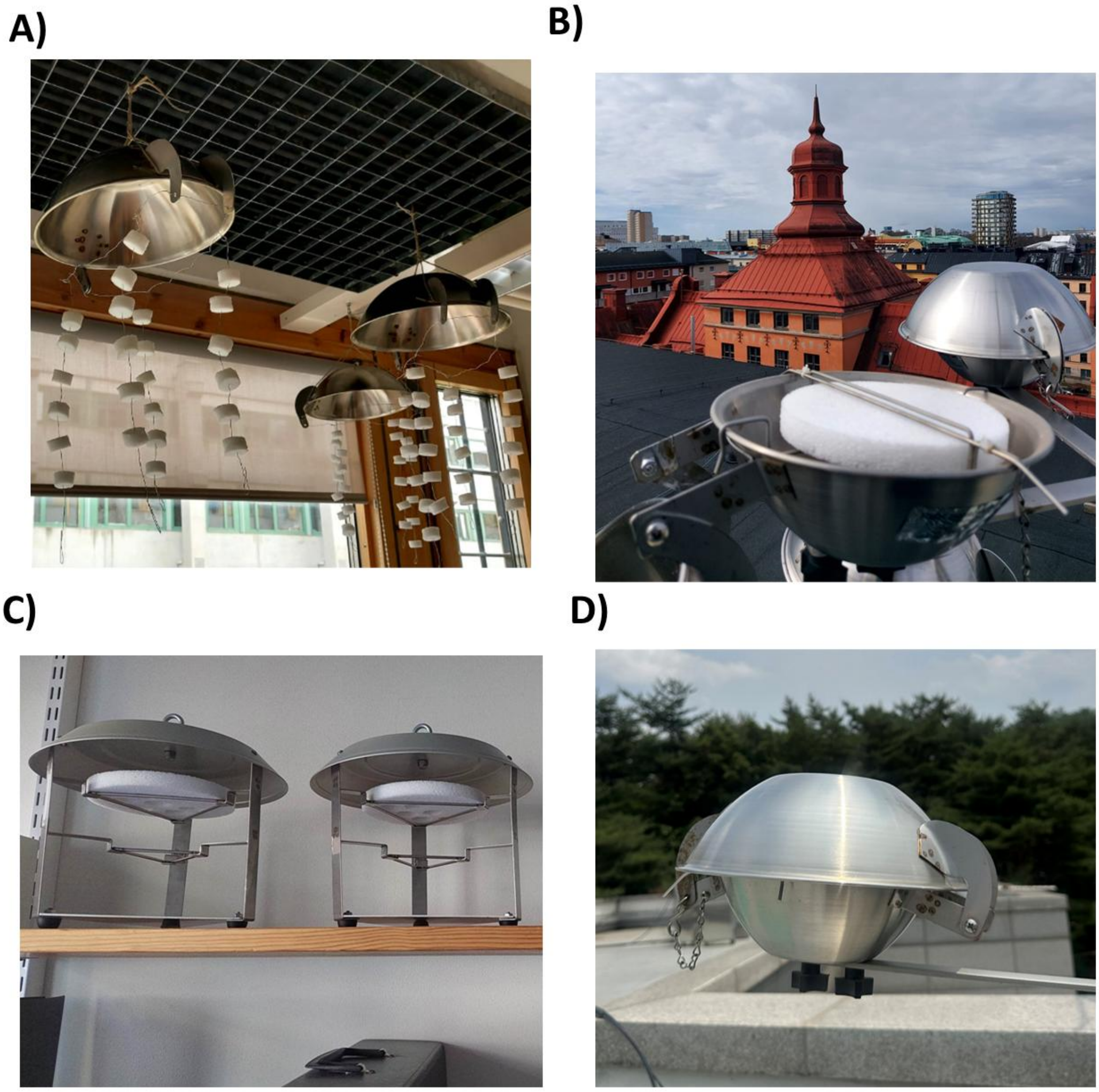

Figure S5. Pictures of the different PDMS foam passive samplers during (A) deployment 1 in an office at Stockholm University campus, (B) deployment 2 in an outdoor rooftop station in Södermalm, Stockholm, Sweden, (C) deployment 2 in another indoor office at Stockholm University campus, and (D) deployment 3 in a rooftop station in Seoul, Republic of Korea.

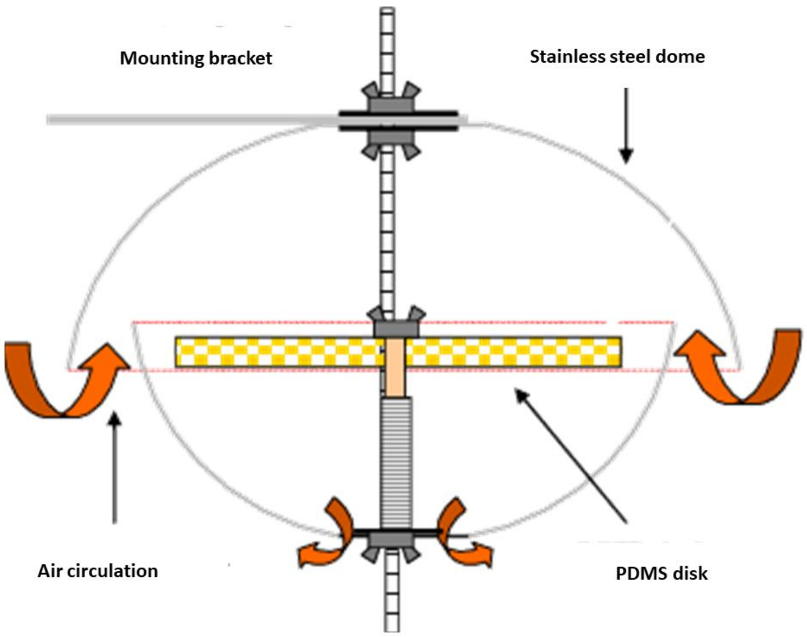

Figure S6. Schematic of the double bowl metallic housing used to deploy PDMS foams in outdoor environments. It shows the air flow in between bowls, and the PDMS foam placement.

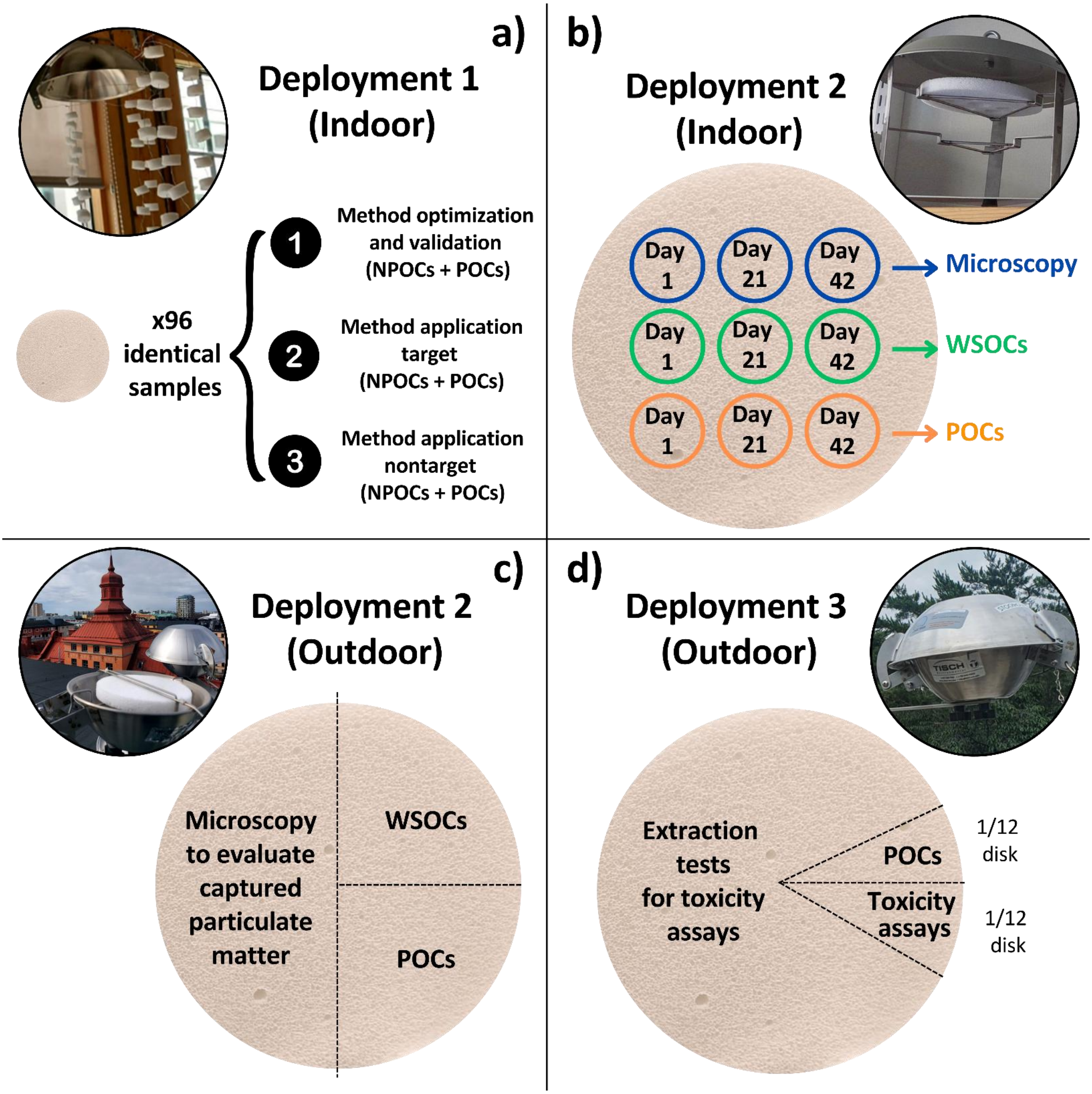

Figure S7. Schematic representation of the different deployments and the different uses/applications of the PDMS foams in each case. (A) 96 small PDMS foam disks were deployed in the same indoor space, and were used to optimize and validate NPOCs and POCs methods, and to perform target and nontarget analysis in real samples. (B) Indoor, a cork borer was used to poke holes in big PDMS disks after 1, 21, and 42 days of deployment to assess linearity uptake of PM (microscopy), and water-soluble (WSCOs) and polar (POCs) organic compounds. (C) Outdoor, big PDMS disks were deployed for 42 days, and cut in different parts to assess PDMS uptake capacity of PM and organic chemicals in ambient air. (D) Outdoor PDMS foam disks were primarily used to evaluate various extraction protocols for assessing extract cytotoxicity in human lung fibroblasts. Finally, one-twelfth of the disk was used to measure lung fibroblast viability following exposure to the PDMS extracts.

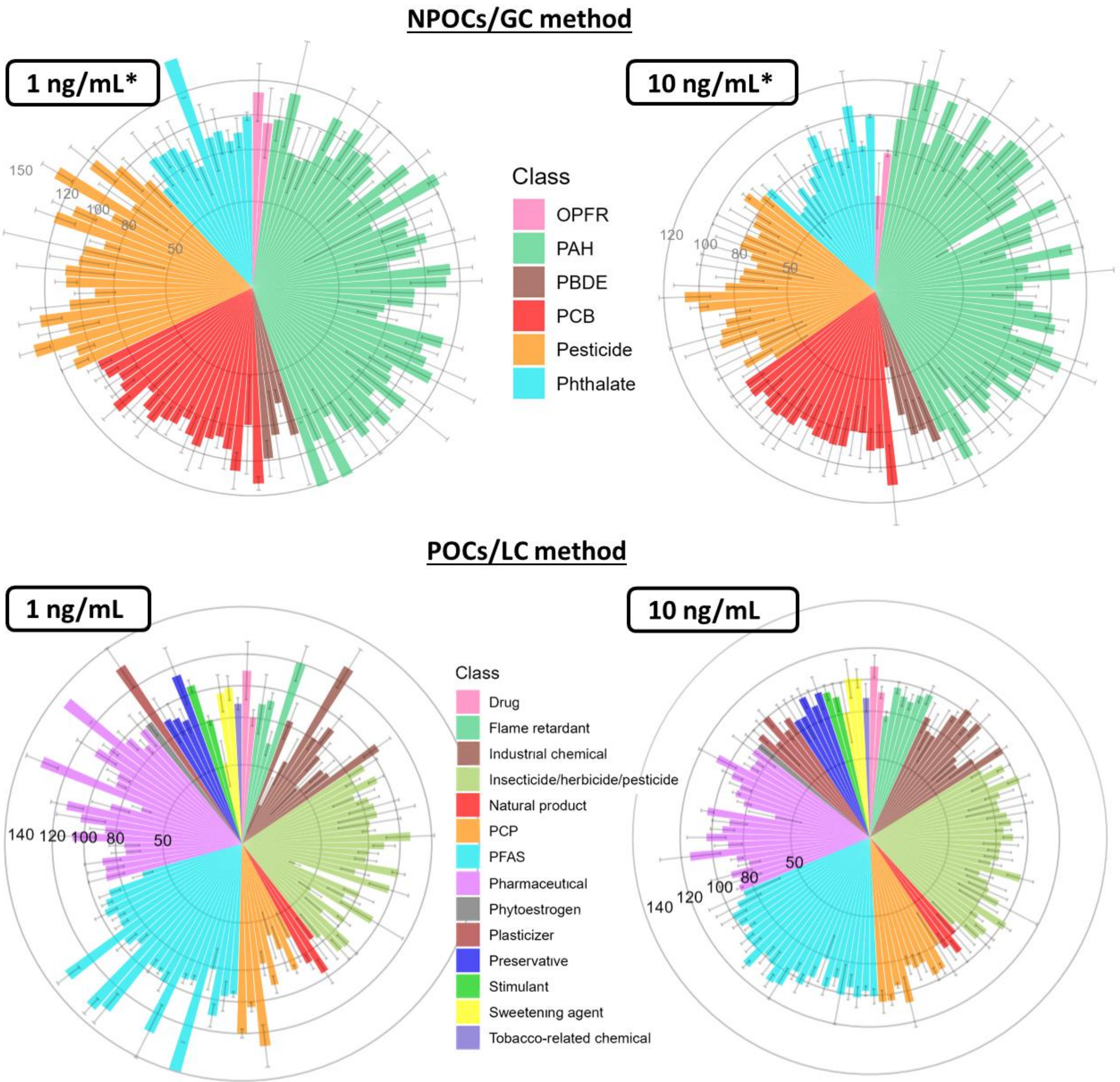

Figure S8. Absolute recoveries of NPOCs (104 analytes) and POCs (118 analytes) using optimized methods for PDMS passive sampler extraction at two different spiking levels (1 ng/mL and 10 ng/mL). *For NPOCs method, phthalate and PBDEs chemical families were spiked at 2 and 20 ng/mL.

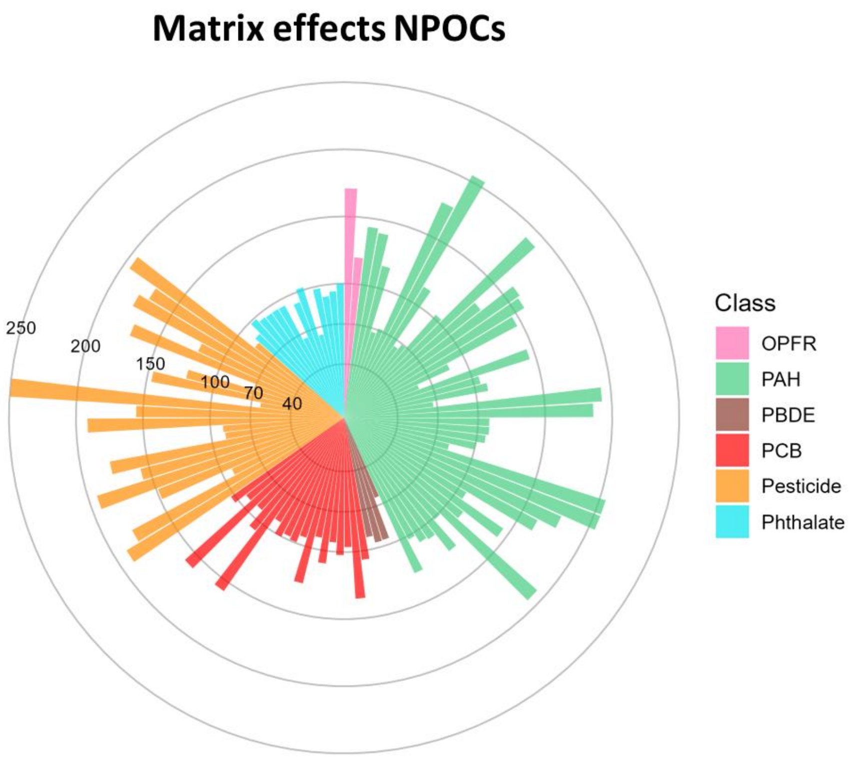

Figure S9. Matrix effects for each target analyte using the NPOCs method.

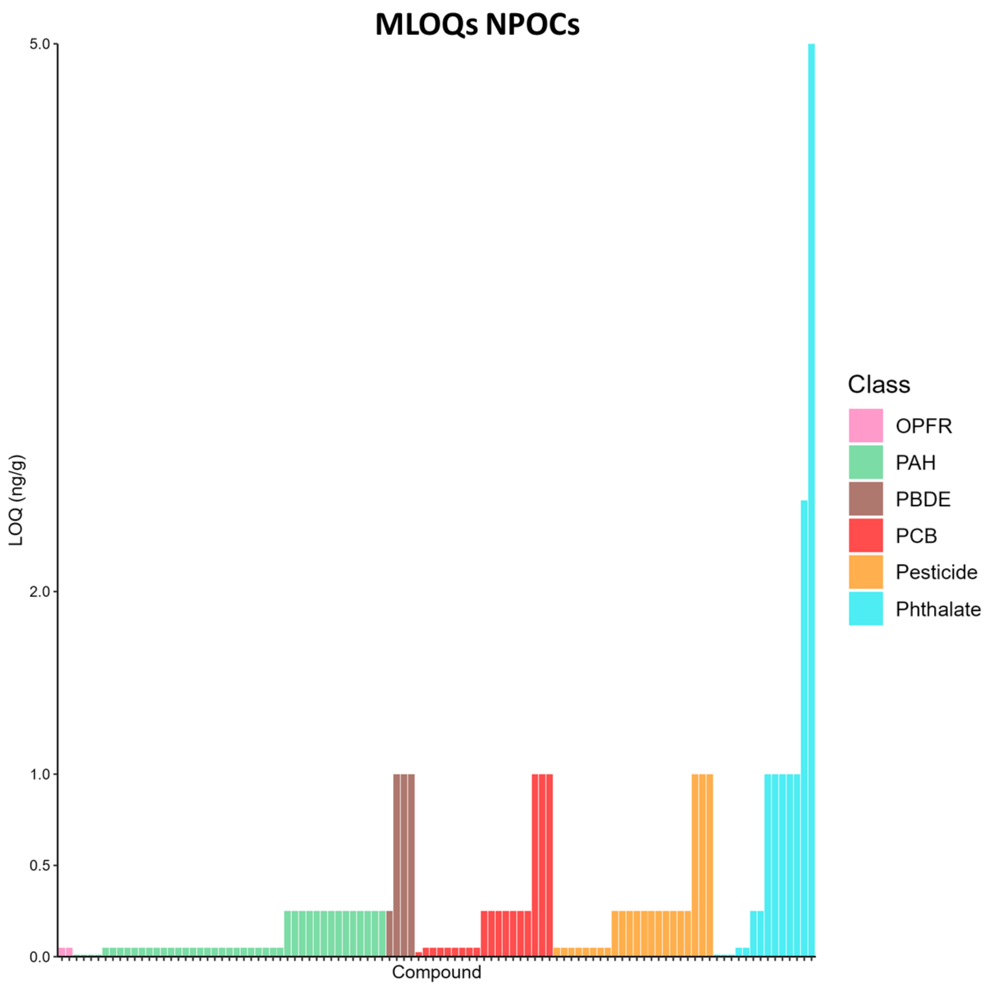

Figure S10. MLOQs of each target analyte in NPOCs method.

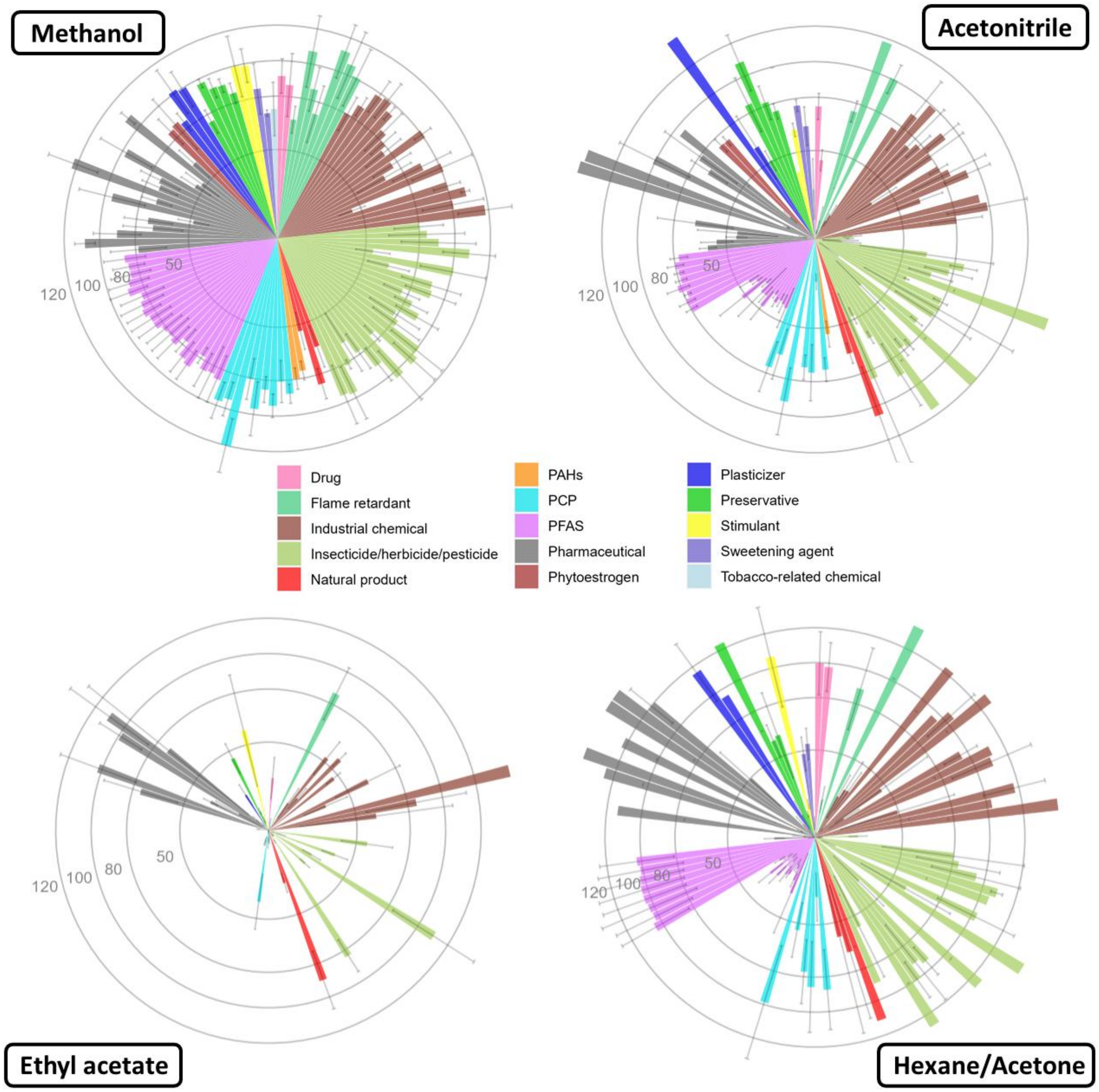

Figure S11. Absolute recoveries for POCs target analytes after extraction with different organic solvents.

A)

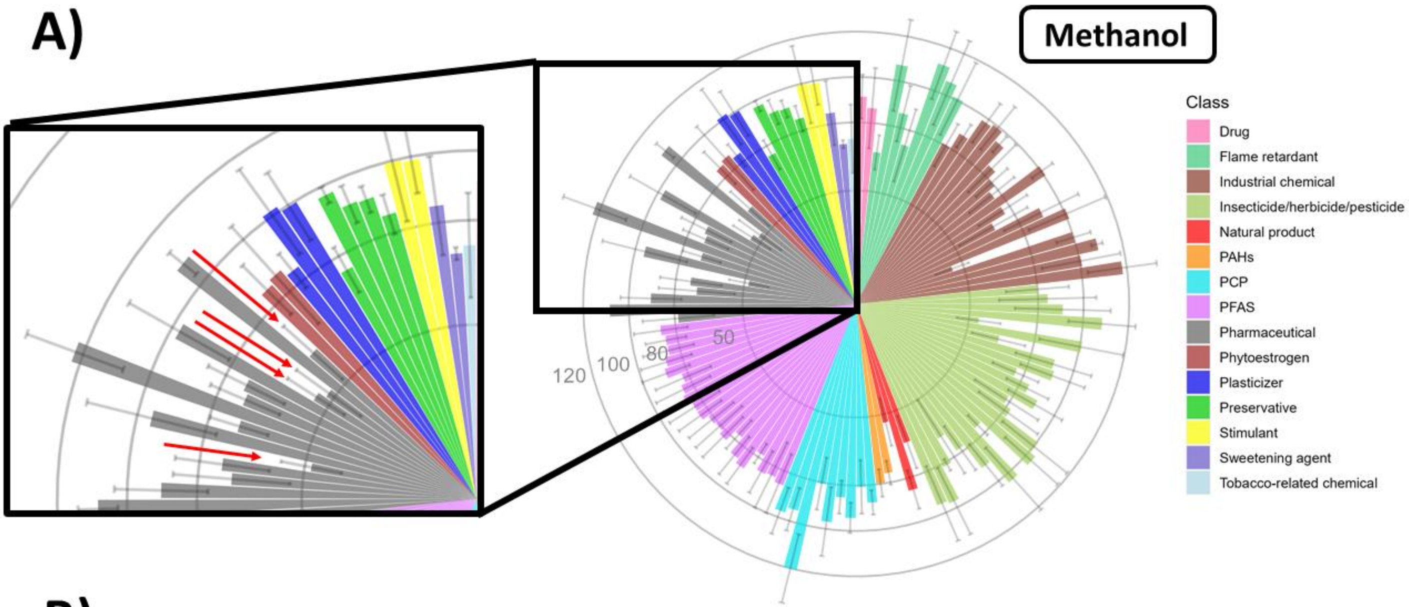

B)

Target analyte	Chemical Family	Recovery	RSD%	Mass	Log kow	Water solubility	Ionization
Codeine	Pharmaceutical	48.1	8.9	299.15	1.28	12150	POS
Sulfamethazine	Pharmaceutical	54.5	9.7	278.33	0.19	11270	POS
Sulfamethoxazole	Pharmaceutical	52.0	11.4	253.28	0.89	3942	POS
Trimethoprim	Pharmaceutical	62.8	11.4	290.32	0.91	2334	NEG

Figure S12. a) Specific analytes with low recoveries following methanol extraction were identified. b) Their physicochemical properties were then assessed to uncover potential trends. The analysis revealed that analytes with the lowest recovery rates exhibited notably high water solubility, highlighting the importance of adding water to the final extract before injection.

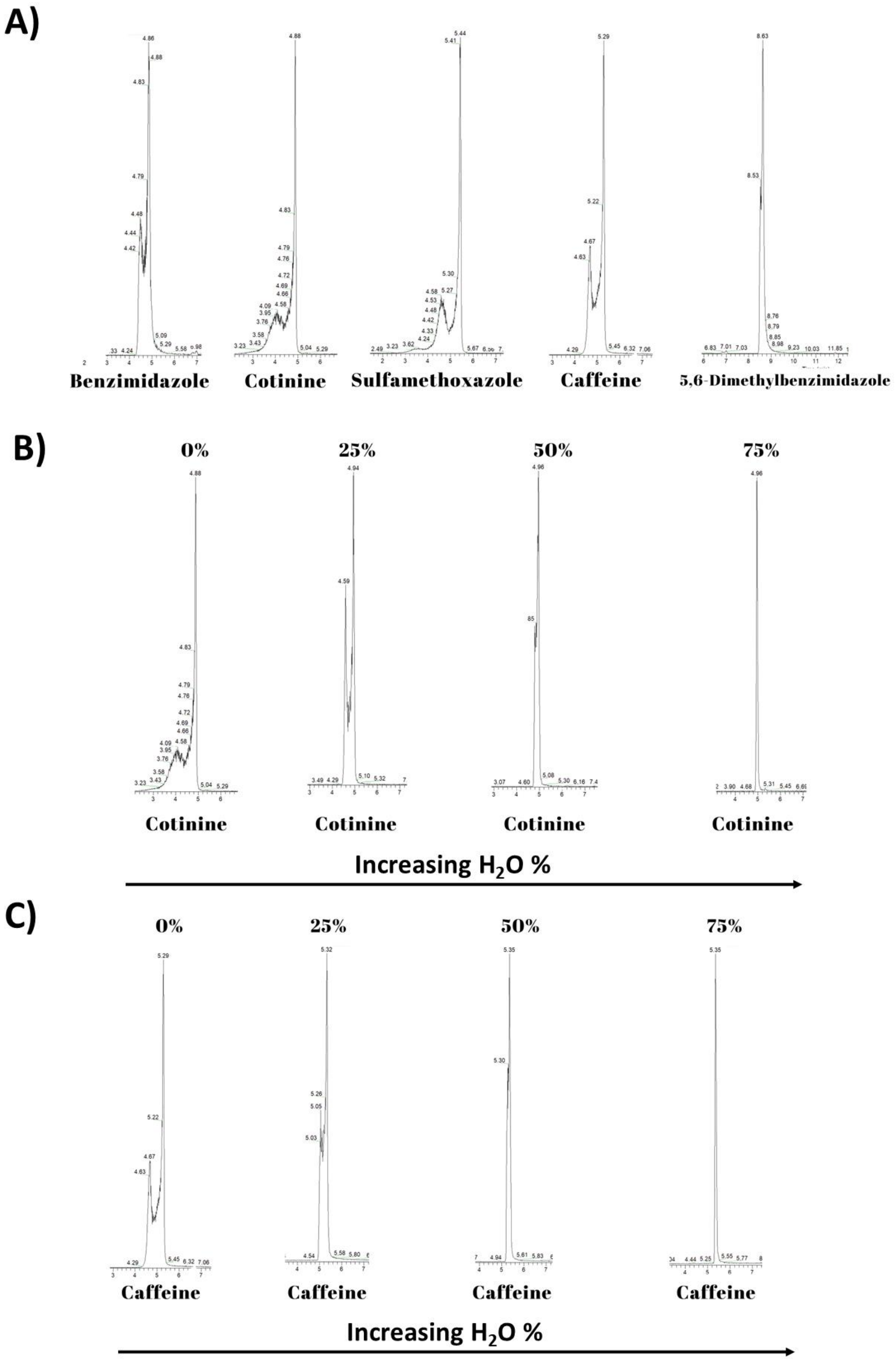

Figure S13. A) Extracted chromatograms of selected analytes after extraction and injection of 100% methanol. B) Extracted chromatograms of cotinine when increasing the water content in the extract. C) Extracted chromatograms of caffeine when increasing the water content in the extract.

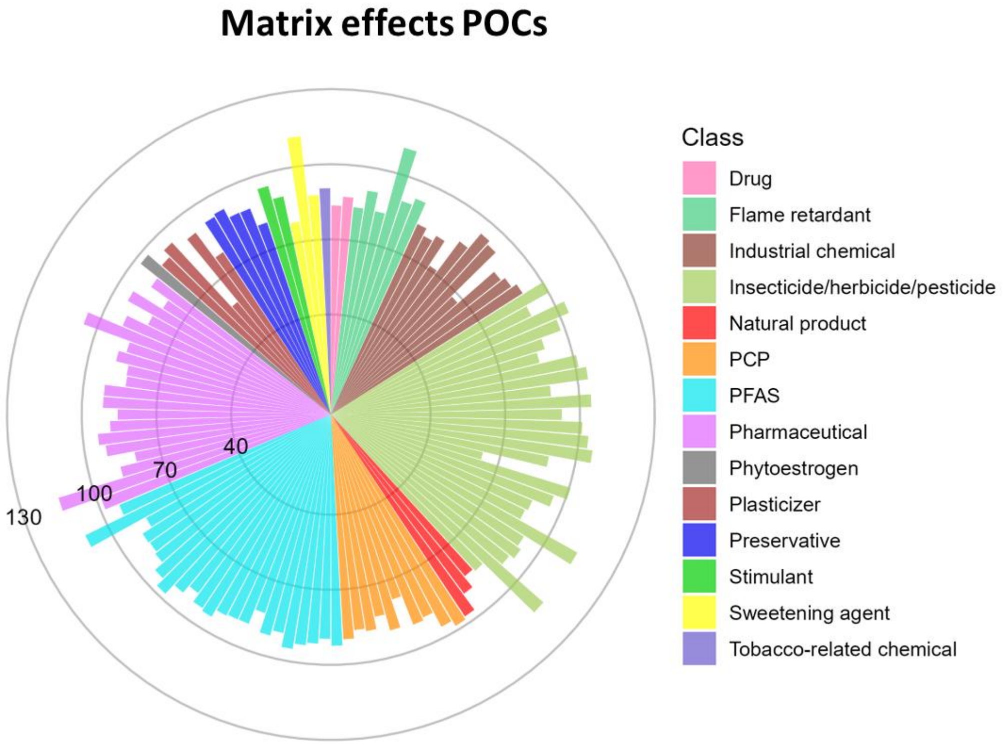

Figure S14. Matrix effects for each target analyte using the POCs method.

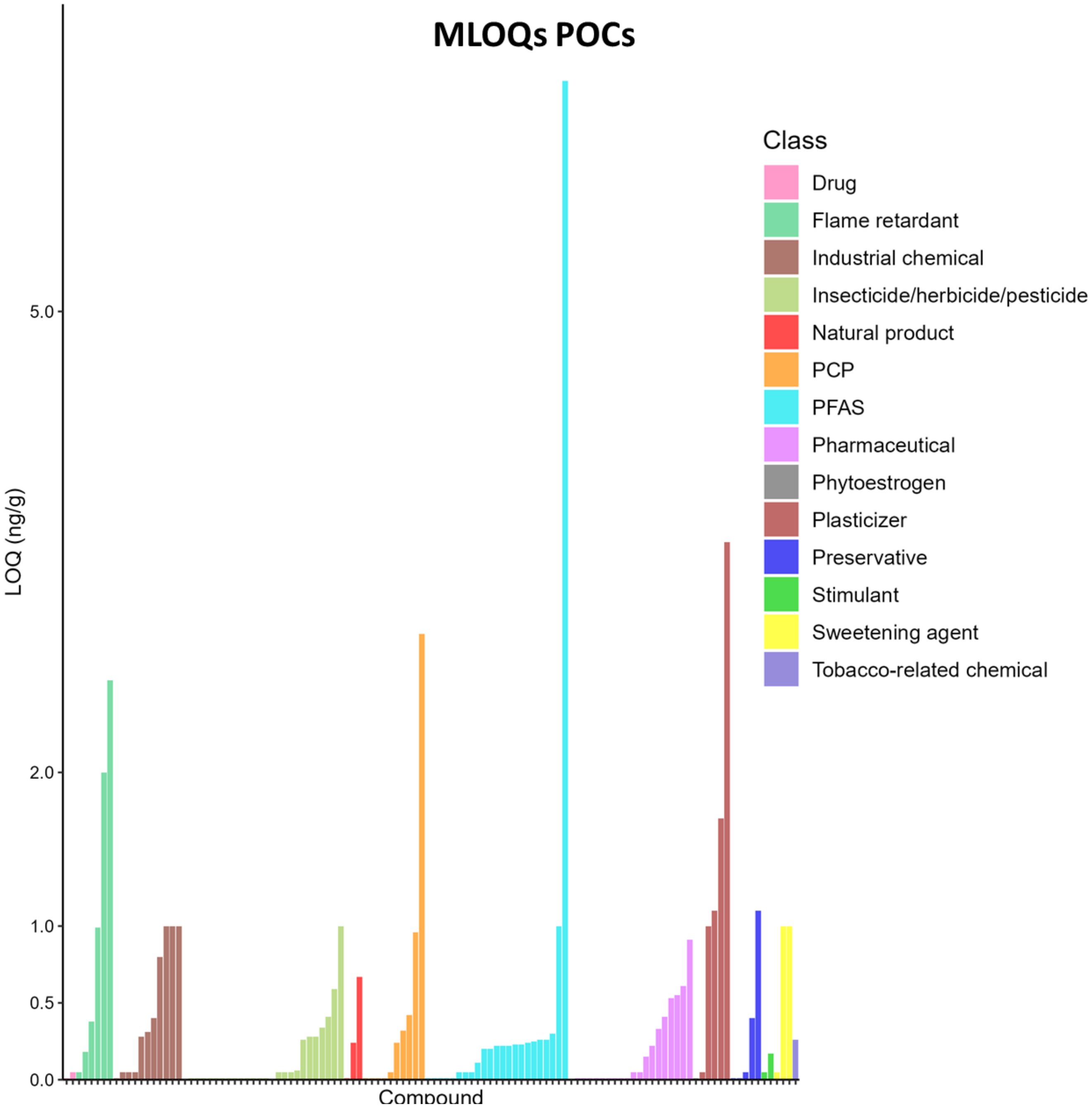

Figure S15. MLOQs of each target analyte in POCs method.

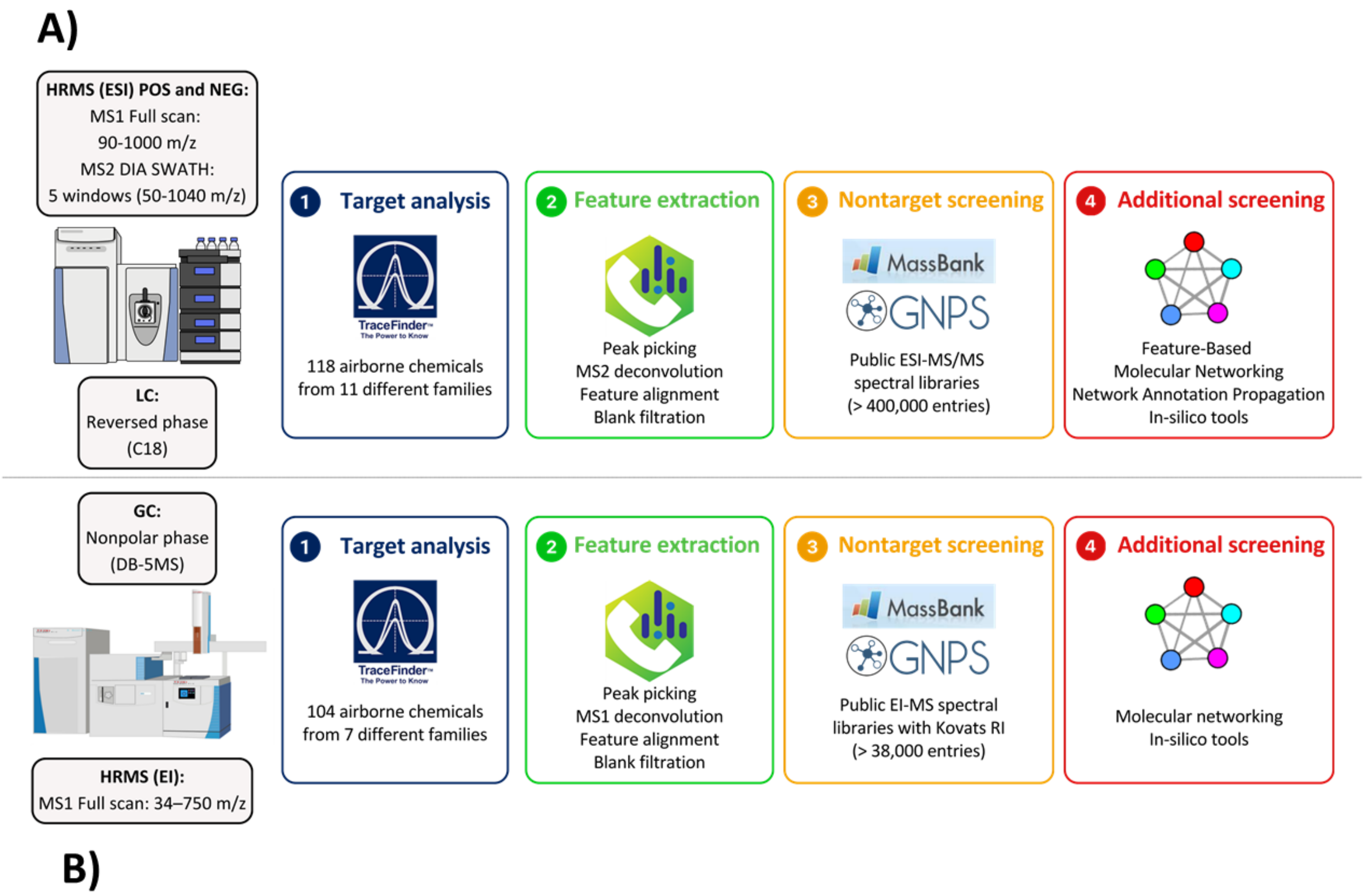

Figure S16. Workflows used for A) POCs and B) NPOCs after PDMS extraction and analysis.

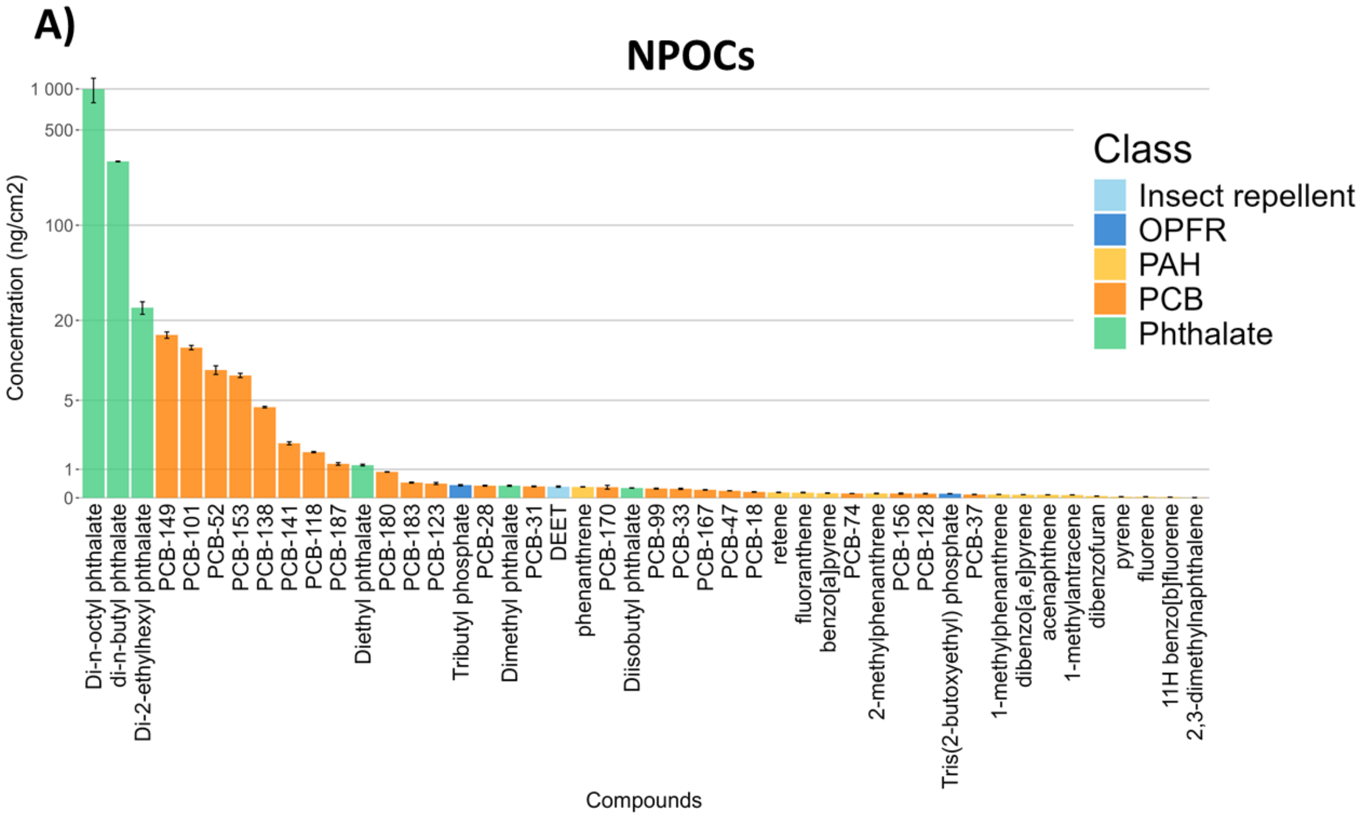

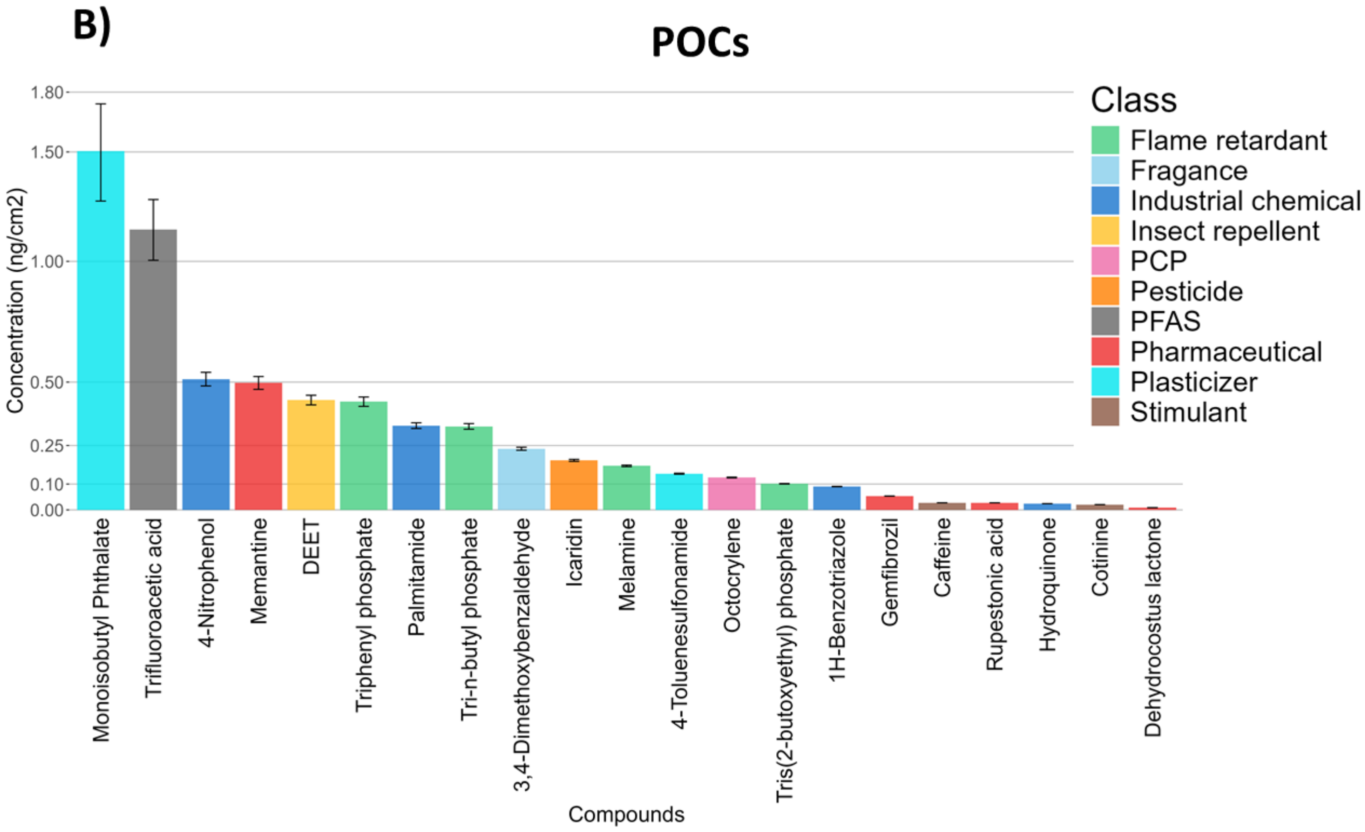

Figure S17. Measured concentration of A) NPOCs and B) POCs analytes using target analysis and normalized by surface of PDMS foam passive sampler (logarithmic scale) from deployment 1 (indoor).

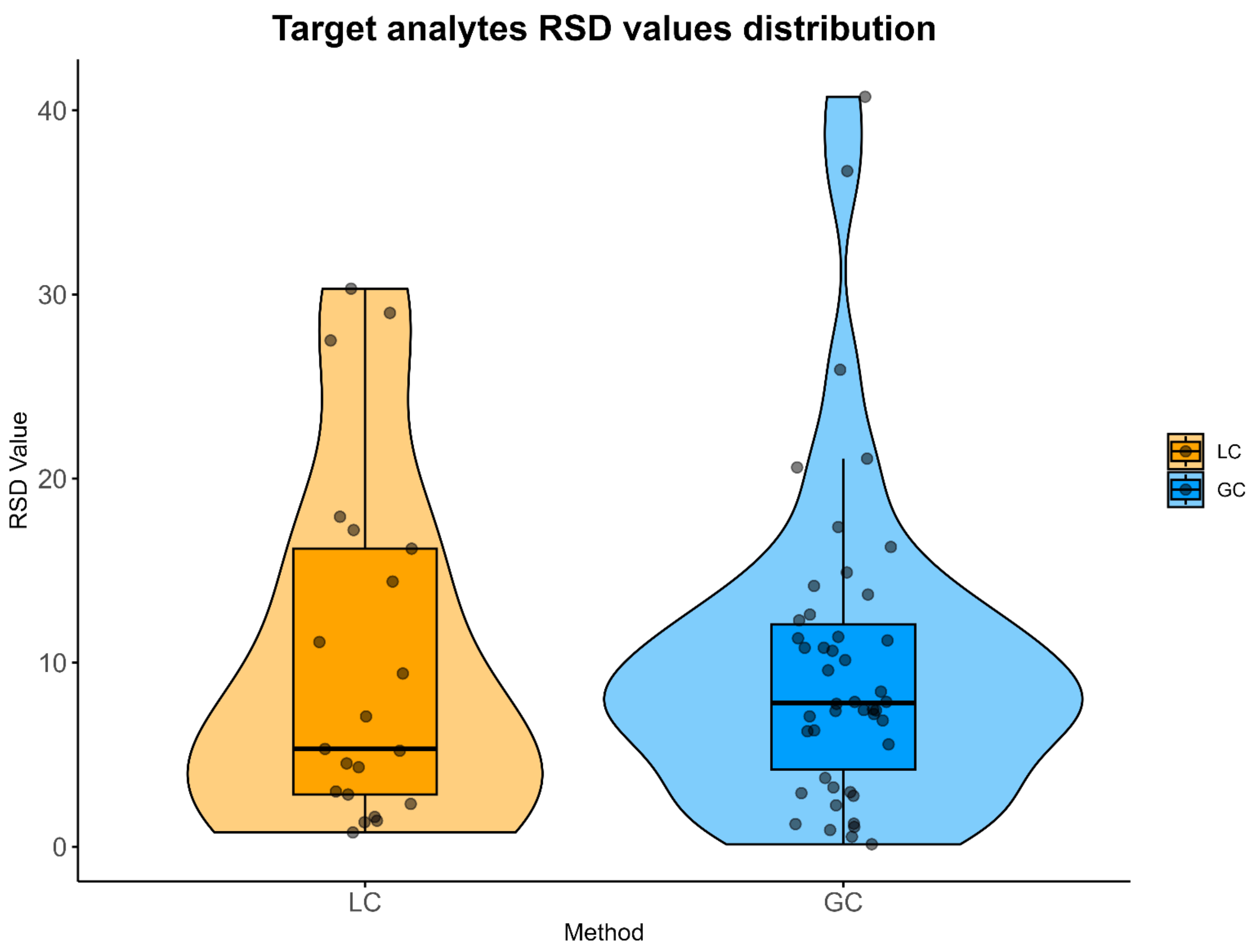

Figure S18. Raincloud plots illustrating the distribution of RSD% values for target analytes measured in triplicate PDMS foams. The plot combines a boxplot, violin plot, and individual data points to show spread and central tendency of the RSD values for each extraction method (left) LC and (right) GC. The horizontal line represents the median value, and individual points indicate raw data values.

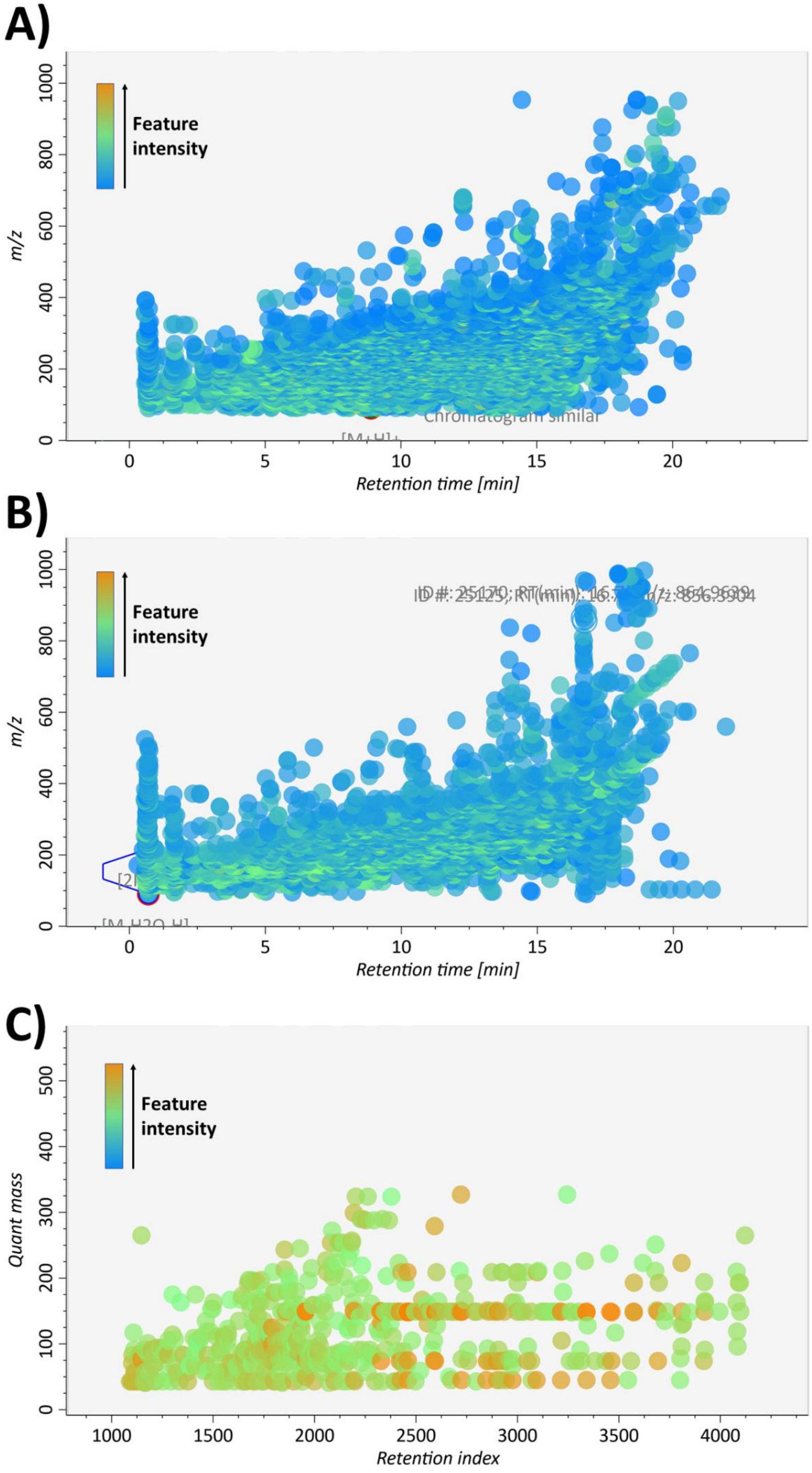

Figure S19. Extracted features plotted by retention time/index and mass for (A) NPOCs in ESI+, (B) NPOCs in ESI-, and (C) POCs from non-target analysis. Feature intensity is represented by color.

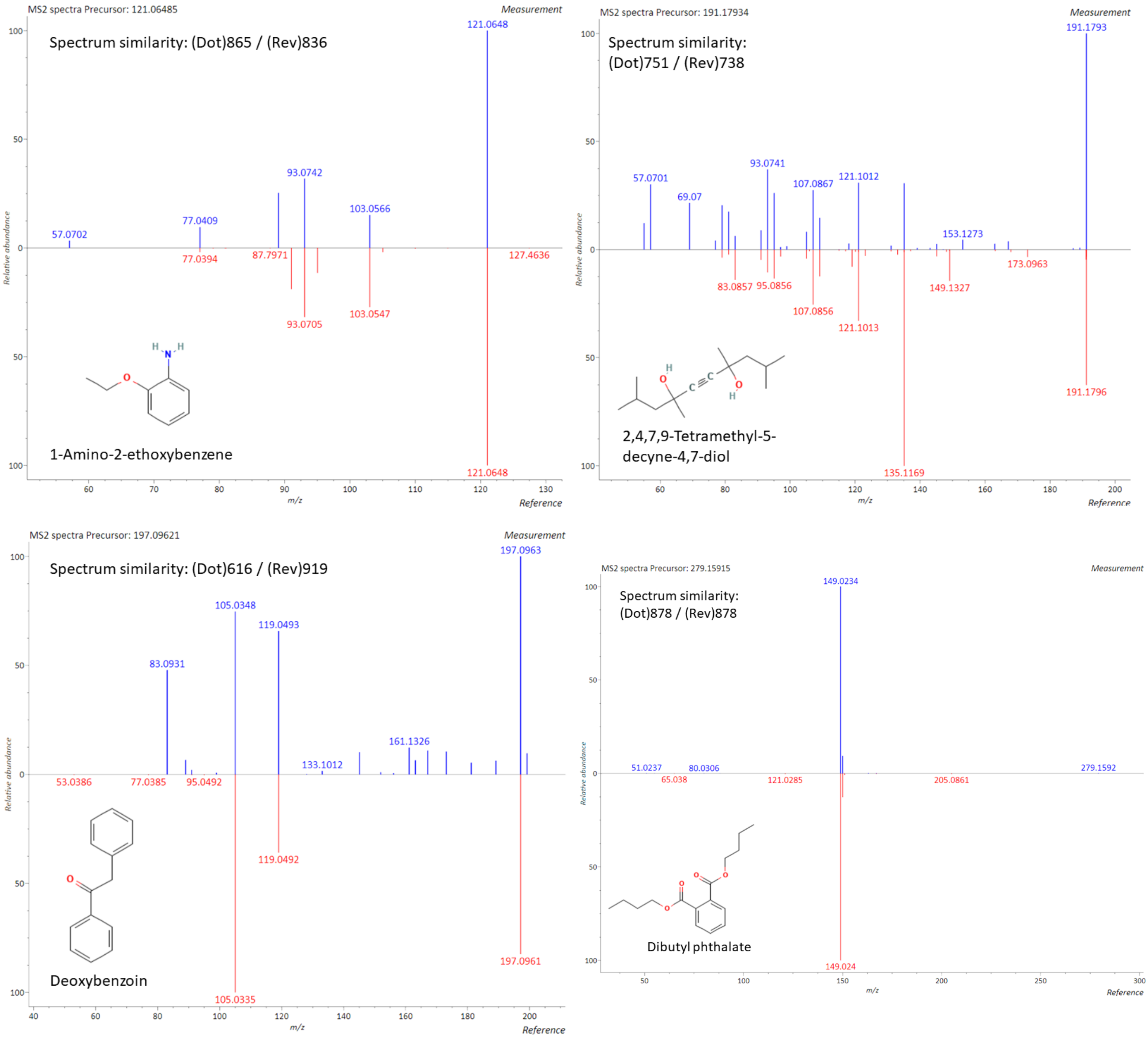

Figure S20. Spectral matches between LC-HRMS publicly available databases ("Reference," red spectrum) and our sample data ("Measurement," blue spectrum), along with their spectral similarity. Continued on the next page.

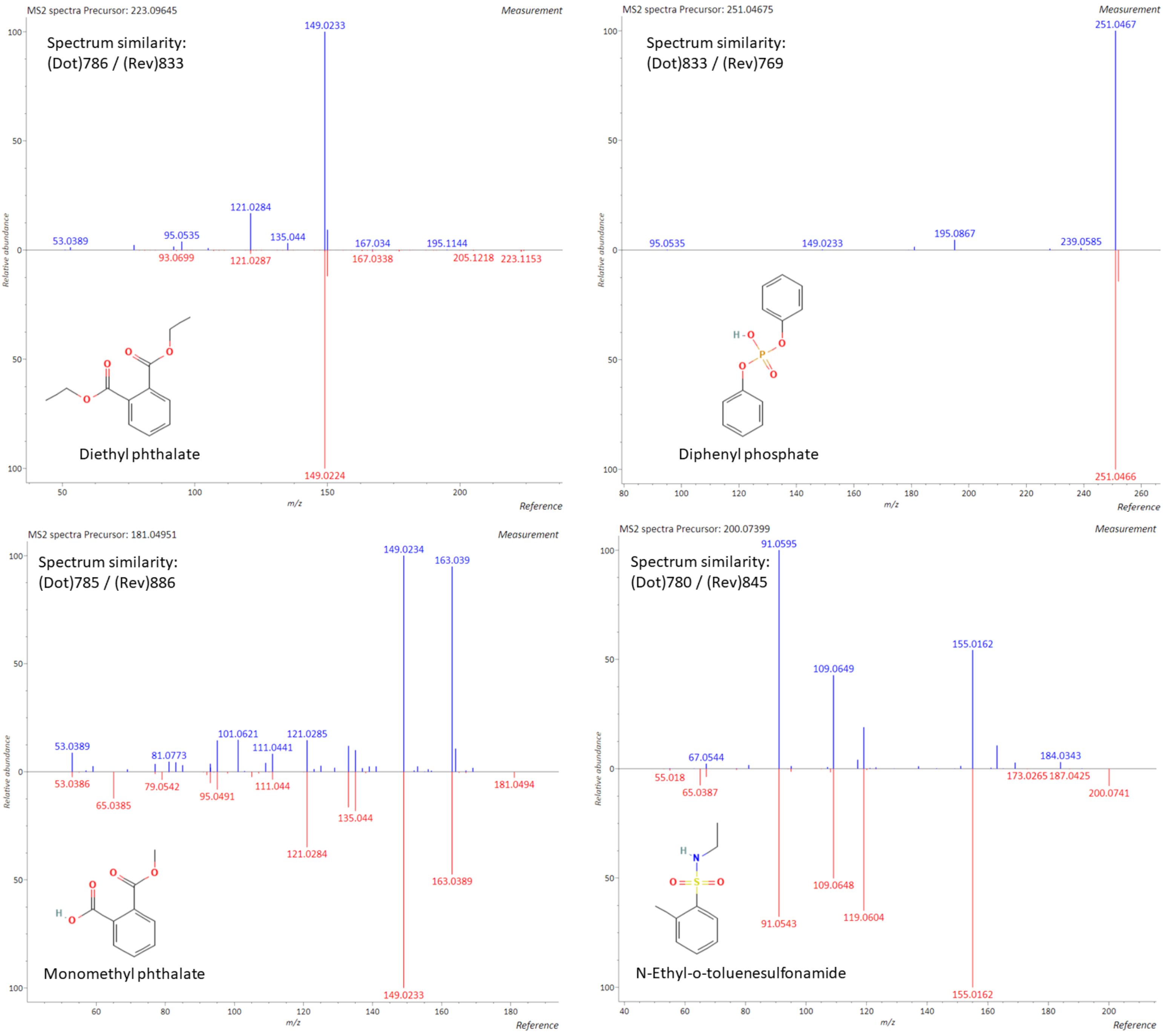

Figure S20. Continued.

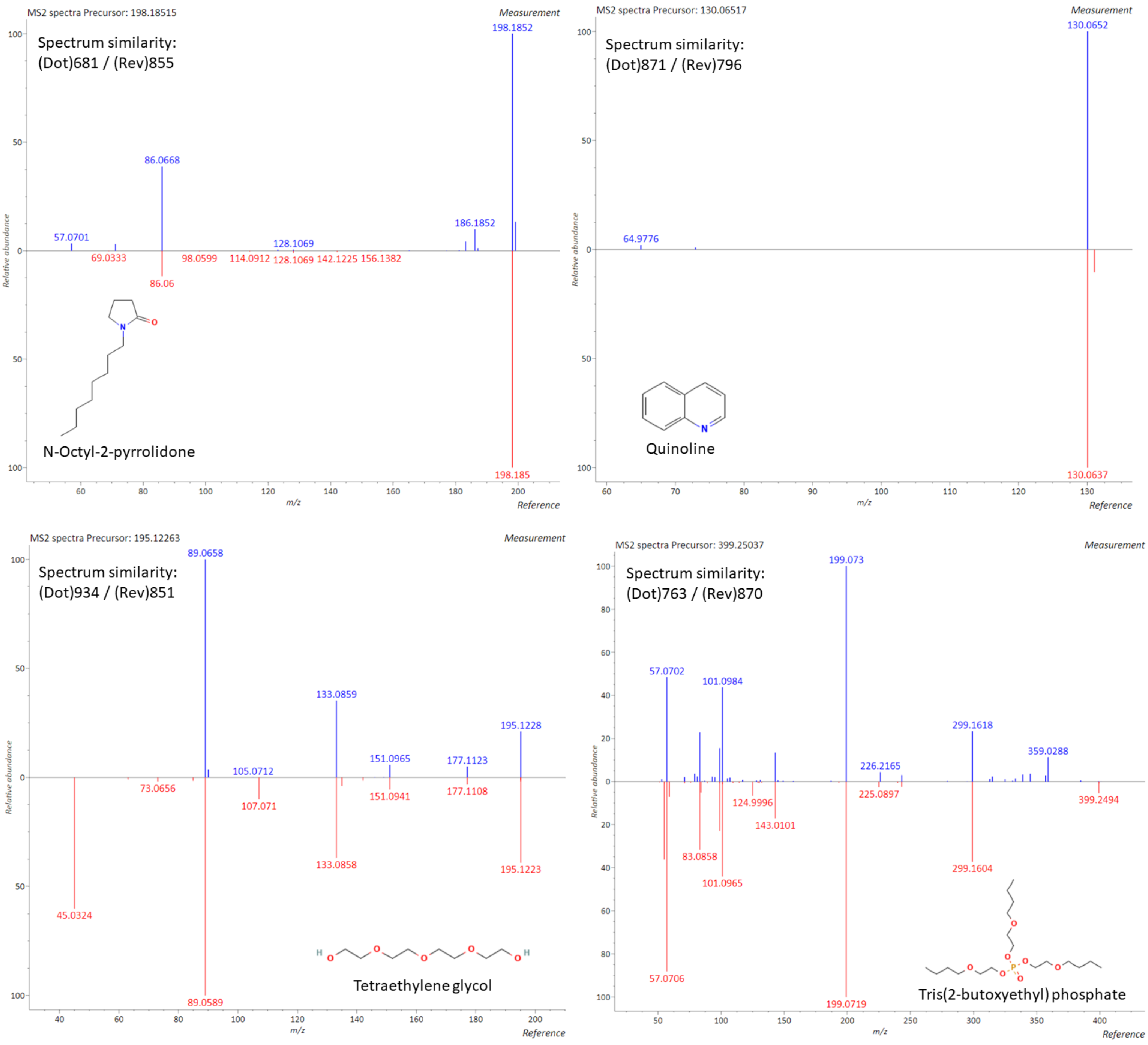

Figure S20. Continued.

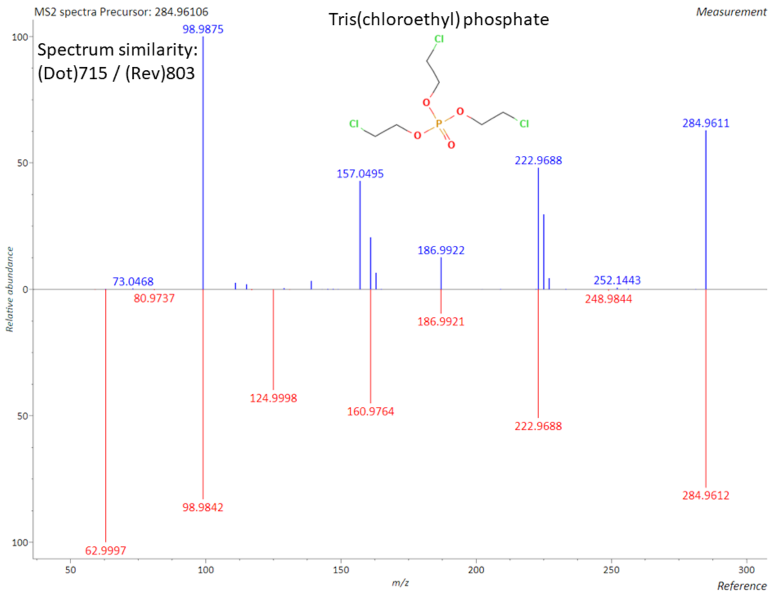

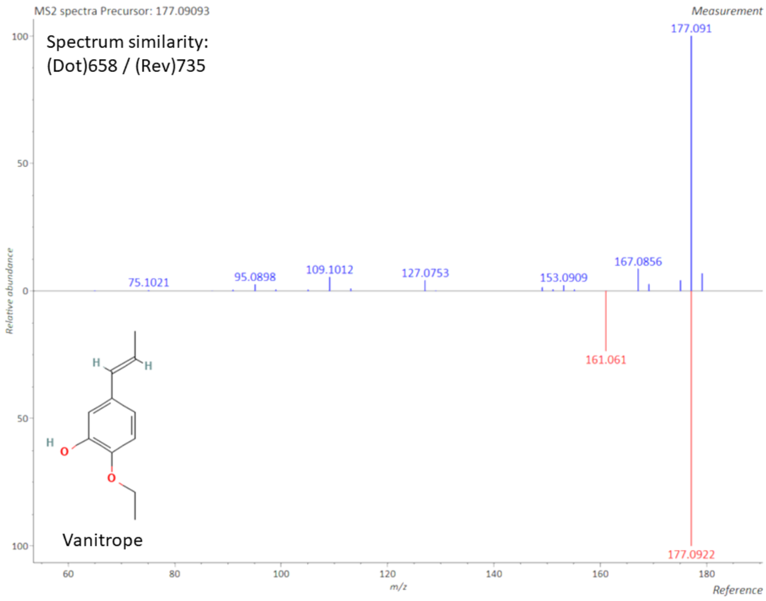

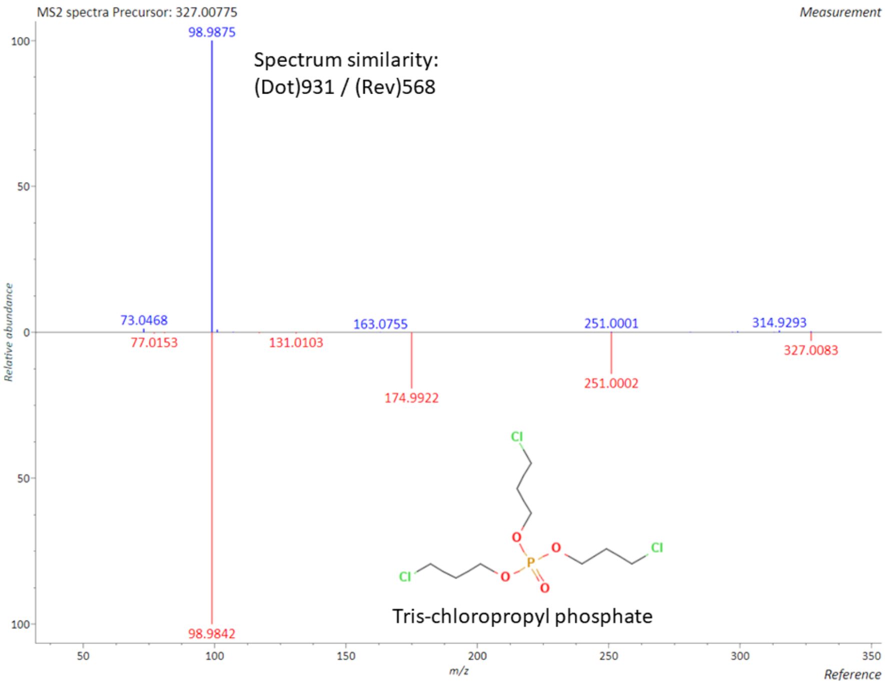

Figure S20. Continued.

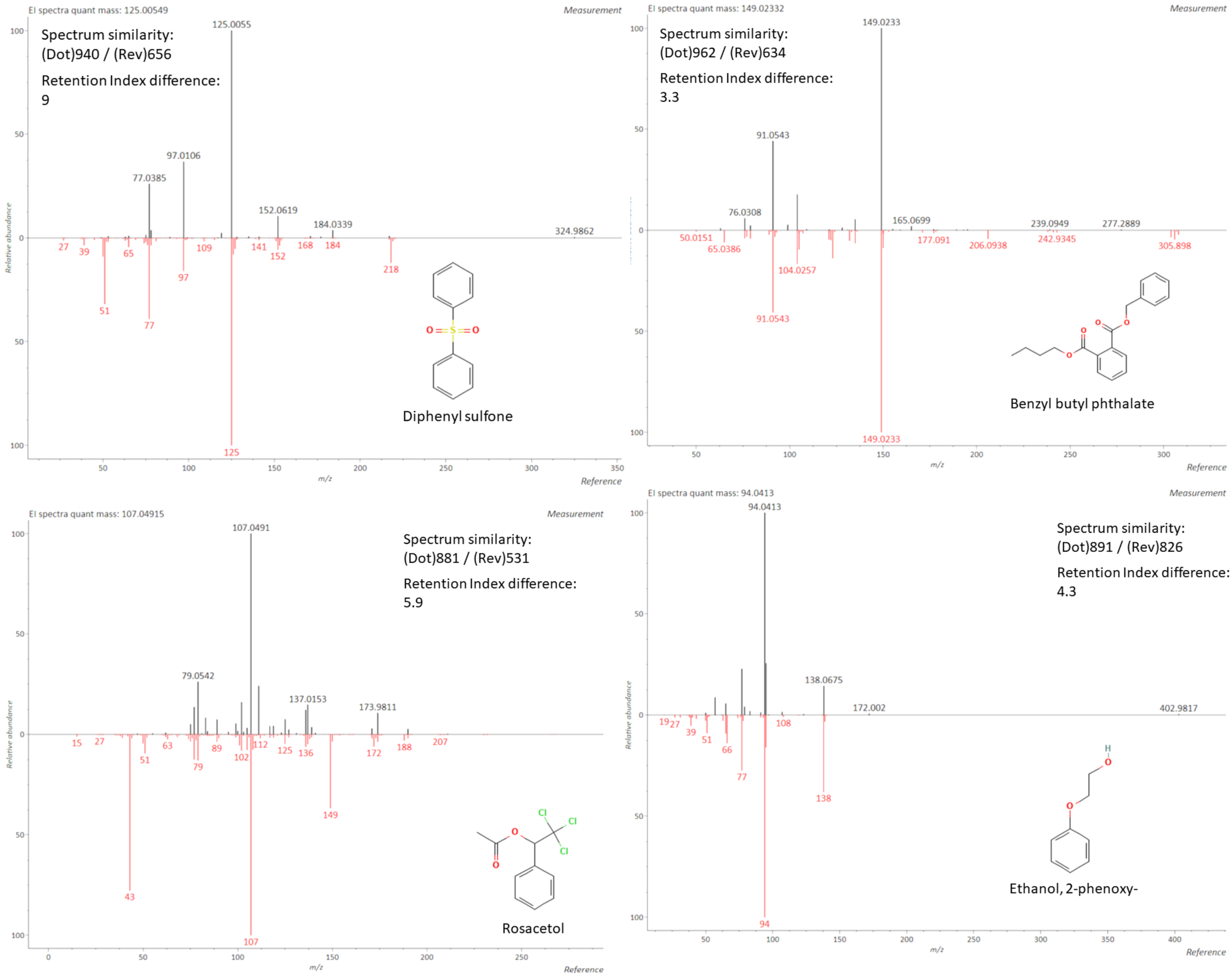

Figure S21. Spectral matches between GC-MS publicly available databases ("Reference," red spectrum) and our sample data ("Measurement," blue spectrum), along with their spectral similarity. Continued on the next page.

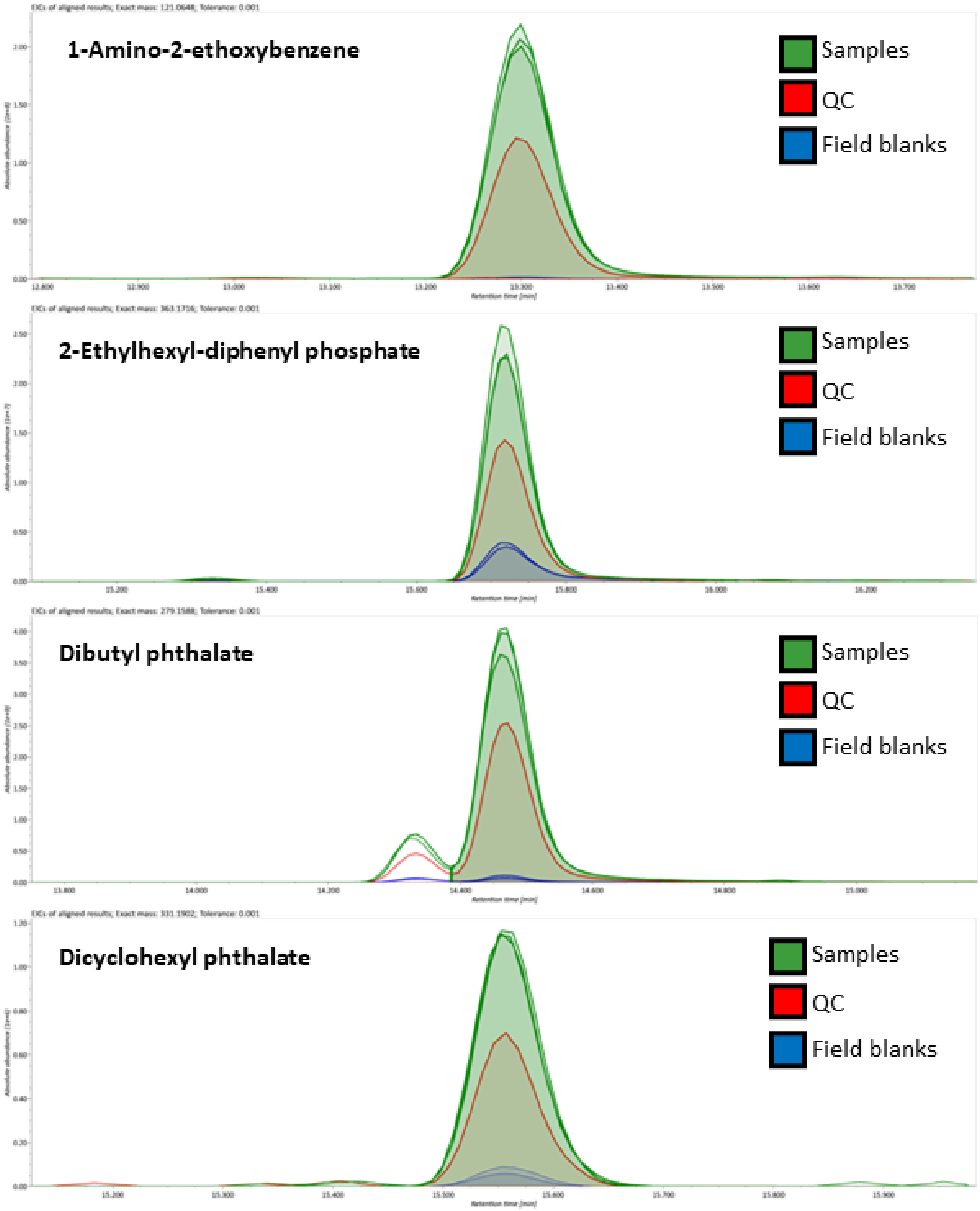

Figure S22. Examples of chromatographic peaks of LC-HRMS annotations in samples (green), QC (red) and field blanks (blue).

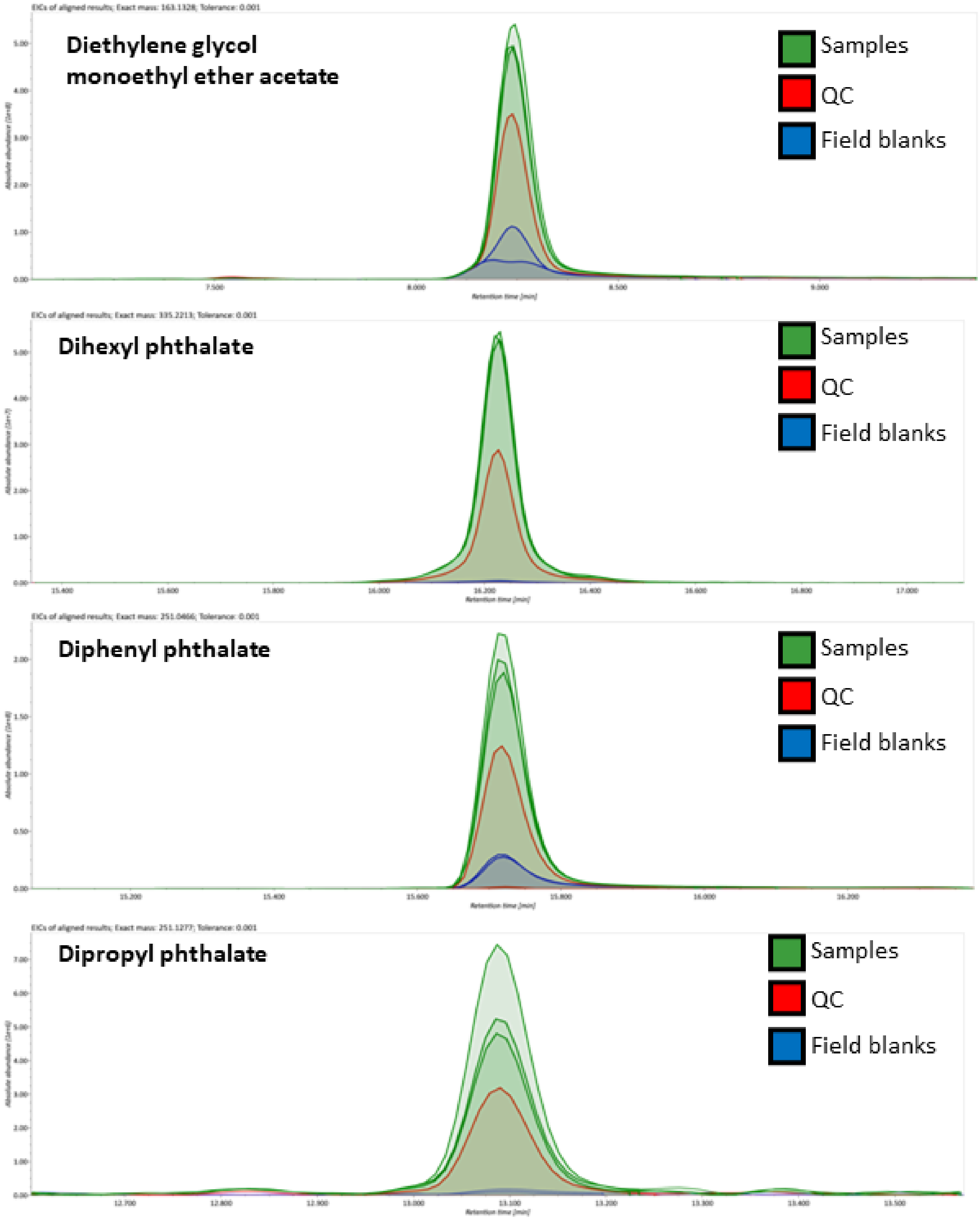

Figure S22. Examples of chromatographic peaks of LC-HRMS annotations in samples (green), QC (red) and field blanks (blue).

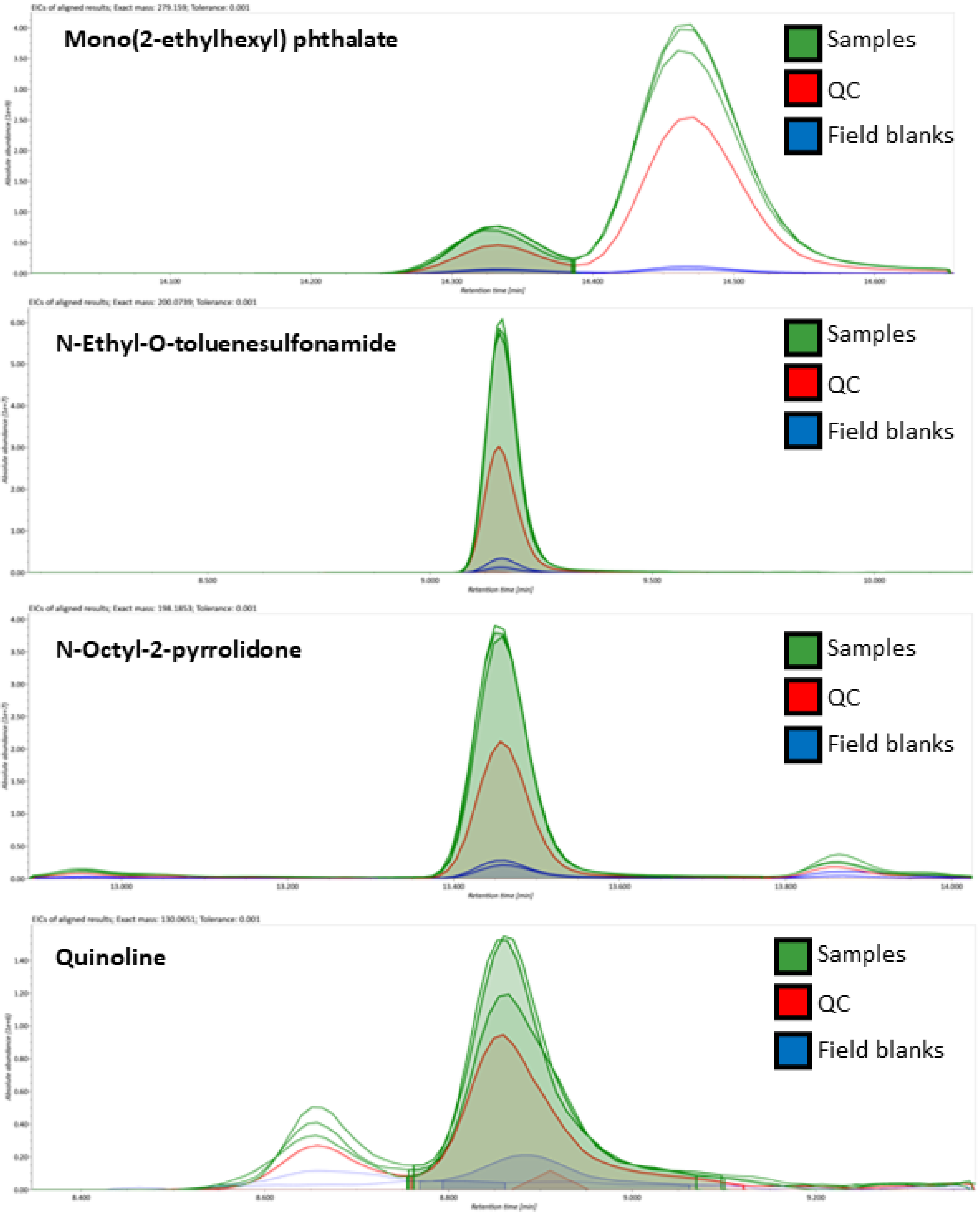

Figure S22. Examples of chromatographic peaks of LC-HRMS annotations in samples (green), QC (red) and field blanks (blue).

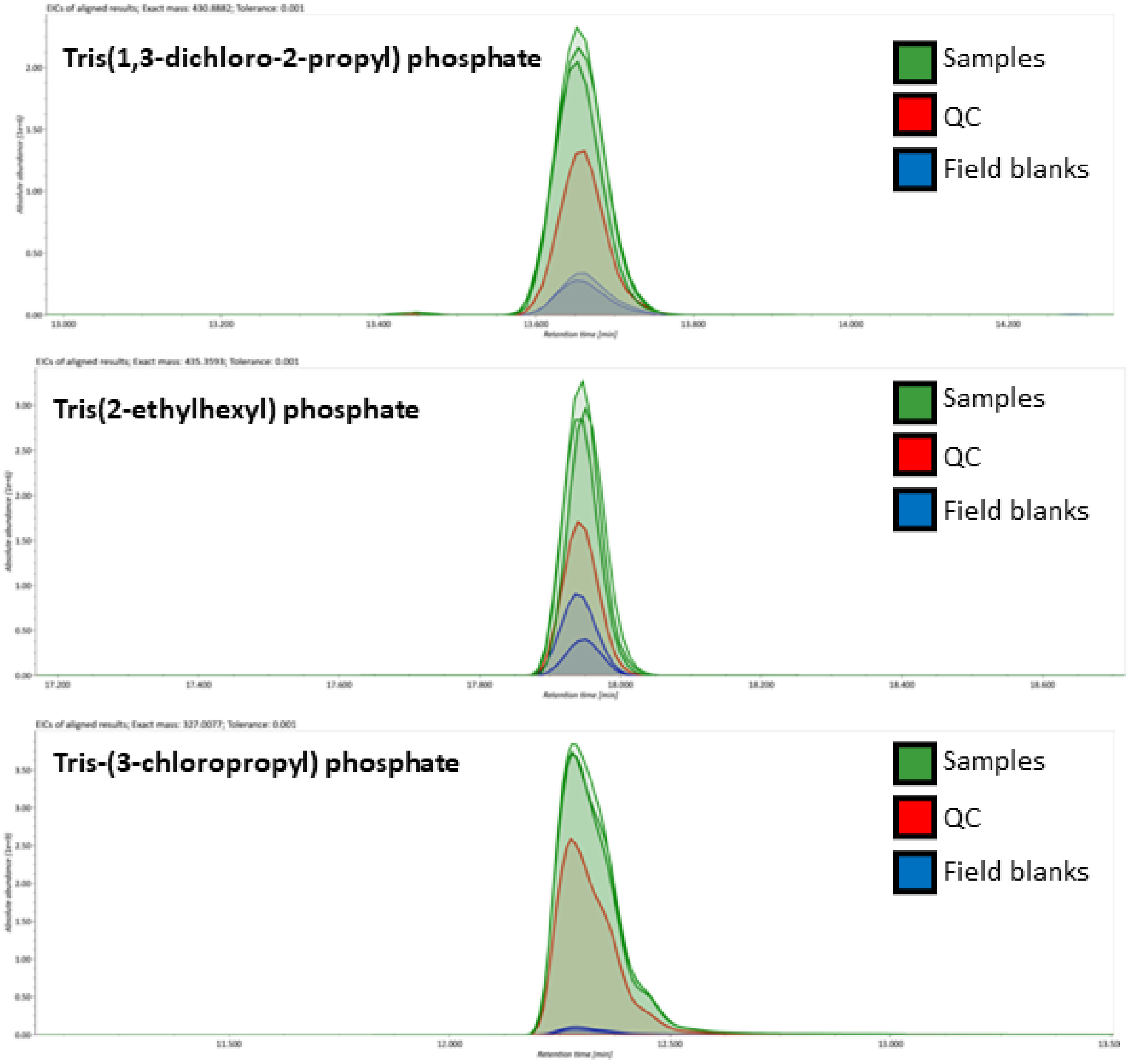

Figure S22. Examples of chromatographic peaks of LC-HRMS annotations in samples (green), QC (red) and field blanks (blue).

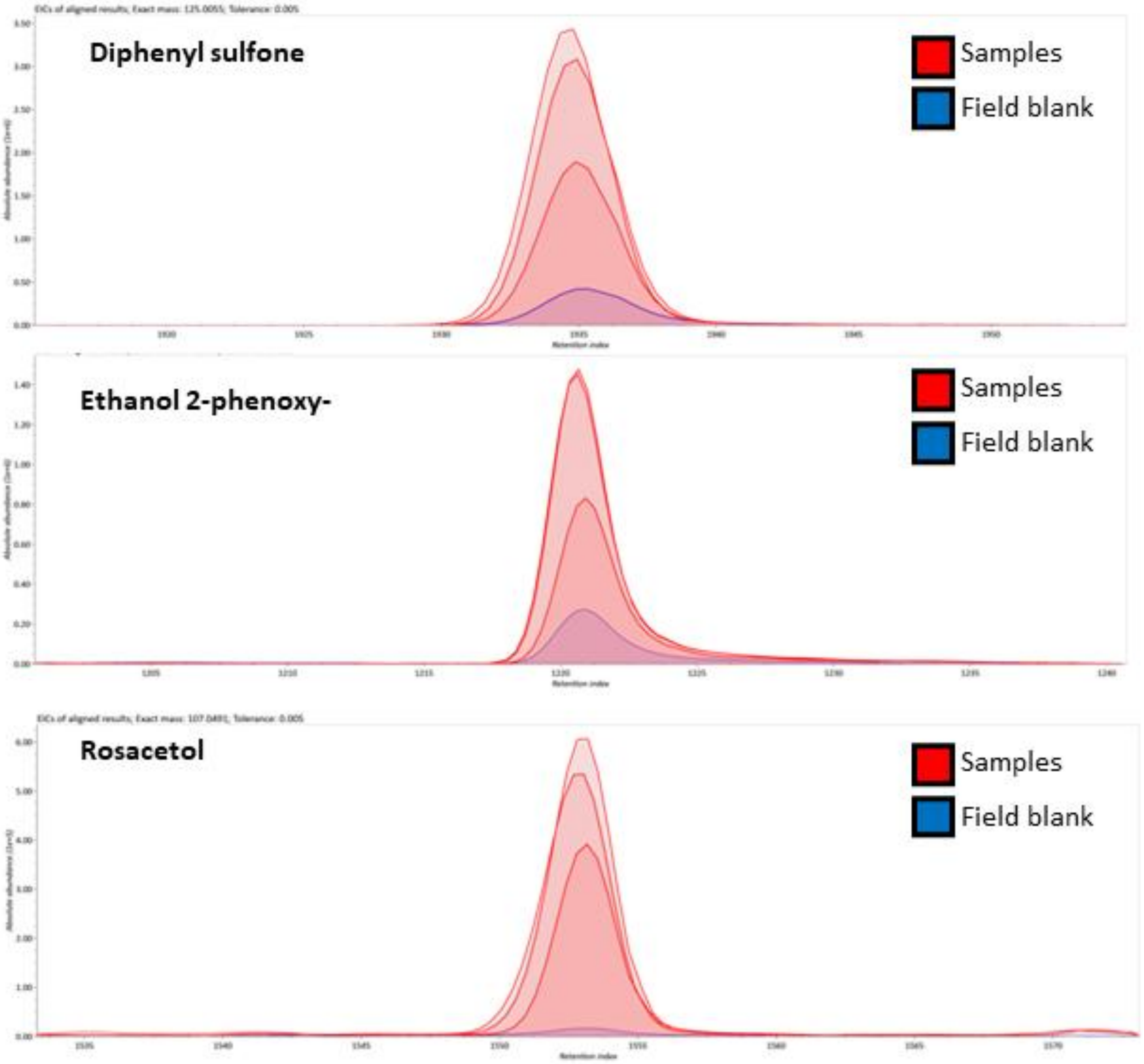

Figure S23. Examples of chromatographic peaks of GC-HRMS annotations in samples (red) and field blank (blue).

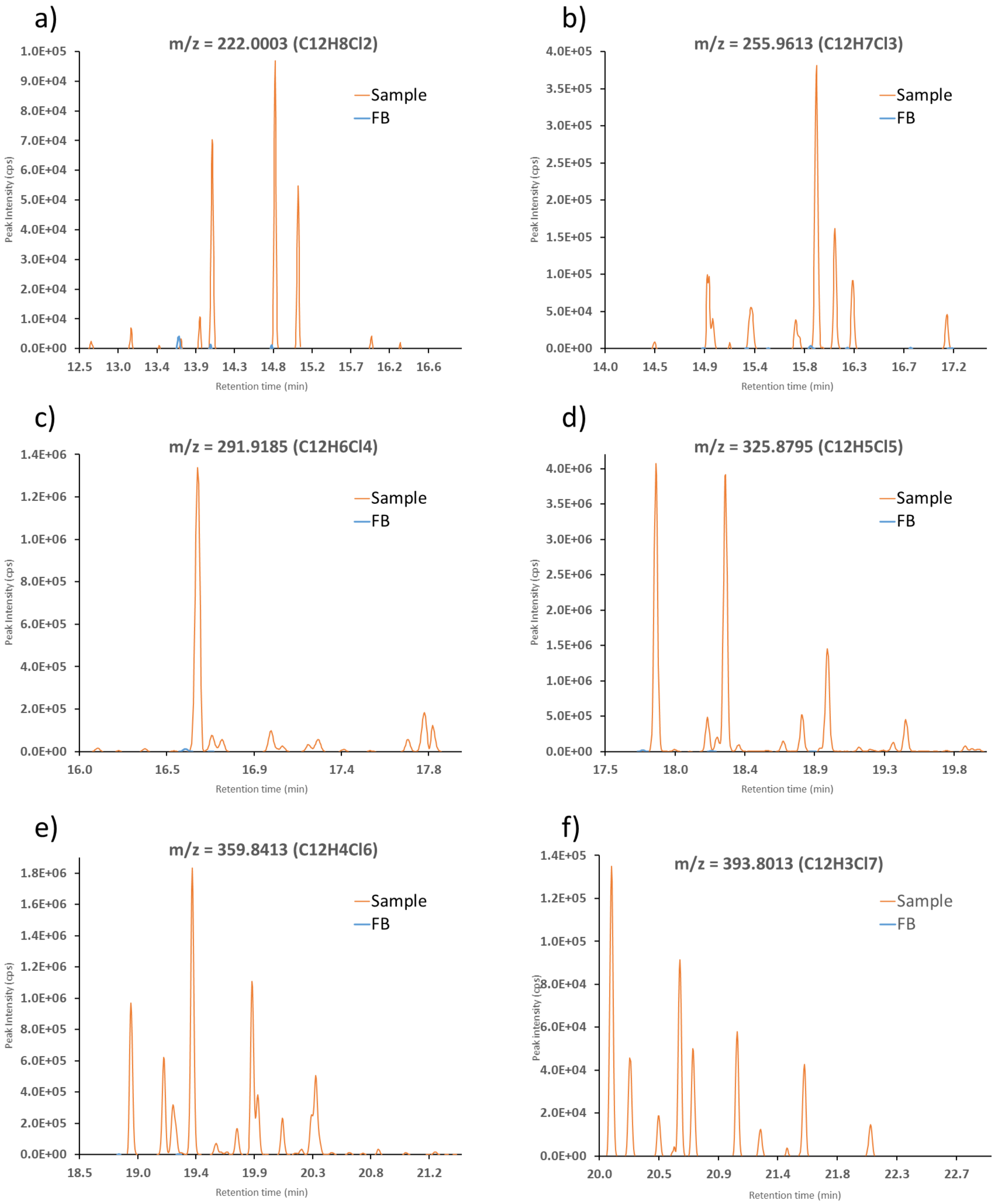

Figure S24. Overlaid extracted ion chromatograms for a sample and its corresponding field blank (FB) from deployment 1, showing the characteristic PCB masses: a) m/z = 222.0003 ($C_{12}H_8Cl_2$), b) m/z = 255.9613 ($C_{12}H_7Cl_3$), c) m/z = 291.9185 ($C_{12}H_6Cl_4$), d) m/z = 325.8795 ($C_{12}H_5Cl_5$), e) m/z = 359.8413 ($C_{12}H_4Cl_6$), and f) m/z = 393.8013 ($C_{12}H_3Cl_7$).

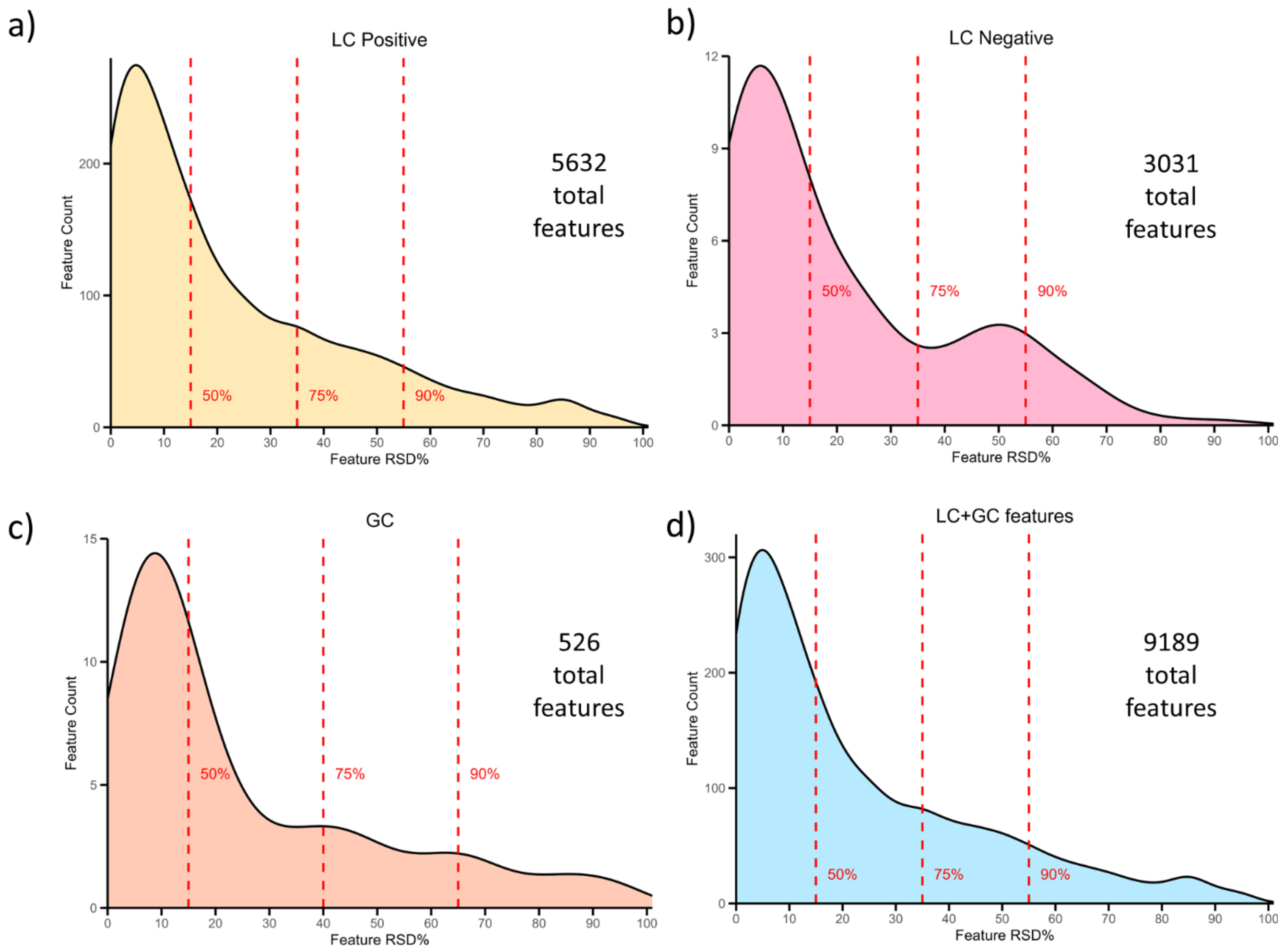

Figure S25. Density plots of the relative standard deviations (RSD%) between features extracted from triplicate PDMS foams deployed in the same location, with individual extractions performed by: a) LC (ESI+), b) LC (ESI-), c) GC, and d) combined extraction (all methods). No signal correction or standardization was applied. Horizontal red dashed lines mark the cumulative RSD values at 50%, 70%, and 90% of the total number of features, providing a visual reference for feature distribution across the analyses.

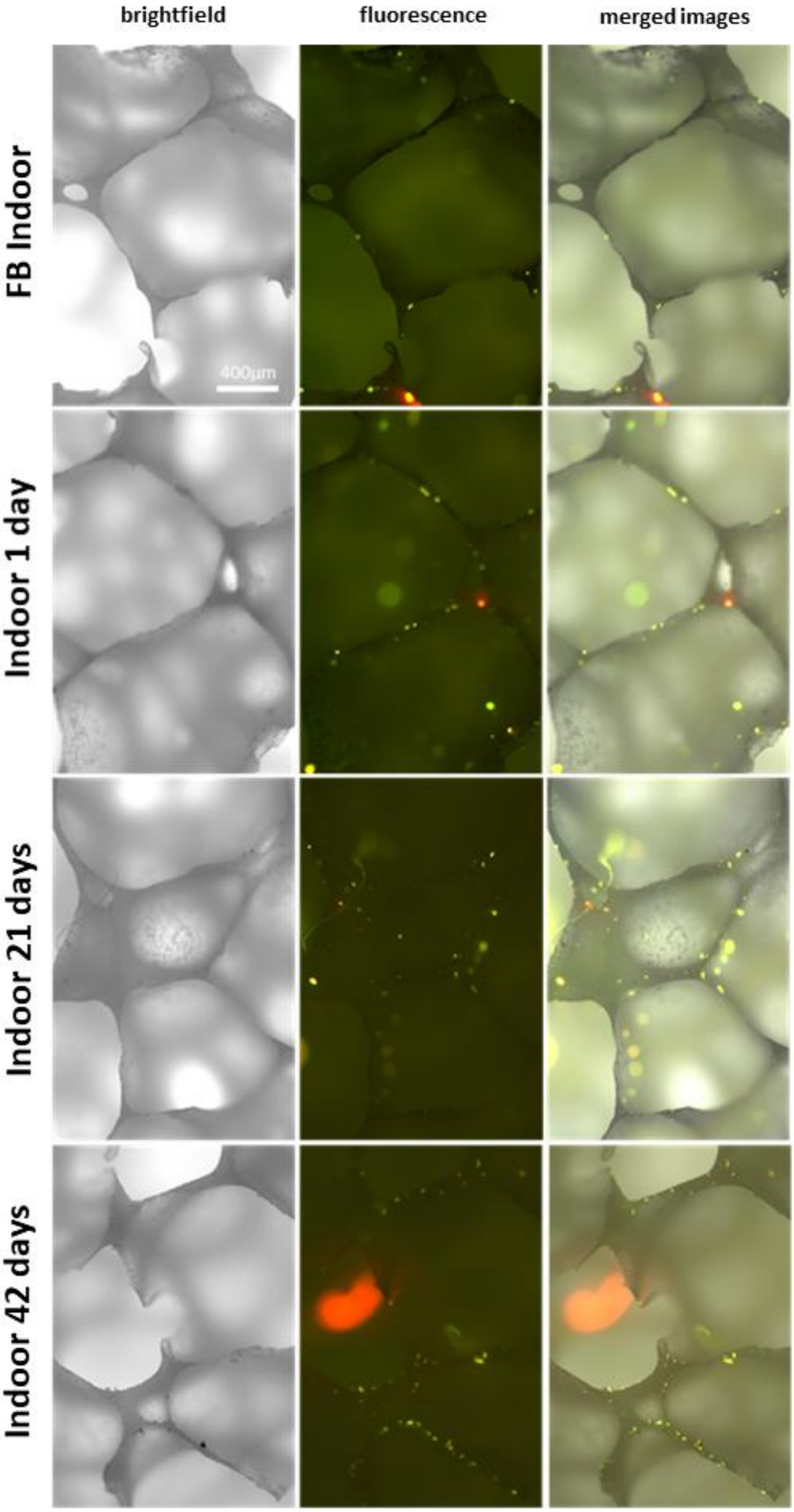

Figure S26. Microscopy images were used to demonstrate PM capture by PDMS foams using brightfield and fluorescent images to visualize the difference in particles captured by field blank PDMS and indoor-deployed PDMS during 1, 21, and 42 days.

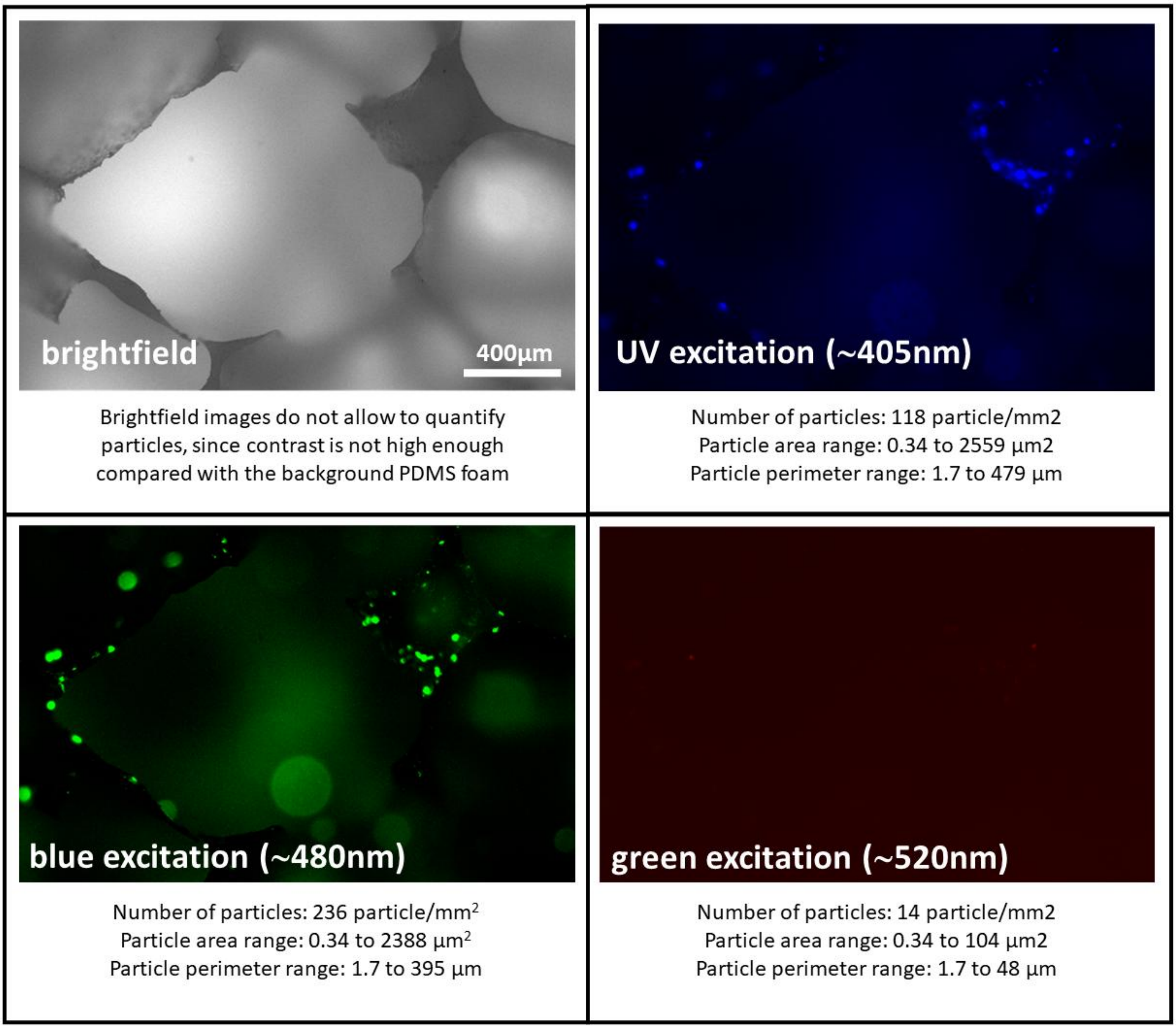

Figure S27. Brightfield and fluorescence images of the Indoor 42 days sample (Deployment 2), comparing imaging techniques and excitation wavelengths for optimal particle quantification. Blue excitation (480 nm), which induces green fluorescence in PM, was identified as the most effective method for accurately quantifying captured particles across a wider size and composition range.

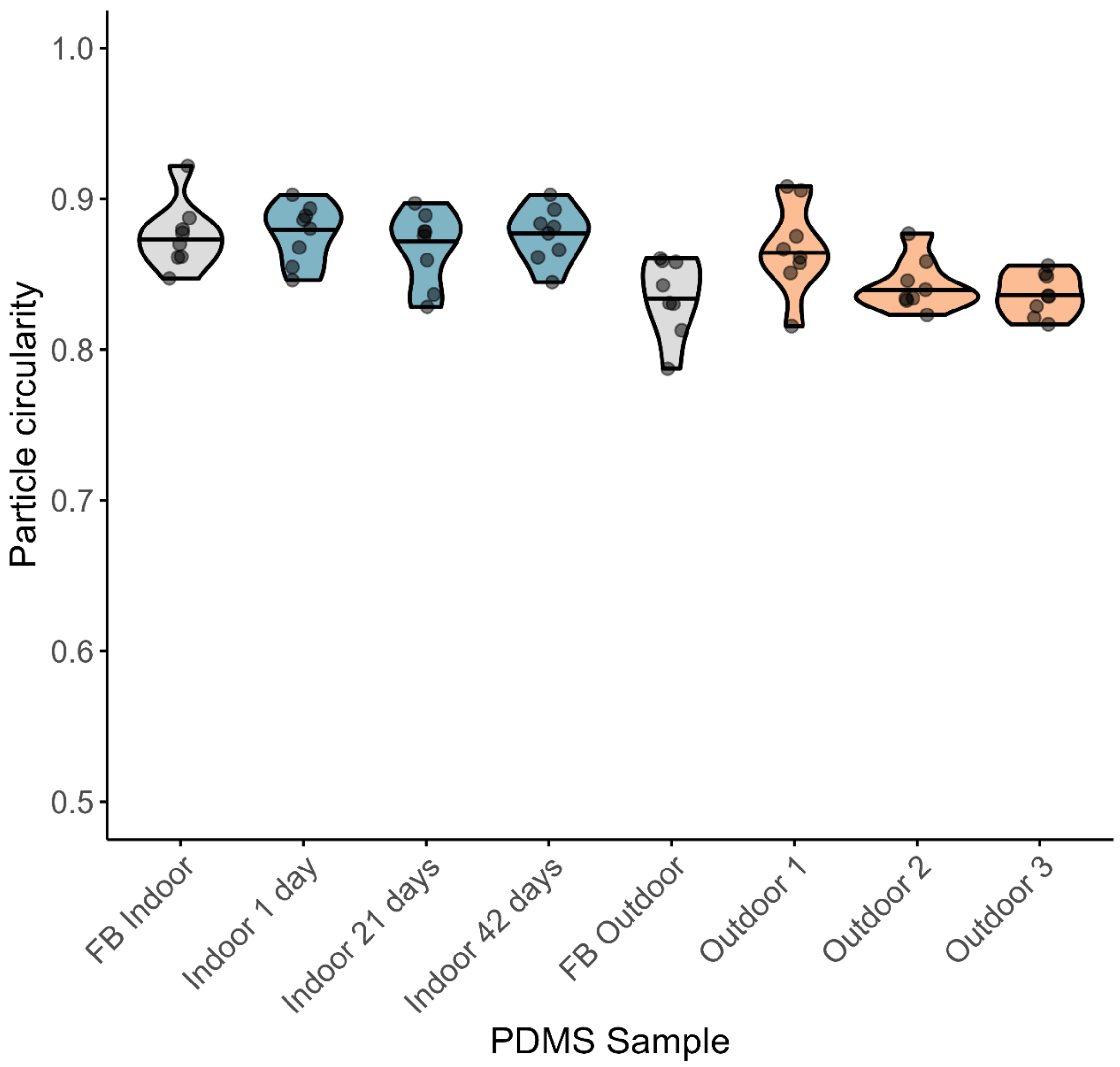

Figure S28. Quantification of the shape of particle captured in indoor and outdoor PDMS foams using circularity (closer to 1 is closer to perfect circle). Each sample is represented by a boxplot of 1 to 3 replicate of 8 independent measurements per foam and statistical analysis was performed using one-way ANOVA with a post-hoc Tukey-Kramer test (α = 0.05). No significant differences were observed between the different samples.

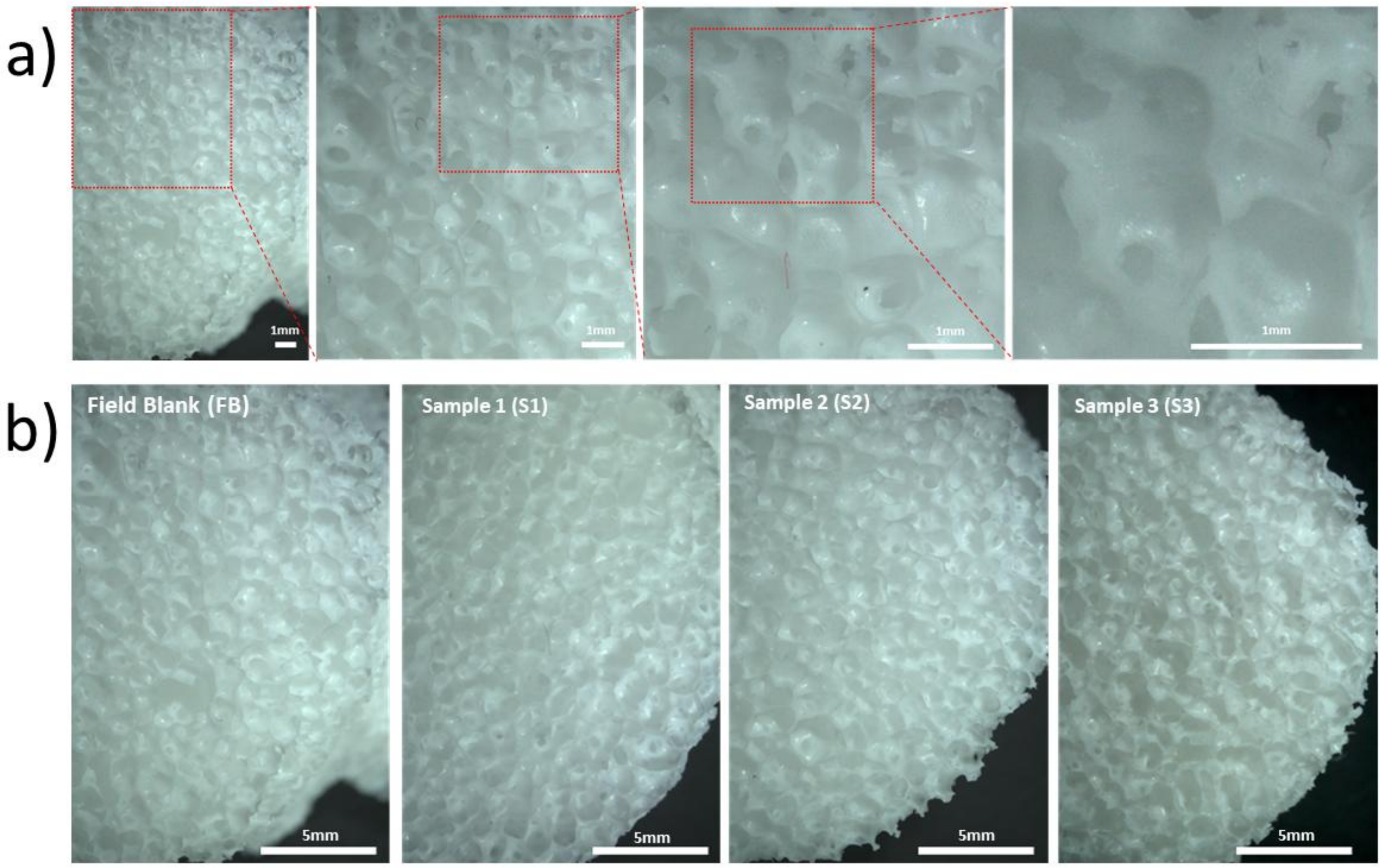

Figure S29. Characterization of PDMS foam structural modifications after 42 days of outdoor exposure. (a) Stereomicroscope magnification used to measure alveoli properties. (b) Stereomicroscope images of PDMS foams, including a non-deployed field blank and samples (1, 2, and 3) deployed outdoors for 42 days.

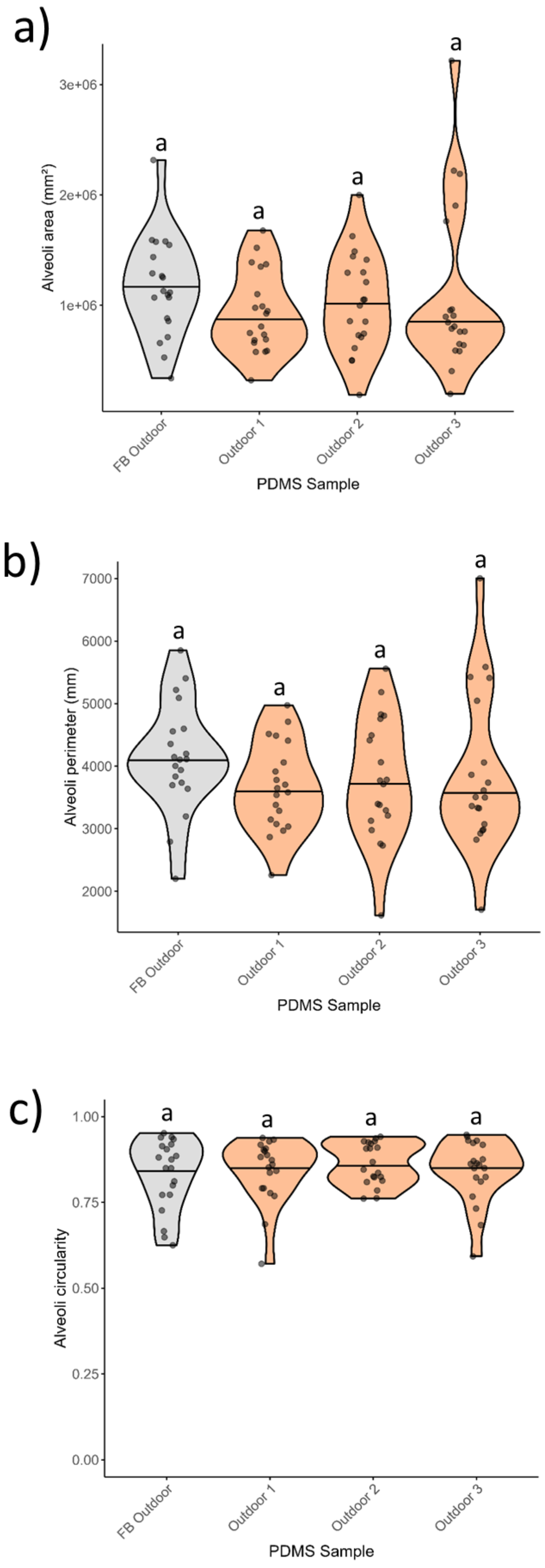

Figure S30. Alveoli parameters were measured to evaluate PDMS stability over time and deployment conditions. a) Boxplots depicting alveoli area (mean ≈ 1 mm^2), b) perimeter (mean ≈ 4 mm), and c) circularity (mean ≈ 85%), based on measurements from 20 alveoli per sample. Statistical comparisons were performed using Kruskal-Wallis/Dunn tests (α = 0.05). Significant differences in the statistical test are indicated with different letters on top of each boxplot.

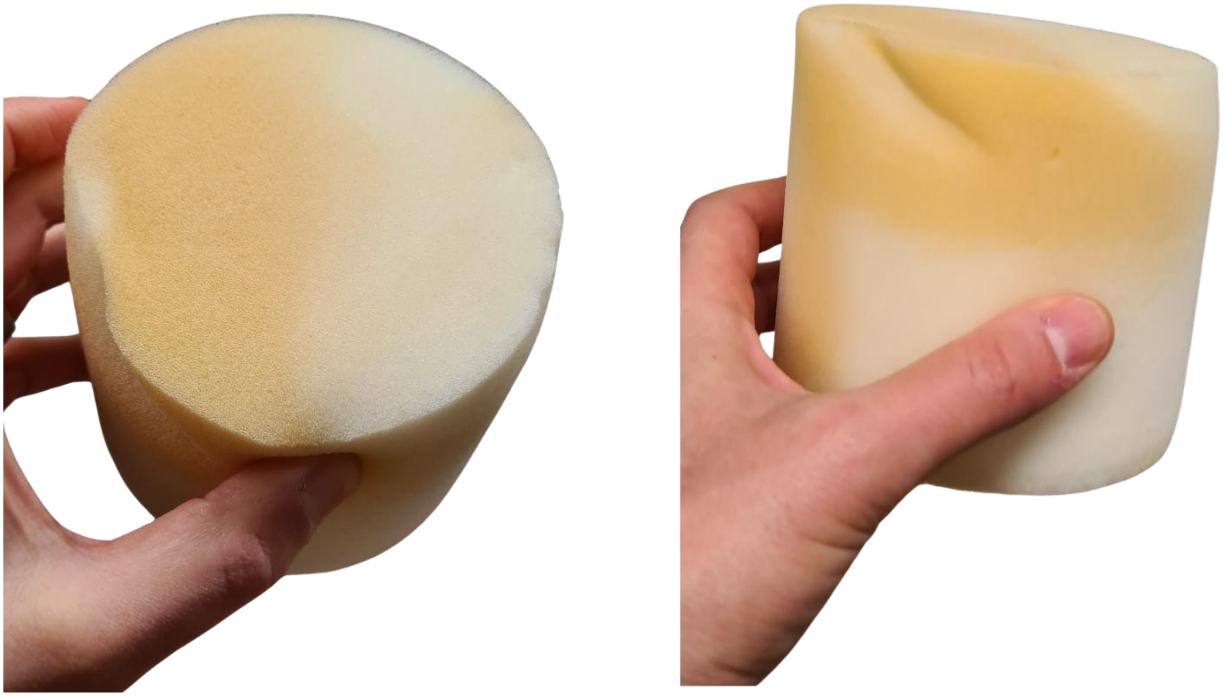

Figure S31. Photographs of polyurethane foam (PUF) disks showing partial surface oxidation after field deployment under real-world conditions.

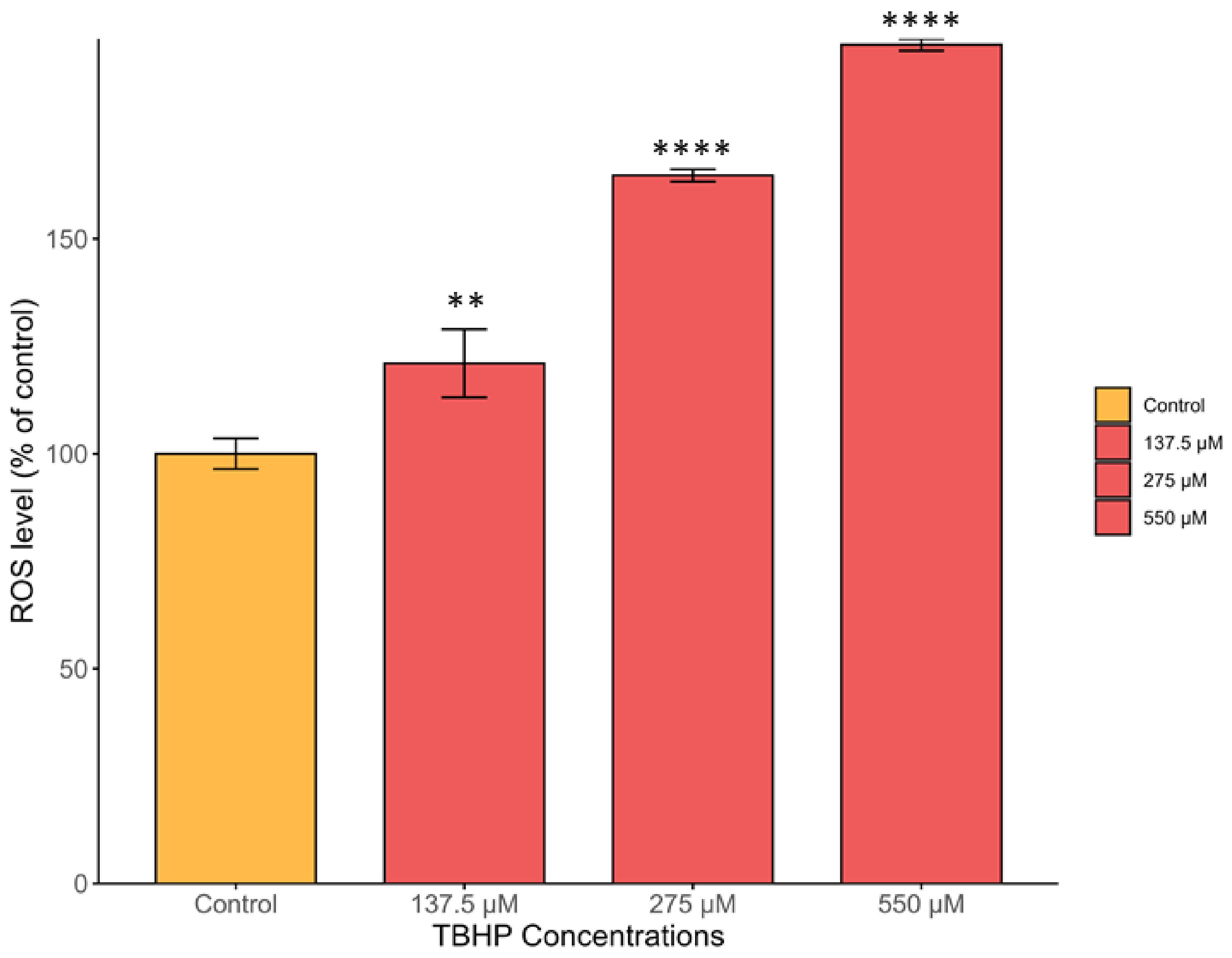

Figure S32. Intracellular ROS levels in human lung fibroblasts exposed to TBHP. Intracellular ROS levels were measured using a fluorescence-based ROS detection method after exposure to increasing concentrations of TBHP, a known oxidative stress inducer used as a positive control for ROS production. Data are expressed as mean ± Standard Error of the Mean (SEM) (n = 3 per treatment). One-way ANOVA followed by Dunnett's test was performed to compare each treatment group with the control. Statistical significance was defined as ns (not significant), *p < 0.05, **p < 0.01, ***p < 0.001, and ***p < 0.0001.

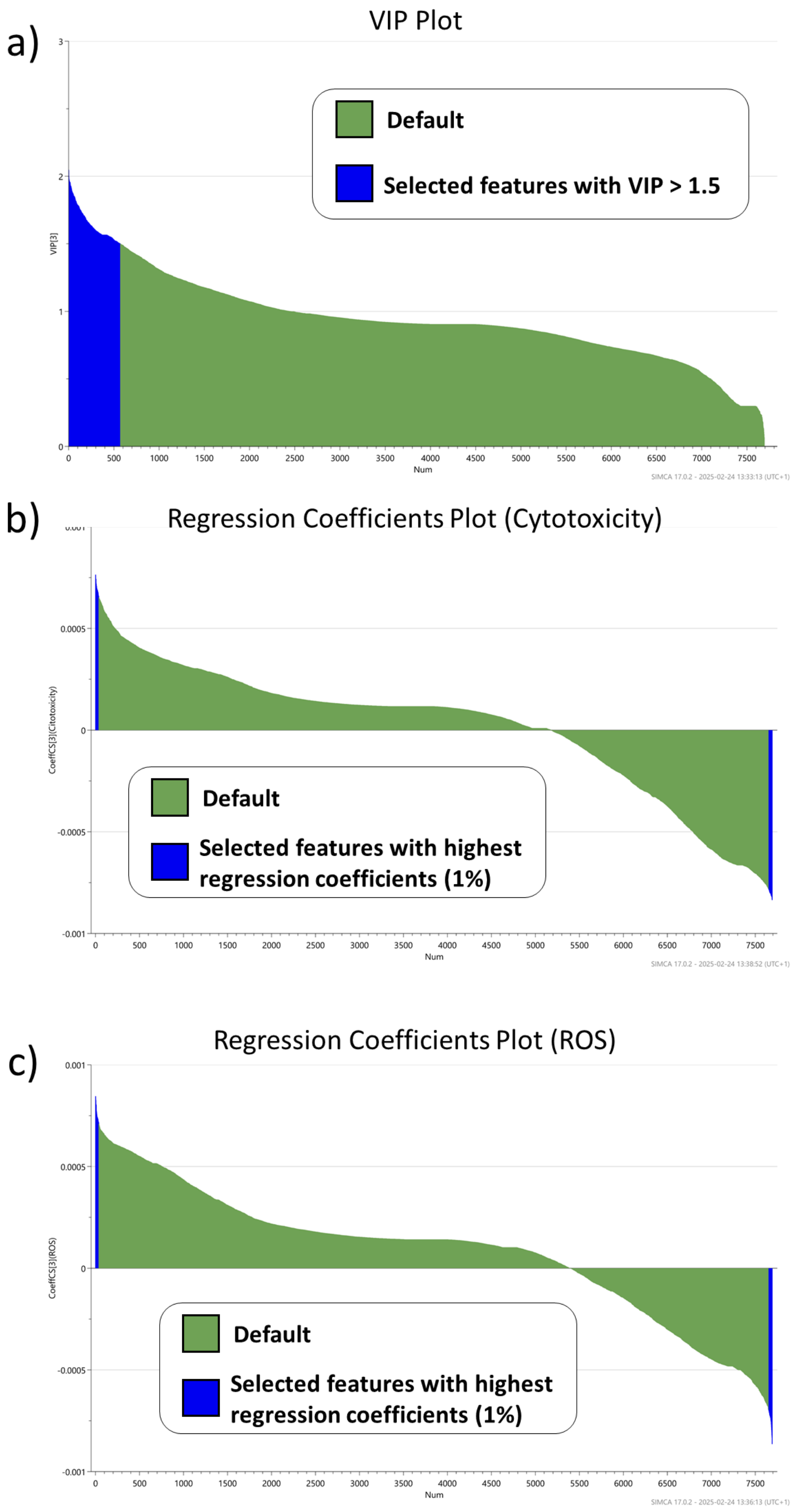

Figure S33. Statistical analysis of extracted features to assess correlations with cytotoxicity and ROS levels. Features were prioritized based on: a) VIP score > 1.5, b) the top 1% of features with the highest regression coefficients for cytotoxicity, and c) the top 1% of features with the highest regression coefficients for ROS levels. Only features meeting both criteria—VIP > 1.5 and among the top 1% of regression coefficients (for either cytotoxicity or ROS)—were selected.
